# A Water-Soluble Aggregation-Induced Emission Photosensitizer with Intrinsic Antibacterial Activity as an Antiplanktonic and Antibiofilm Therapeutic Agent

**DOI:** 10.1021/acs.jmedchem.5c00403

**Published:** 2025-04-05

**Authors:** Cheung-Hin Hung, Ka Hin Chan, Wai-Po Kong, Ruo-Lan Du, Kang Ding, Zhiguang Liang, Yong Wang, Kwok-Yin Wong

**Affiliations:** State Key Laboratory of Chemical Biology and Drug Discovery, Department of Applied Biology and Chemical Technology, The Hong Kong Polytechnic University, Kowloon, Hong Kong, China

## Abstract

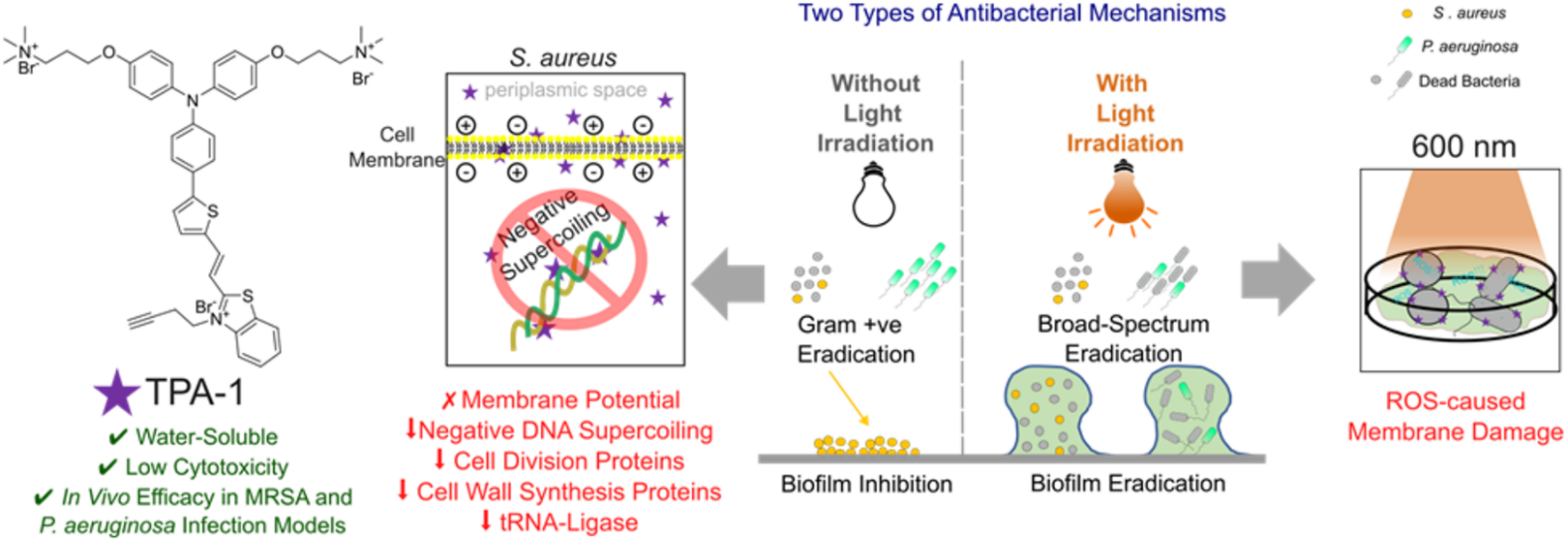

Photosensitizers (PSs) with aggregation-induced emission (AIE) properties have gained popularity for treating bacterial infections. However, most AIE PSs have a poor water solubility and low selectivity, limiting their applications in biological systems. Herein, we report a water-soluble and bacteria-targeting AIE PS that exhibits minimum cytotoxicity toward human cells with and without light irradiation. Acting as a narrow-spectrum antibacterial agent without light irradiation, TPA-1 eradicates planktonic *Staphylococcus aureus* and inhibits biofilm formation by targeting the *S. aureus* membrane, inhibiting the supercoiling activity of *S. aureus* DNA gyrase, and causing the downregulation of multiple essential proteins. Upon light irradiation, TPA-1 generates reactive oxygen species (ROS) that cause membrane damage, resulting in excellent antiplanktonic and antibiofilm activities against *S. aureus* and *Pseudomonas aeruginosa*, significantly reducing the number of viable bacteria in biofilms and promoting wound healing *in vivo*.

## 1 Introduction

The treatment of bacterial infections has long been a clinical challenge. Bacteria can encase themselves in self-produced extracellular polymeric substances (EPS), generating a biofilm.^1^ EPS, which includes extracellular DNA, polysaccharides, lipids, and proteins, protects bacteria from the host immune system and antibiotics.^1−4^ Biofilm-related infections are reportedly the leading cause of chronic and recurrent wounds.^5−7^ Clinical treatment of such infections usually involves physical debridement and high-dose antibiotics,^8^ which tends to cause pain and discomfort to patients. Moreover, such treatments have become less effective over the years owing to the emergence of antibiotic resistance.^9,10^

Photodynamic therapy (PDT) is a promising alternative to antibiotics for the treatment of bacterial infections owing to its rapid eradication rate and ability to overcome antimicrobial resistance.^11^ PDT combines a photosensitizer (PS) with light irradiation, generating toxic reactive oxygen species (ROS) that destroy essential bacterial components.^12,13^ Several aggregation-induced emission (AIE) PSs have been developed to treat bacterial infections, demonstrating high ROS generation ability and unique photophysical properties that can prevent aggregation-caused quenching.^14,15^ Nevertheless, most AIE PSs are insoluble in water, potentially leading to high cytotoxicity due to the formation of aggregates in biological systems.^16−23^ Although some AIE PSs have been fabricated as positively charged nanoparticles, resulting in good water dispersion, they tend to accumulate in human cells, increasing their toxicity with and without irradiation.^24−26^ Designing a water-soluble AIE PS could overcome the toxicity issue in bioapplications. Furthermore, many reported AIE PSs exhibit good antiplanktonic activity but are less likely to eradicate bacterial biofilms.^27−36^

**Figure 1:**
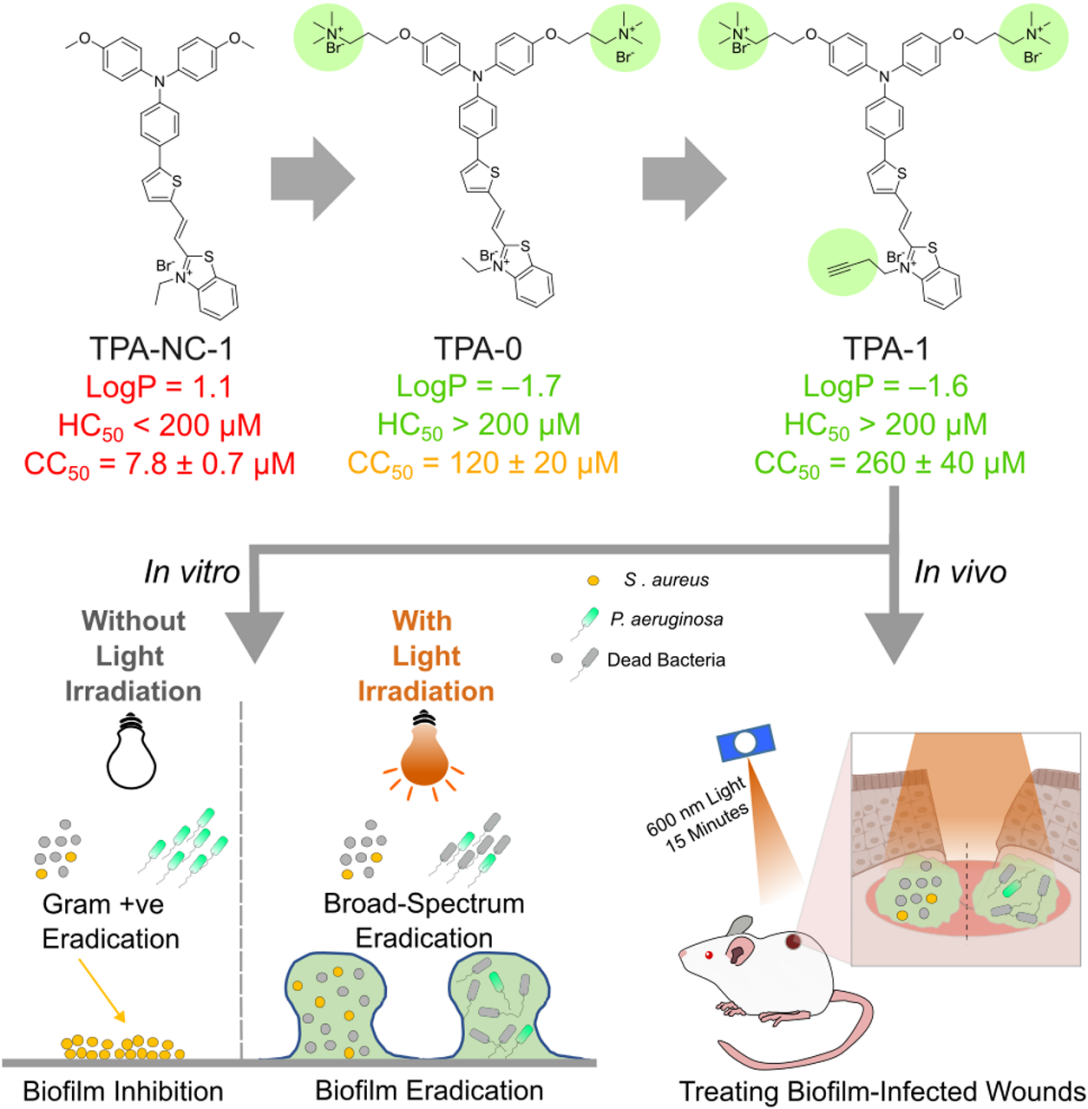
Molecular design of triphenylamine derivative TPA-1 and an illustration of its antibacterial activity.

Herein, we report the design and synthesis of a novel water-soluble AIE PS for antibacterial treatment. The triphenylamine derivative, TPA-1, exhibits excellent water solubility and bacteria-targeting ability as well as low toxicity toward human cells. Additionally, TPA-1 exhibits intrinsic antibacterial activity against planktonic *Staphylococcus aureus* and can inhibit biofilm formation even without light irradiation. Upon light irradiation at 600 nm, TPA-1 exhibits broad-spectrum antibacterial activity against planktonic and biofilm bacteria. Moreover, TPA-1 is effective in treating wounds infected with methicillin-resistant *S. aureus* (MRSA) and *Pseudomonas aeruginosa* biofilms *in vivo* (Figure 1).

## 2 Results and Discussion

### 2.1 Molecular Design of Triphenylamine (TPA) Derivatives

**Figure 2:**
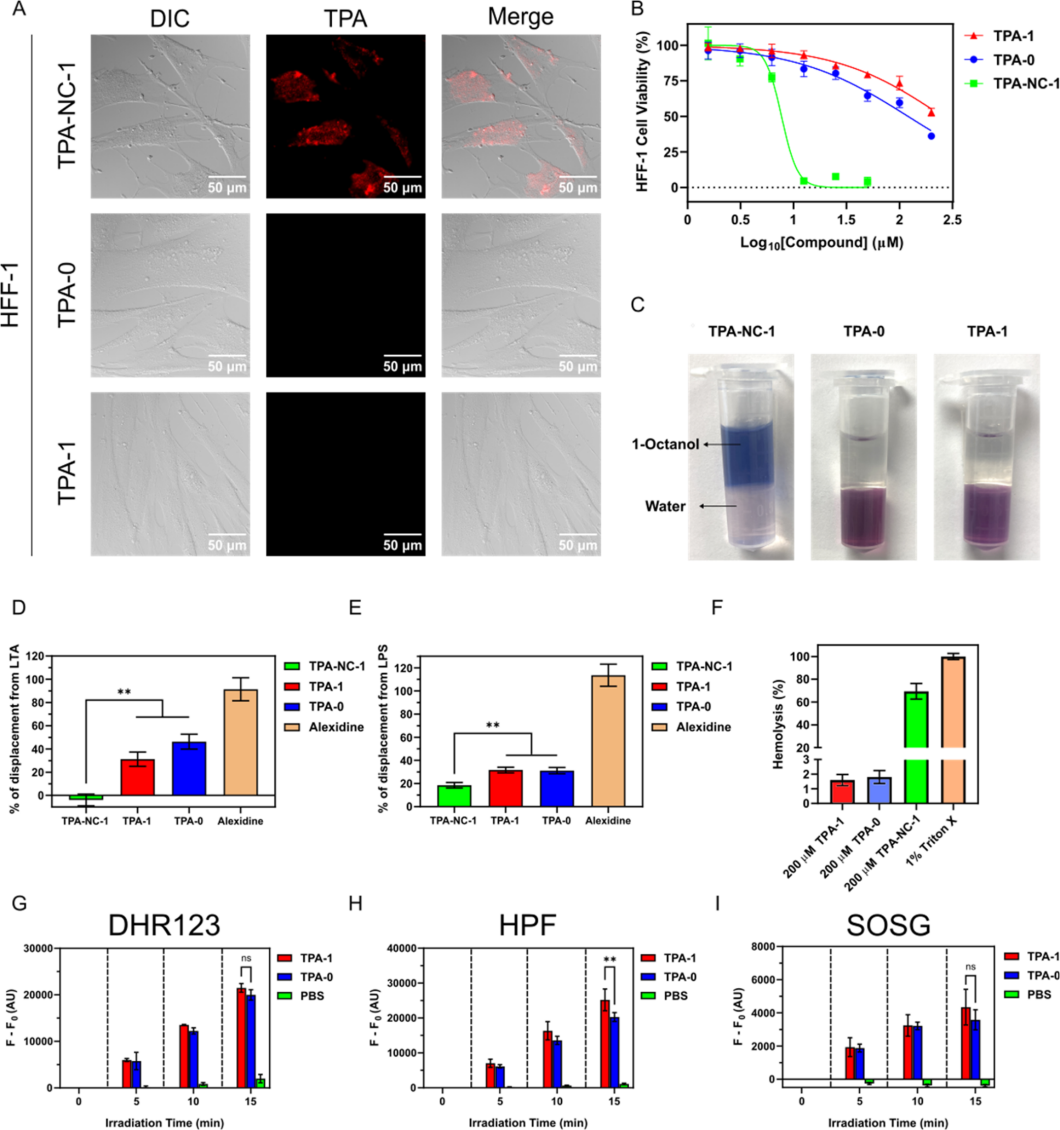
(A) Differential interference contrast (DIC) and fluorescence images of human foreskin fibroblast (HFF-1) cells with TPA-NC-1 (10 μM), TPA-0 (10 μM), and TPA-1 (10 μM). The excitation wavelength for the TPA compounds (TPA) was 550 nm. Emission wavelengths were collected from 590 to 670 nm. (B) HFF-1 cell viability after incubation with TPA compounds for 24 h. (C) Photographs of TPA compounds in 1-octanol/water (1/1) mixture. (D) *S. aureus* LTA and (E) *P. aeruginosa* LPS-binding assay results of TPA compounds (10 μM) and alexidine (positive control, 10 μg/mL) using BODIPY TR Cadaverine. (F) Hemolysis rate of human erythrocytes treated with TPA compounds. ROS generation assay results of TPA-0 (10 μM) and TPA-1 (10 μM) under irradiation with 600 nm light (60 mW/cm^2^) for 15 min with (G) DHR123 used as a nonspecific ROS detection probe. (H) HPF used as a hydroxyl radical detection probe. (I) SOSG used as a singlet oxygen detection probe. Data are presented as the mean ± SD, with *n* = 3 per group. ** *p*-Value <0.01, ns (not significant) *p*-value >0.05.

TPA-NC-1 (Figure 1) is an AIE PS with a donor−π–acceptor structure that is infused with heavy atoms (sulfur) to facilitate efficient intersystem crossing.^37−39^ It is known that TPA-NC-1 possesses a high ROS generation ability.^39^ However, like other AIE PSs, TPA-NC-1 is insoluble in water and is toxic to human cells. As shown in the fluorescence image in Figure 2A, TPA-NC-1 strongly binds to human foreskin fibroblast (HFF-1) cells. The 50% cytotoxicity concentration (CC_50_) of TPA-NC-1 against HFF-1 cells was found to be as low as 7.8 ± 0.7 μM, indicating its high toxicity toward human cells (Figure 2B). To exploit the photosensitizing abilities of TPA-NC-1 while dodging its shortcomings, we designed two analogs by attaching two quaternary ammonium groups on the triphenylamine core (TPA-0) and modifying the substituent on the benzothiazolium group (TPA-1). The effects of adding quaternary ammonium groups were demonstrated by TPA-0. TPA-0 has a log*P* value of −1.7 (*c *log* P* = −0.9), which is lower than that of TPA-NC-1 (log* P* = 1.1, *c *log *P* = 6.8), indicating that the two quaternary ammonium groups enhance the water solubility of the PS (Figure 2C). Furthermore, we hypothesize that the positively charged quaternary ammonium groups would bind to negatively charged lipoteichoic acid (LTA) on Gram-positive bacterial membranes and lipopolysaccharide (LPS) on Gram-negative bacterial membranes. The fluorescent dye BODIPY TR Cadaverine (BTRC) was employed to evaluate the binding of TPA compounds with LTA from *S. aureus* and LPS from *P. aeruginosa*.^40^ The fluorescence signal of BTRC was quenched when bound to LTA or LPS; however, an intense fluorescence signal at 620 nm was generated when BTRC was displaced by LTA- or LPS-binding molecules.^40−42^ As shown in Figure 2D,2E, TPA-0 displaced BTRC more readily from LTA and LPS than TPA-NC-1, indicating that the quaternary ammonium groups increased the level of binding of TPA-0 to bacteria. In addition, the quaternary ammonium groups reduced the affinity of TPA-0 toward human cells. The TPA-0-treated HFF-1 cells did not generate an observable fluorescence signal (Figure 2A). Additionally, the CC_50_ of TPA-0 against HFF-1 cells is 120 ± 20 μM, which is much higher than that of TPA-NC-1 (Figure 2B). Furthermore, the hemolysis rate of human erythrocytes treated with TPA-0 is much lower than that of human erythrocytes treated with TPA-NC-1:200 μM TPA-0 induced <3% hemolysis, while 200 μM TPA-NC-1 induced >60% hemolysis (Figure 2F). Therefore, the addition of quaternary ammonium groups improved the water solubility of the PS but also increased its selectivity toward bacteria and reduced its cytotoxicity toward human cells. Subsequently, we synthesized TPA-1 by modifying the ethyl group on the benzothiazolium group to 1-butyne. Apart from preserving the desirable properties of TPA-0 (Figure 2A–F), TPA-1 exhibited even lower cytotoxicity and higher ROS generation ability than did TPA-0. TPA-1 has a CC_50_ value of 260 ± 40 μM against HFF-1 cells and induces <2% hemolysis at 200 μM.

Next, we conducted ROS generation assays with TPA-0 and TPA-1 using commercial ROS detection probes. Dihydrorhodamine 123 (DHR123), hydroxyphenyl fluorescein (HPF), and Singlet Oxygen Sensor Green (SOSG) were employed as nonspecific ROS, hydroxyl radical, and singlet oxygen indicators, respectively. As shown in Figure 2G–I, the fluorescence signals for all three indicators gradually increased with irradiation time after mixing the ROS probes with TPA-1 or TPA-0, indicating that TPA-1 and TPA-0 could generate type I and II ROS. In addition, TPA-1 showed a slightly higher hydroxyl radical generation rate than did TPA-0. These favorable properties suggested that the antibacterial activity TPA-1 warranted further study.

### 2.2 Intrinsic Antiplanktonic and Antibiofilm Properties of TPA-1 without Light Irradiation

**Figure 3:**
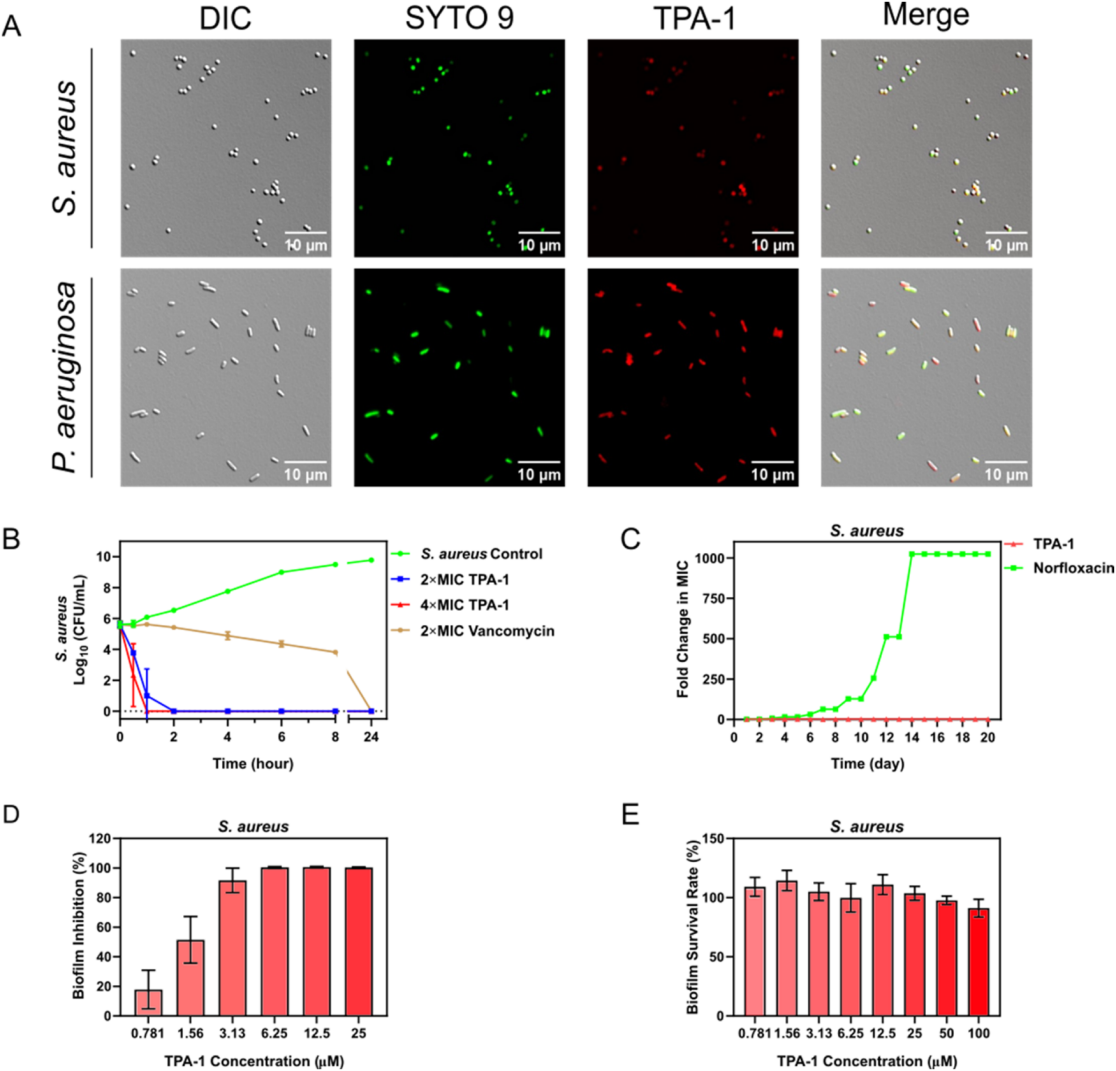
(A) DIC and fluorescence images of *S. aureus* and *P. aeruginosa* treated with SYTO 9 (2.5 μM) and TPA-1 (20 μM). SYTO 9 is a commercial stain that can bind to bacterial DNA. The excitation wavelengths for SYTO 9 and TPA-1 were 490 and 550 nm, respectively. Emission wavelengths were collected from 500 to 550 nm for SYTO9 and from 590 to 670 nm for TPA-1. (B) Time–kill kinetics assay results of 2 × MIC TPA-1 (6.25 μM), 4 × MIC TPA-1 (12.5 μM), and 2 × MIC vancomycin (1.25 μM) against *S. aureus*. *n* = 3 per group. (C) Resistance development assay results of TPA-1 and norfloxacin (positive control) against *S. aureus*. This graph represents one of two independent experiments. The second replicate is shown in Figure S34. (D) Biofilm inhibition rate of TPA-1 against *S. aureus* without light irradiation. *n* = 3 per group. (E) Mature biofilm survival rate of *S. aureus* after incubation with TPA-1 in the dark for 24 h. *n* = 3 per group. Data are presented as the mean ± SD.

Fluorescence imaging validated that TPA-1 could label *S. aureus* and *P. aeruginosa*, as shown in Figure 3A. Additionally, upon excitation at 550 nm, a fluorescence signal was generated by bacteria treated with TPA-1 but not by those treated with phosphate-buffered saline (PBS), indicating that the fluorescence signal was generated by the specific binding of TPA-1 to bacteria (Figure S35). The minimum inhibitory concentration (MIC) value of TPA-1 against *S. aureus* (ATCC 29213) and MRSA (BAA 41) was 3.125 μM, while no inhibition was observed against *P. aeruginosa* (ATCC 27853) at concentrations of ≤ 100 μM (Table S1). The time-kill kinetics revealed that treatment with TPA-1 could completely eradicate *S. aureus* within 2 h at 2 × MIC, which was more rapid than vancomycin at 2 × MIC (Figure 3B). Further, we conducted the resistance development assay to assess the antibacterial ability of TPA-1 against *S. aureus* across different bacterial passages (Figure 3C). The initial MIC values for TPA-1 and norfloxacin against *S. aureus* were 3.125 and 0.5 μM, respectively. After treatment for 20 days, the MIC value of TPA-1 against *S. aureus* increased 2-fold, while that of norfloxacin increased 1024-fold, indicating that consecutive treatment with TPA-1 did not lead to the development of significant resistance in *S. aureus*. Additionally, treatment with TPA-1 at 1 × MIC (3.125 μM) inhibited >90% of *S. aureus* biofilm formation, reaching >99.9% at ≥ 6.25 μM (Figure 3D). However, TPA-1 had only limited ability to eradicate mature biofilms without light irradiation (Figure 3E). Collectively, these results suggested that TPA-1 exhibited high antiplanktonic and biofilm inhibition abilities against *S. aureus*, even without light irradiation.

### 2.3 Intrinsic Antibacterial Mechanisms of TPA-1 without Light Irradiation

**Figure 4:**
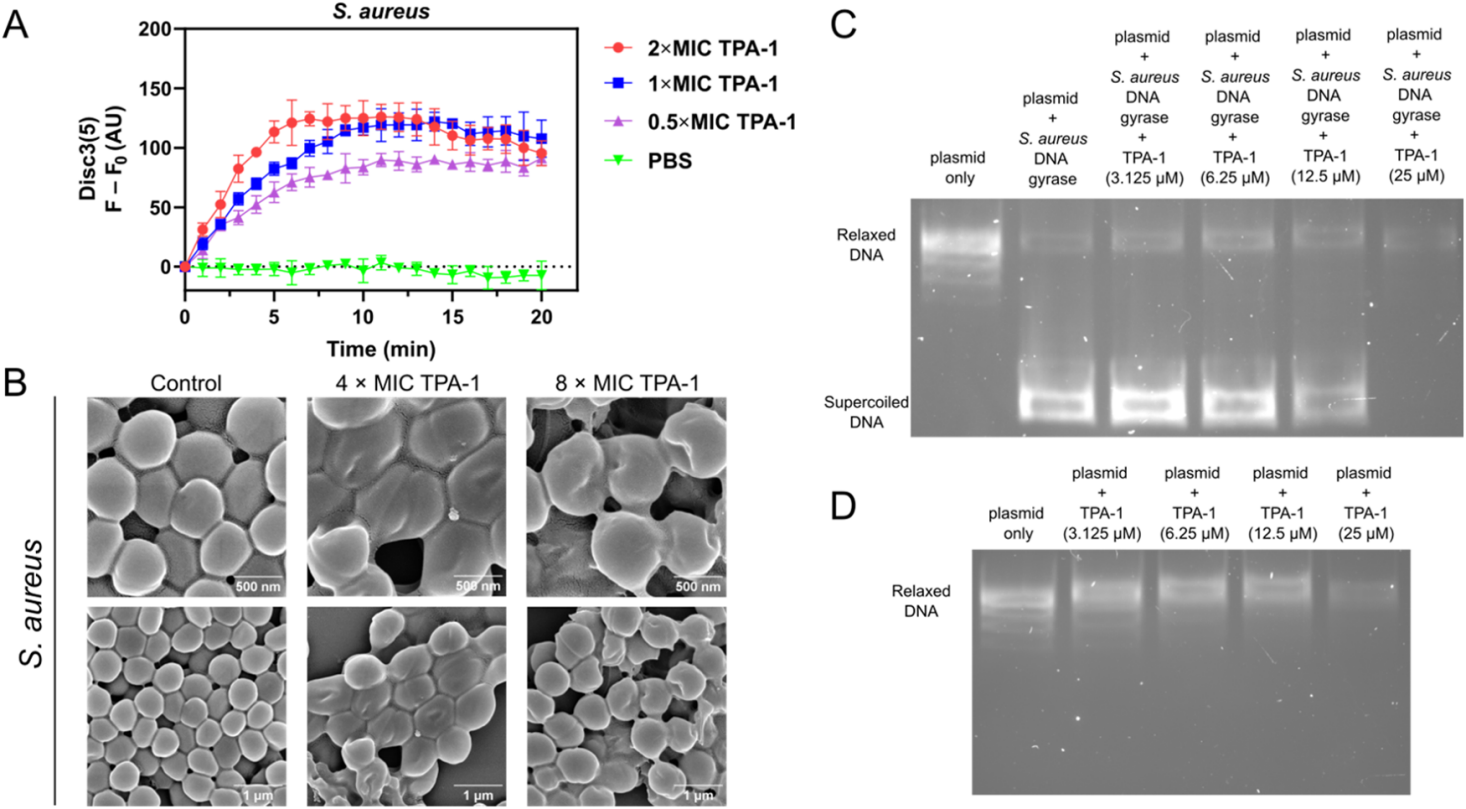
(A) Release of Disc3(5) dye from *S. aureus* with an increasing TPA-1 concentration as a result of membrane depolarization. Data are presented as the mean ± SD, with *n* = 3 per group. (B) Scanning electron microscopy (SEM) images of *S. aureus* after incubation with 4 × MIC TPA-1 (12.5 μM) and 8 × MIC TPA-1 (25 μM) for 3 h in the dark. (C) DNA gel image (1% agarose) showing the supercoiling inhibition activity of different TPA-1 concentrations against *S. aureus* DNA gyrase. (D) DNA gel image (1% agarose) of different concentrations of TPA-1 mixed with the substrate plasmid (pBR322) in the *S. aureus* DNA gyrase assay was used as a negative control.

To determine the antibacterial mechanism of TPA-1, we investigated the effects of TPA-1 on the *S. aureus* cell membrane given that positively charged TPA-1 may depolarize the *S. aureus* cell membrane and affect its integrity. We conducted the membrane depolarization assay using Disc3(5), a fluorescent dye that can accumulate in polarized bacteria cells.^43^ The fluorescence signal of Disc3(5) is quenched upon accumulation in polarized cells, but it can be regenerated once the cells are depolarized and Disc3(5) is released from the cells. As shown in Figure 4A, treatment with TPA-1 increased the fluorescence signal of Disc3(5) in *S. aureus* in a concentration-dependent manner, indicating that treatment with TPA-1 could depolarize its bacterial membrane. Additionally, scanning electron microscopy (SEM) revealed that treatment with TPA-1 could cause collapse of the *S. aureus* cell surface, suggesting damage to the bacterial membrane (Figure 4B). Apart from affecting the integrity of *S. aureus* membrane, TPA-1 demonstrated a DNA-binding affinity toward *S. aureus* genomic DNA (Figure S37). Treatment of *S. aureus* genomic DNA with TPA-1 caused an upshift in the DNA band in the DNA gel image. Furthermore, TPA-1 inhibited the negative supercoiling activity of *S. aureus* DNA gyrase, a property that is rarely reported for other antibacterial PSs (Figure 4C).^16−22,28−35^ DNA gyrase is an enzyme that converts relaxed double-stranded DNA to negatively supercoiled DNA.^44^ Negative supercoiling provides torsional strain to the DNA strand, which lowers the energy required for DNA double-strand separation.^45^ Biological processes that required for unwinding of the DNA stand, such as DNA replication and transcription, can be affected by the inhibition of negative supercoiling.^46−48^ As shown in Figure 4C, the intensity of the supercoiled DNA band decreased with increasing TPA-1 concentration, completely disappearing at 25 μM of TPA-1. The remaining DNA band was similar to the negative control band when 25 μM TPA-1 was mixed with the same plasmid that was used in the DNA gyrase assay (Figure 4D), suggesting that TPA-1 could inhibit the supercoiling process of *S. aureus* DNA gyrase. These results implied that TPA-1 could effectively eradicate *S. aureus* through multiple antibacterial mechanisms without light irradiation.

### 2.4 Mass Spectrometry (MS)-Based Proteomic Study on TPA-1-Treated *S. aureus* without Light Irradiation

**Figure 5:**
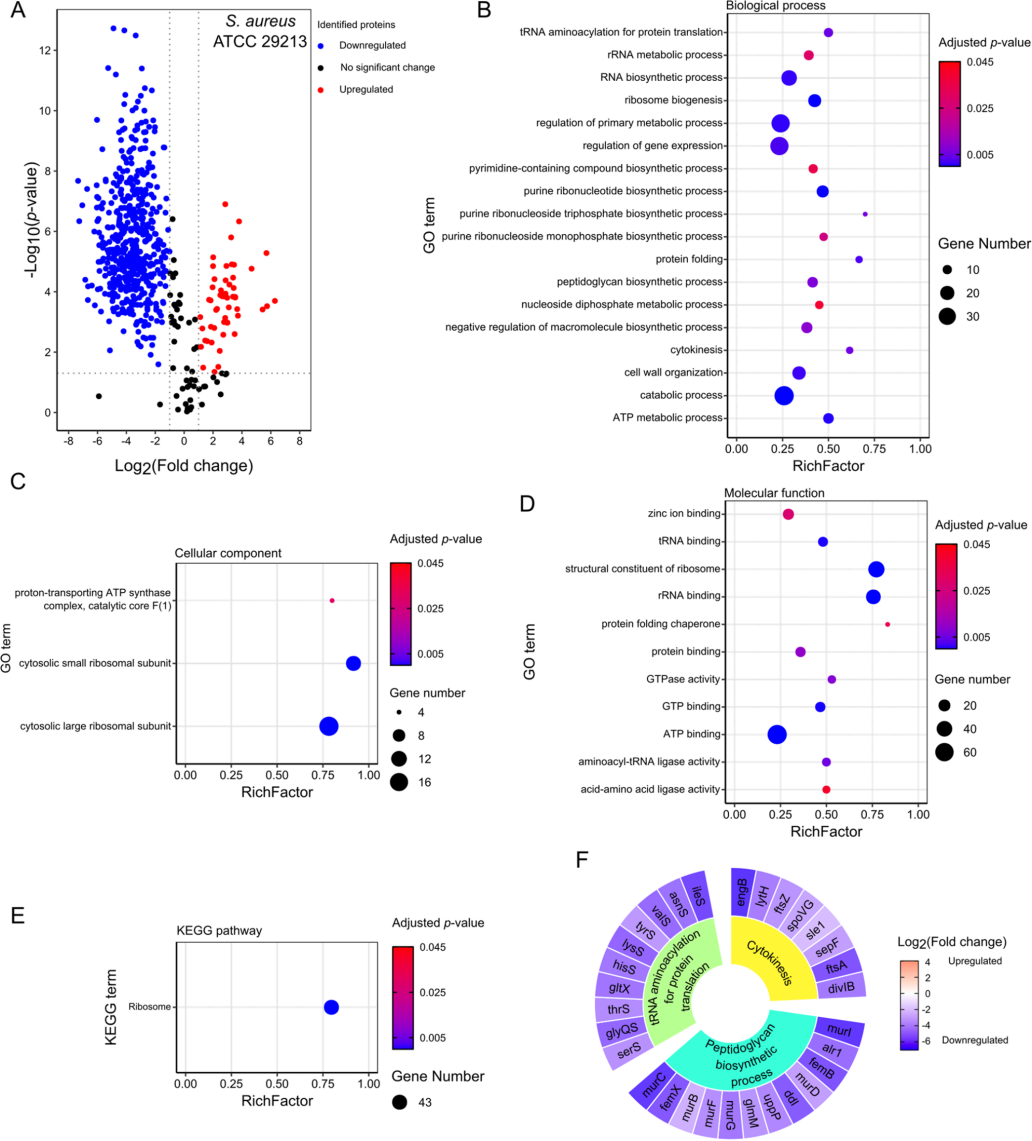
(A) Volcano plot showing differentially expressed proteins (DEPs) in *S. aureus* treated with TPA-1 (2 × MIC, 6.25 μM) *versus* control *S. aureus* after incubation for 1 h at 37 °C in the dark. Gene ontology enrichment analysis in terms of (B) biological function, (C) cellular component, and (D) molecular function and (E) Kyoto Encyclopedia of Genes and Genomes pathway enrichment analysis of the corresponding genes of the DEPs in TPA-1-treated *S. aureus*. (F) Selected biological processes in *S. aureus* affected by treatment with TPA-1. The inner blocks indicate the names of the selected biological processes, while the outer blocks show the fold changes of the corresponding DEPs, which are represented by their gene names. Three independent biological replicates were performed.

Mass spectrometry (MS)-based proteomic analysis was performed to further investigate the intrinsic antibacterial effects of TPA-1 against *S. aureus* without light irradiation (Figure 5). In total, 631 unique protein groups were identified, among which 578 proteins were considered to be differentially expressed proteins (DEPs) based on a *p*-value < 0.05 and |log_2_(fold change)| > 1. Among the 578 DEPs, many were downregulated (524 proteins; Figure 5A). We conducted Gene Ontology (GO) and Kyoto Encyclopedia of Genes and Genomes (KEGG) pathway enrichment analyses on the genes corresponding to the DEPs, using the PANTHER Classification System and clusterProfiler package in RStudio.^49−51^ The GO enrichment analysis categorized the DEPs into biological processes, molecular functions, and cellular components (Figure 5B–D). The KEGG enrichment analysis identified one major KEGG pathway (Figure 5E). Among the GO and KEGG pathway terms, three biological processes that are important for bacterial viability (cytokinesis, the peptidoglycan biosynthetic process, and tRNA aminoacylation for protein translation) were selected for further investigations, and their expression fold changes are presented in Figure 5F.

Cytokinesis and peptidoglycan synthesis are crucial biological processes during bacterial cell division and growth. All of the identified proteins in both processes were downregulated after treatment with TPA-1 at 2 × MIC (6.25 μM) for 1 h in the dark at 37 °C. Related to cytokinesis, the expression levels of FtsZ, FtsA, SepF, and DivlB were decreased. FtsZ is a tubulin-like protein that polymerizes at midcell to form the Z ring during the initiation of bacterial cell division.^52^ The Z ring acts as a platform to recruit other division proteins for divisome assembly and to guide septal cell wall synthesis.^53,54^ FtsA and SepF help the Z ring to anchor to the bacterial membrane, while DivlB accompanies the FtsW/PBP1 complex to induce cell constriction.^55−57^ Treatment with TPA-1 caused the downregulated expression of these proteins, which may interfere with the formation of the divisome and inhibit cell division. In terms of peptidoglycan synthesis, the levels of several proteins related to peptidoglycan precursor synthesis,^58^ such as Mur family proteins (MurB, MurC, MurD, MurF, MurG, and MurI), d-alanine ligase (ddl), and alanine racemase 1 (alr1), were downregulated, indicating that treatment with TPA-1 also affected cell wall synthesis in *S. aureus.* Additionally, treatment with TPA-1 led to the downregulation of ten tRNA-ligases in *S. aureus.* tRNA-ligases are essential for protein translation, as they catalyze the attachment of amino acids to the corresponding tRNA. A decrease in tRNA-ligase expression may result in poor translation efficiency. In summary, the proteomic study of *S. aureus* revealed that TPA-1 without light irradiation affected planktonic cell division, cell wall synthesis, and translation efficiency.

### 2.5 Antiplanktonic Properties of TPA-1 upon Light Irradiation

**Figure 6:**
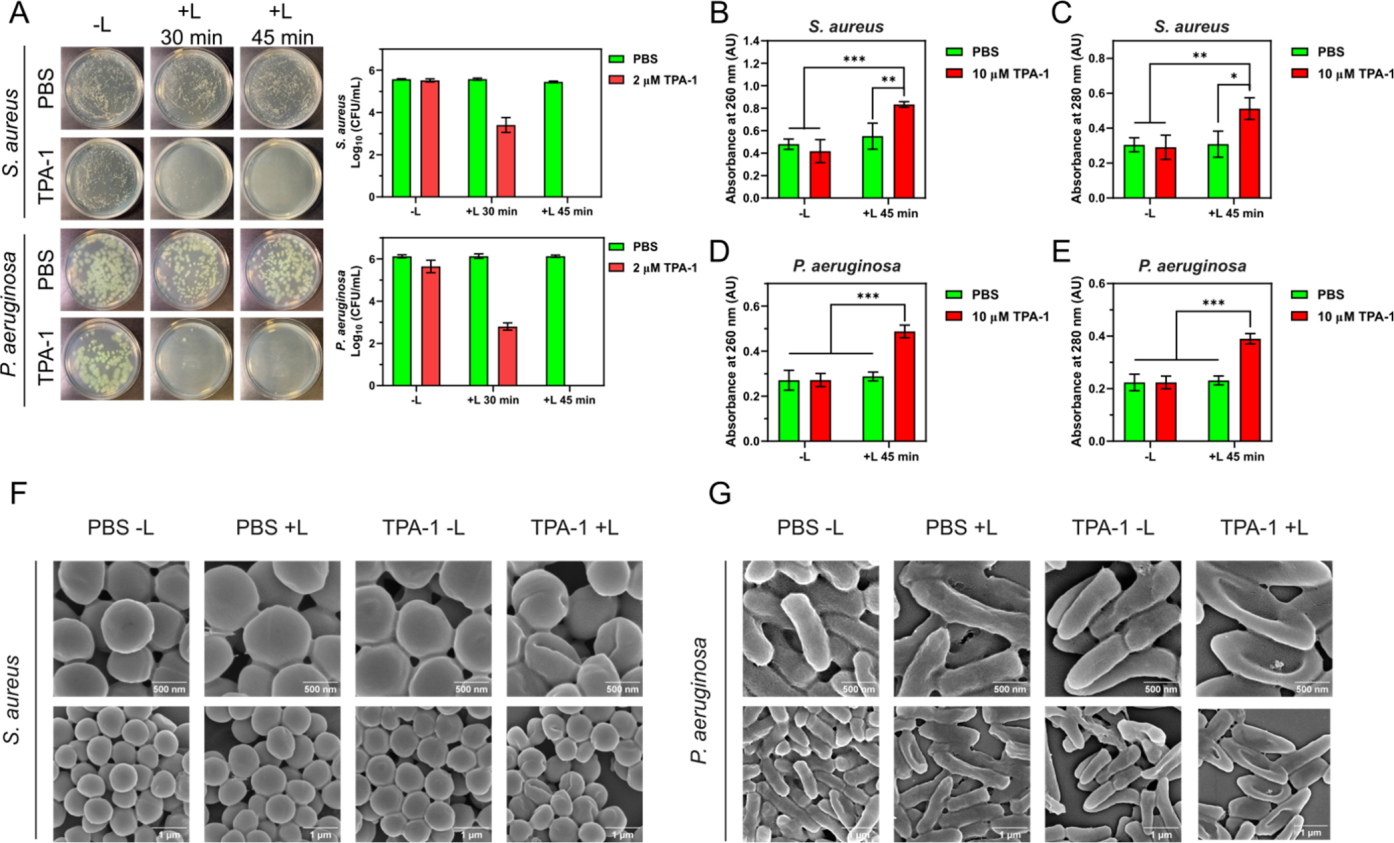
(A) Photographs and numbers of viable planktonic bacteria after PDT. (B) DNA/RNA leakage (measured by absorption at 260 nm) and (C) protein leakage (measured by absorption at 280 nm) of *S. aureus* after PDT. (D) DNA/RNA and (E) protein leakage of *P. aeruginosa* after PDT. Data are presented as the mean ± SD, with *n* = 3 per group. * *p*-Value <0.05, ** *p*-value <0.01, and *** *p*-value <0.001. (F) SEM images of *S. aureus* and (G) *P. aeruginosa* after PDT with TPA-1 (10 μM). −L: without light irradiation. +L: with light irradiation at 600 nm (60 mW/cm^2^) for 45 min.

We next investigated the antibacterial activity of TPA-1 upon light irradiation. The photodynamic eradication abilities of TPA-1 against *S. aureus* and *P. aeruginosa* were assessed after the bacterial cultures were irradiated with light at 600 nm (60 mW/cm^2^). As shown in Figure 6A, at a sub-MIC concentration of 2 μM TPA-1, 99.2% ± 0.7% (2.2 ± 0.4 log CFU/mL reduction) of *S. aureus* and 99.96% ± 0.03% (3.4 ± 0.2 log CFU/mL reduction) of *P. aeruginosa* were eradicated after irradiation for 30 min. Moreover, TPA-1 was capable of eradicating >99.99% (>5 log CFU/mL reduction) of *S. aureus* and *P. aeruginosa* when the irradiation time was increased to 45 min.

### 2.6 Antiplanktonic Mechanism of TPA-1 upon Light Irradiation

Given that the previous BTRC assay indicated that TPA-1 could bind to LTA and LPS, which are located on the respective bacterial membranes of *S. aureus* and *P. aeruginosa*, we hypothesized that the ROS generated by TPA-1 would damage the bacterial membrane. Therefore, we monitored the DNA and protein contents of the supernatants of *S. aureus* and *P. aeruginosa* cultures via absorption at 260 nm (DNA) and 280 nm (protein) before and after the PDT. The absorption of the supernatants of the TPA-1 treatment plus light irradiation groups (TPA-1 + L) for *S. aureus* and *P. aeruginosa* was higher than that of the control groups (Figure 6B–E), indicating that the bacterial membrane was likely damaged and the cellular contents leaked after PDT with TPA-1. Additionally, the SEM images of *S. aureus* and *P. aeruginosa* revealed shrinkages and collapses of the bacterial cells after PDT with TPA-1 (Figure 6F,G). The results indicated that TPA-1 likely eradicated planktonic *S. aureus* and *P. aeruginosa* upon light irradiation by causing bacterial membrane damage.

### 2.7 Antibiofilm Properties of TPA-1 upon Light Irradiation

**Figure 7:**
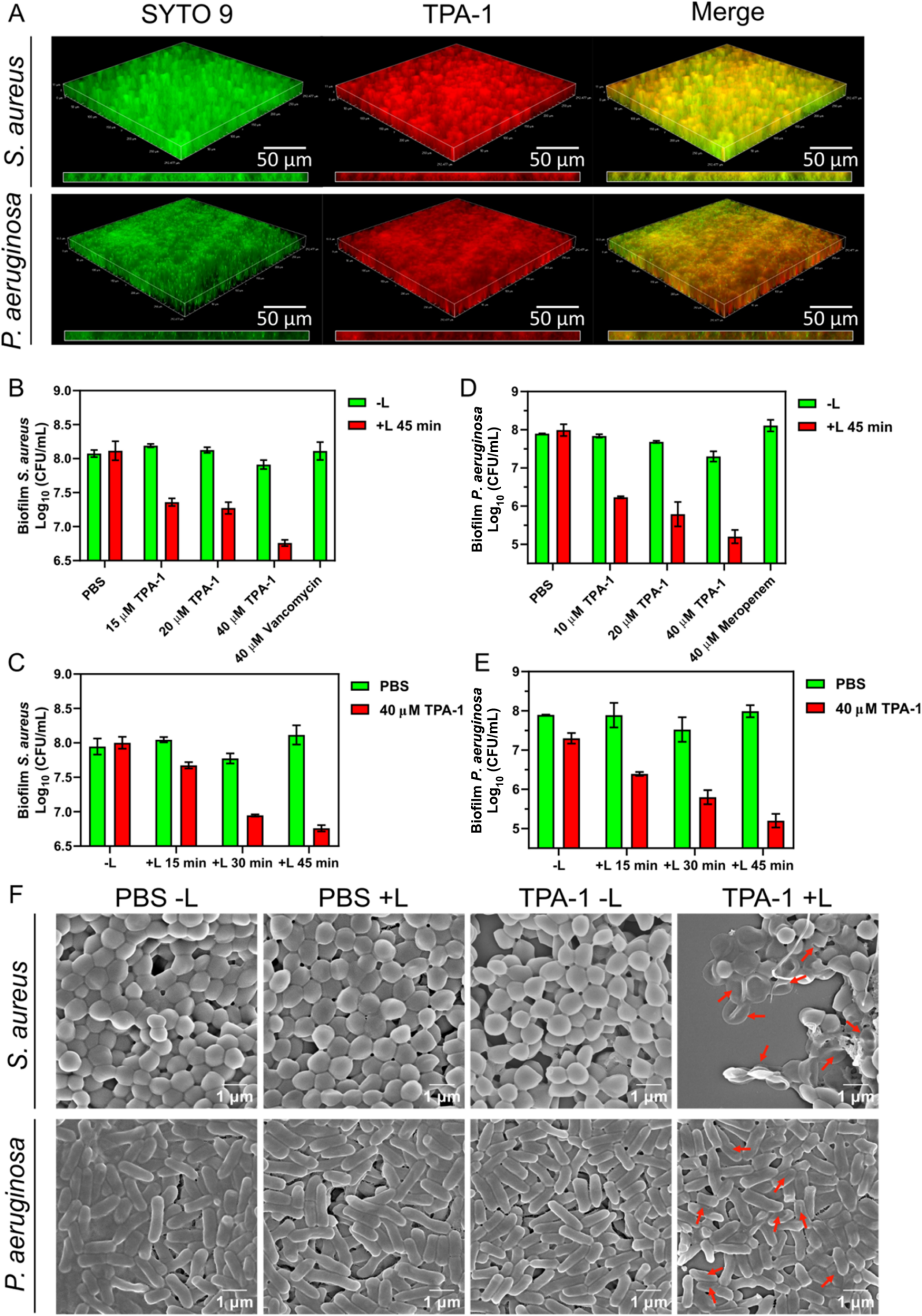
(A) Fluorescence images of the bacterial biofilms treated with SYTO 9 (2.5 μM) and TPA-1 (20 μM). The images inside the rectangular boxes are longitudinal sections of the biofilms. The numbers of viable bacterial cells in the *S. aureus* biofilm after PDT with (B) different TPA-1 concentrations and (C) various irradiation times. The numbers of viable bacterial cells in the *P. aeruginosa* biofilm after PDT with (D) different TPA-1 concentrations and (E) various irradiation times. Data are presented as the mean ± SD, with *n* = 3 per group. (F) SEM images of bacterial biofilms after PDT. The red arrows indicate shrinkage of bacterial cells, suggesting membrane damage. −L: without light irradiation. +L: with light irradiation at 600 nm (60 mW/cm^2^) for 45 min.

In addition to its excellent antiplanktonic ability, TPA-1 exhibited promising antibiofilm abilities against Gram-positive and Gram-negative bacteria upon light irradiation. One of the biggest challenges in eliminating mature biofilms is overcoming the penetration barrier of the EPS produced by bacteria. As shown in Figure 7A, the red fluorescence signal generated by TPA-1 was observed throughout the *S. aureus* and *P. aeruginosa* biofilms, suggesting that TPA-1 could label bacteria inside biofilms. Upon light irradiation at 600 nm for 45 min, TPA-1 was able to eradicate 92% ± 3% (1.18 ± 0.05 log CFU/mL reduction) of *S. aureus* in the biofilm and 99.8% ± 0.1% (2.6 ± 0.2 log CFU/mL reduction) of *P. aeruginosa* in the biofilm, which was more effective than the commercially available antibiotics vancomycin (*S. aureus*) and Meropenem (*P. aeruginosa*) at the same concentration (Figure 7B–E). The photodynamic eradication of biofilm bacteria by TPA-1 was also more potent than that by the reported PS zinc phthalocyanine under the same experimental conditions (Figure S39). These results demonstrated that TPA-1 exhibited promising antibacterial activity against mature biofilms upon light irradiation.

### 2.8 Antibiofilm Mechanism of TPA-1 upon Light Irradiation

The metabolic rate of the biofilms was evaluated using Calcein-AM, a fluorescence dye that generates a green fluorescence signal inside metabolically active cells (Figures S40 and S41). *S. aureus* and *P. aeruginosa* biofilms exhibited lower green fluorescence signals than the control groups after PDT with TPA-1, indicating lower metabolic rates. The decrease in the metabolic rate was likely caused by the eradication of the biofilm bacterial cells during PDT. Moreover, the SEM images of the TPA-1 PDT-treated *S. aureus* and *P. aeruginosa* biofilms revealed defects in their bacterial morphology (Figure 7F). These results implied that TPA-1 could cross the EPS barrier and then generate ROS upon light irradiation, eradicating biofilm bacteria by damaging the bacterial membrane.

MS-based proteomic analysis was also performed on TPA-1 PDT–treated *S. aureus* and *P. aeruginosa* biofilms (Figure S42). In total, 804 unique protein groups were identified in the *S. aureus* biofilm, among which 56 proteins were considered to be DEPs. Among the 56 DEPs, 44 were downregulated and 12 were upregulated (Figure S42A). In total, 1393 unique protein groups were identified in the *P. aeruginosa* biofilm. However, only four were considered DEPs and all were downregulated (Figure S42B). GO and KEGG enrichment analyses were performed on the genes corresponding to the DEPs for the TPA-1 PDT-treated *S. aureus* and *P. aeruginosa* biofilms. Although no significant enrichment was found in the *P. aeruginosa* biofilm, the enrichment analysis of the genes associated with the DEPs in the *S. aureus* biofilm were classified into three GO terms and one KEGG pathway (Figure S42C–F). Based on the annotations, we focused on two terms related to cell growth and virulence: cell wall organization (GO) and *S. aureus* infection (KEGG).

The expression levels of cell-wall-organization-related proteins were decreased in the *S. aureus* biofilm after PDT with TPA-1 (Figure S42G). Teichoic acid glycerol-phosphate primase (tarB) and lipoteichoic acid synthase (ltaS), which are proteins required in the synthesis of wall teichoic acid (WTA) and LTA, respectively, were downregulated.^59,60^ WTA and LTA are important components in *S. aureus* colonization and infection.^61,62^ A decrease in the expression of these proteins may inhibit the synthesis of WTA and LTA, reducing the virulence of the *S. aureus* biofilm. Moreover, TPA-1 upon light irradiation caused the downregulation of proteins related to *S. aureus* infection in the *S. aureus* biofilm. These proteins included γ-hemolysin components B and C (hlgB, hlgC) and staphylococcal superantigen-like 7 (ssl7), which are virulence factors released by *S. aureus* to help evade the host immune system.^63,64^ Downregulations of these proteins could also reduce the virulence of the *S. aureus* biofilm. Overall, the proteomic data hinted that the PDT of TPA-1, apart from eradicating biofilm bacteria, could reduce the virulence of the *S. aureus* biofilm.

### 2.9 Assessments of Potential Off-Target Effects of TPA-1

**Figure 8:**
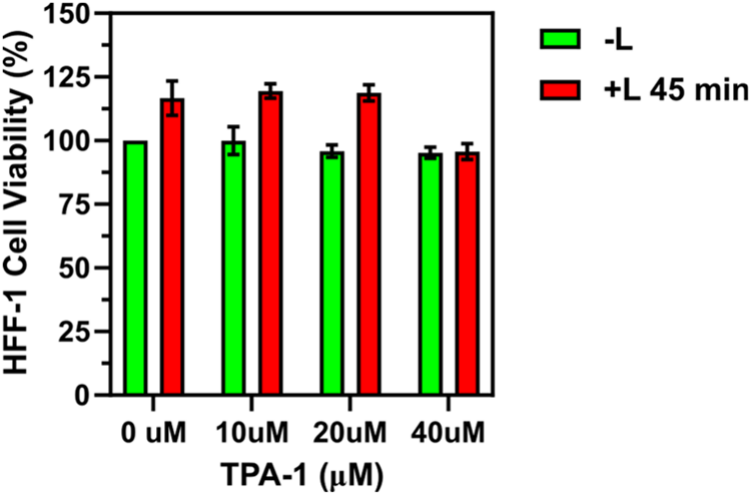
HFF-1 cell viability rate after PDT with different TPA-1 concentrations for 45 min. Data are presented as the means ± SDs, with *n* = 3 per group.

Before *in vivo* studies with TPA-1, selectivity and light cytotoxicity assays were conducted to assess the potential off-target effects of TPA-1. To study the selectivity of TPA-1, we mixed 50,000 HFF-1 cells with 1 × 10^8^ CFU/mL of *S. aureus* or *P. aeruginosa*, followed by incubation with 10 μM TPA-1 (Figure S43). The fluorescence signal from TPA-1 was observed on bacteria but barely found on HFF-1 cells, indicating the high specificity of TPA-1 toward bacteria. In addition, the viability of HFF-1 cells exceeded 90% after incubation with 40 μM TPA-1 and light irradiation for 45 min (Figure 8). Collectively, these results indicated that TPA-1 had high specificity toward bacterial cells and PDT with TPA-1 would not cause severe damage to human cells. Along with the above-mentioned low cytotoxicity and excellent antibacterial abilities of TPA-1, the results suggested that TPA-1 could be safely and effectively adopted in the treatment of *in vivo* bacterial infections.

### 2.10 *In Vivo* Mice Models

**Figure 9:**
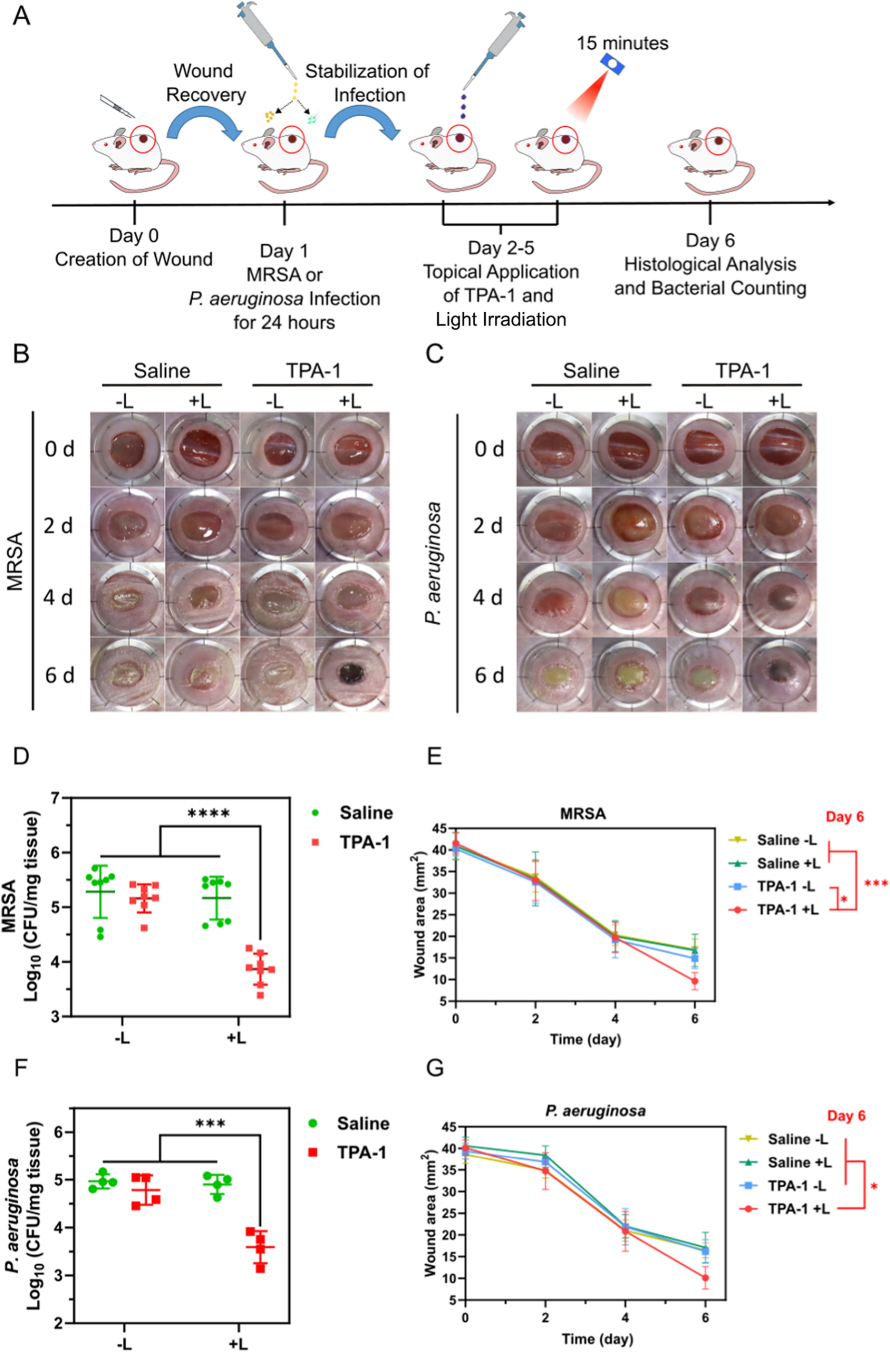
(A) Illustration of *in vivo* experimental procedures. Photographs of (B) MRSA- and (C) *P. aeruginosa*-infected wounds after treatment. (D) Number of viable MRSA and (E) wound areas in infected tissues after various treatments for 4 days. (F) Number of viable *P. aeruginosa* and (G) wound areas in infected tissues after various treatments for 4 days. Data are presented as the mean ± SD, with *n* = 8 per group for the MRSA-infected model and *n* = 4 per group for the *P. aeruginosa*-infected model. * *p*-Value <0.05; *** *p*-value <0.001; **** *p*-value <0.0001. −L: without light irradiation. +L: with light irradiation at 600 nm (60 mW/cm^2^) for 15 min.

Wound infection mouse models with MRSA and *P. aeruginosa* were designed and employed to evaluate the therapeutic efficacy of TPA-1. The general workflow of the *in vivo* experiments is illustrated in Figure 9A. An open wound was created on the back of the mice, and 1 × 10^8^ CFU/mL of MRSA or *P. aeruginosa* was added to the wound after 24 h to avoid inducing severe sepsis.^65^ The wound was covered and left untreated for 24 h to allow bacterial biofilm formation. Subsequently, saline (0.9% NaCl) or TPA-1 (90 μM) was added topically to the wound. After incubation for 10 min, the mice were irradiated with 600 nm light for 15 min or kept in the dark. The treatments were performed every 24 h for 4 consecutive days. The number of bacteria that remained in the wound was counted the day after the last treatment. As shown in Figure 9B,C, 96% ± 3% (1.4 ± 0.6 log (CFU/mg tissue) reduction) of MRSA and 96% ± 4% (1.4 ± 0.4 log (CFU/mg tissue) reduction) of *P. aeruginosa* were eradicated in the infected tissues in the TPA-1 plus light irradiation groups (TPA-1 + L), demonstrating that TPA-1 could eradicate MRSA and *P. aeruginosa* biofilms *in vivo* upon light irradiation. Additionally, the TPA-1 + L groups exhibited the smallest wound areas compared to the other groups after treatment for 4 days (Figure 9D–G). We also conducted histological analysis on MRSA biofilm-infected wounds using hematoxylin and eosin (H&E) staining and Masson’s trichrome staining. As shown in Figure S44, all wounds exhibited signs of inflammation; however, fewer inflammatory cells were infiltrated the wounds and a higher extent of collagen deposition was observed in the wound tissue of the TPA-1 + L group. These results demonstrated that TPA-1 could promote wound healing in bacterially infected wounds upon light irradiation. Altogether, TPA-1 delivered good therapeutic results in treating wounds infected with MRSA and *P. aeruginosa* biofilms.

## 3 Conclusions

We have synthesized a novel water-soluble and bacterial-specific AIE PS that exhibited excellent antibacterial activity with and without light irradiation. Without light irradiation, TPA-1 acts as an effective antibacterial agent, eradicating planktonic *S. aureus* and preventing biofilm formation. The antibacterial mechanism study indicated that TPA-1 depolarizes the *S. aureus* membrane and inhibits the negative DNA supercoiling activity of the *S. aureus* DNA gyrase. The proteomic study further revealed that TPA-1 can affect *S. aureus* cell division, cell wall synthesis, and translation efficiency. Upon light irradiation, TPA-1 acts as an antibacterial PS, eradicating planktonic and biofilm bacteria by generating ROS, which causes bacterial membrane damage. PDT with TPA-1 can also reduce the virulence of the *S. aureus* biofilm. In addition, the therapeutic efficacy of TPA-1 was exemplified in MRSA- and *P. aeruginosa*-infected wounds in mice. This study demonstrates the incorporation of intrinsic antibacterial properties into a PS while providing a good biocompatibility. Given that one of the major flaws of antibacterial PSs is that their antibacterial effect is lost once the light source is removed, we believe that TPA-1 provides a new direction in the development of antibacterial PSs by extending the antibacterial effects even in the dark.

## 4 Experimental Section

### 4.1 Materials and Instruments

4-Bromo-4′,4″-dimethoxytriphenylamine, 1,3-dibromopropane, 4-bromo-1-butyne, bromoethane, 2-methylbenzothiazole, 5-Formyl-2-thienylboronic acid, trimethylamine, and zinc phthalocyanine were purchased from TCI. Boron tribromide and [1,1′-bis(diphenylphosphino)ferrocene]dichloropalladium(II) were purchased from Aladdin. Dulbecco’s Modified Eagle Medium (DMEM), fetal bovine serum (FBS) and penicillin-streptomycin (10,000 U/mL) solution, phosphate-buffered saline (PBS), and trypsin were purchased from Gibco. SYTO 9, hydroxyphenyl fluorescein (HPF), dihydrorhodamine 123 (DHR123), singlet oxygen sensor green (SOSG), Calcein AM, and BODIPY TR-cadaverine were purchased from Invitrogen. 3,3-Dipropylthiadicarbocyanine iodide (Disc3(5)), crystal violet, lysogeny broth (LB) miller, agar, d-(+)-glucose, LTA from *S. aureus* and LPS from *P. aeruginosa* were purchased from Sigma-Aldrich. Mueller–Hinton Broth (MHB) and Tryptic Soy Broth (TSB) were purchased from BD. All chemical reagents and solvents were directly used without further purification. *S. aureus* (ATCC 29213), methicillin-resistant S. aureus (BAA 41 and ATCC 43300), *P. aeruginosa* (ATCC 27853), and human foreskin fibroblast (HFF-1) cells were obtained from American Type Culture Collection (ATCC). MTT assays were performed by using the MTT assay kit from ThermoFisher Scientific. Hemolysis rates of the compounds were assessed by using the hemolysis test kit from Hemoscan.

Ultraviolet–visible(UV–vis) absorption spectra were obtained from an Agilent Technologies Cary 8454 UV–vis spectrometer. Fluorescence spectra were taken on an Agilent Technologies Cary Eclipse Fluorescence Spectrophotometer. TECAN Infinite M1000 PRO was used to perform assays requiring fluorescence intensity measurements. Cytotoxicity, hemolysis, crystal violet staining assays, and cell component leakage assays were conducted on a BMG Labtech CLARIOstar microplate reader. Fluorescence images were taken using a Nikon Eclipse Ti2-E Live-cell Fluorescence Imaging System. SEM images were obtained from a Tescan MAIA3. The photodynamic properties of the compounds were studied using a CEL-HXF300-T3 xenon light system from CEAulight with a 420–780 nm UV–visible cut filter and 600 nm band-pass filter. The *c* log *P* values of the compounds were obtained by using the integrated function in ChemDraw.

### 4.2 Synthesis and Characterization

The synthetic route of all TPA compounds is shown in Scheme S1. Bruker Advance-III 400 MHz Fourier transform nuclear magnetic resonance (FT-NMR) system was used to record the ^1^H and ^13^C NMR spectra of the compounds. The high-resolution mass spectra were acquired on an Agilent 6540 Quadrupole-TOF LC/MS. Melting point experiments were performed on Cole-Parmer MP400. The high-performance liquid chromatography (HPLC) of the compounds were performed on Waters Autopurification HPLC Prep System (2545 Binary Gradient Module with 2767 Sample Manager) with Sunfire Prep C18 OBD 5 μM column (19 mm × 100 mm). ^1^H,^13^C NMR, high-resolution mass spectra, and HPLC spectra are shown in Figures S1–S30. All compounds used for *in vitro* and *in vivo* experiments are >95% pure by HPLC.

#### 4.2.1 Synthesis of Compound **1**

4-Bromo-4′,4″-dimethoxytriphenylamine (0.8 g, 2 mmol) was dissolved in dichloromethane (DCM, 15 mL) and purged with nitrogen. Boron tribromide (6 mmol) was slowly added into the solution at 0 °C. The reaction mixture was then allowed to stir at room temperature overnight. The reaction was quenched with iced methanol (2 mL). After the addition of methanol, some deep blue solids were formed in the solution. DCM (100 mL) was added in several portions to wash the solids. The organic layer was then filtered, washed with water (30 mL, 3 times) and brine (30 mL), and dried with sodium sulfate. The sodium sulfate was removed by filtration, and the filtrate was evaporated under vacuum to obtain crude Compound **1** as a green oil. The crude Compound **1** was directly used to synthesize Compound **2** without further purification.

#### 4.2.2 Synthesis of Compound **2**

Crude Compound **1** (0.7 g, 2 mmol) was dissolved in dimethylformamide (DMF, 5 mL) with 1,3-dibromopropane (7.93 g, 40 mmol). Cesium carbonate (1.99 g, 6.2 mmol) was then added to the solution. The reaction mixture was stirred overnight at room temperature. Upon completion of the reaction, ethyl acetate (80 mL) was added to the solution. The organic solution was washed with water (30 mL, 3 times) and brine (30 mL) and dried with sodium sulfate. The solution was concentrated and purified with column chromatography (hexane/ethyl acetate = 20:1) to obtain Compound **2** as a colorless oil (0.45 g, 38% yield).^1^H NMR (400 MHz, CDCl_3_) δ = 7.29 (d, *J* = 7.92 Hz, 2H), 7.08 (d, *J* = 8.04 Hz, 4H), 6.90–6.85 (m, 6H), 4.12 (t, *J* = 5.28 Hz, 4H), 3.65 (t, *J* = 6.2 Hz, 4H), 2.38–2.32 ppm (m, 4H). ^13^C NMR (100 MHz, CDCl_3_) δ = 155.2, 147.9, 140.8, 131.9, 126.6, 122.2, 115.6, 112.6, 65.6, 32.5, 30.3 ppm. HRMS (ESI) *m*/*z* calcd for C_24_H_24_Br_3_NO_2_^+^: 596.9337 [M]^+^; found: 596.9329.

#### 4.2.3 Synthesis of Compound **3**

Compound **2** (0.45 g, 0.75 mmol) and 5-Formyl-2-thienylboronic acid (0.24 g, 1.5 mmol) were dissolved in degassed methanol/toluene (30 mL, 2:3) with potassium carbonate (0.52 g, 3.75 mmol) and Pd(dppf)Cl_2_ (27 mg, 0.035 mmol). The reaction mixture was purged with nitrogen and heated to reflux overnight. Then, the solvent was evaporated, and the remaining mixture was redissolved in DCM (100 mL). The mixture was washed with water (30 mL, 3 times) and brine (30 mL). The organic layer was dried with sodium sulfate and purified with column chromatography (hexane/ethyl acetate = 10:1) to obtain Compound **3** as a yellow oil (0.2 g, 42% yield). ^1^H NMR (400 MHz, CDCl_3_) δ = 9.82 (s, 1H), 7.67 (d, *J* = 2.76 Hz, 1H), 7.45 (d, *J* = 7.8 Hz, 2H), 7.25 (d, *J* = 0.8 Hz, 1H), 7.08 (d, *J* = 7.72 Hz, 4H), 6.91–6.85 (m, 6H), 4.09 (t, *J* = 5.12 Hz, 4H), 3.61 (t, *J* = 6 Hz, 4H), 2.31 ppm (m, 4H). ^13^C NMR (100 MHz, CDCl_3_) δ = 182.6, 155.6, 155.0, 149.9, 140.8, 140.1, 137.9, 127.2, 127.2, 124.4, 122.4, 119.5, 115.5, 65.6, 32.4, 30.1 ppm. HRMS (ESI) *m*/*z* calcd for C_29_H_27_Br_2_NO_3_S^+^: 629.0058 [M]^+^; found 629.0065.

#### 4.2.4 Synthesis of Compound **4**

Compound **3** (0.2 g, 0.32 mmol) was dissolved in tetrahydrofuran (THF, 5 mL). The solution was then cooled to −78 °C under nitrogen. A large excess of trimethylamine (in THF) was slowly added to the solution. The reaction was stirred at −78 °C for an hour and stirred at room temperature for 3 days. The reaction progress was monitored with a mass spectrometer. The mixture was centrifuged upon completion of the reaction, and the supernatant was discarded. The remaining yellow solid was then washed with THF (40 mL, 4 times) to yield pure Compound **4** (0.18 g, 76% yield) as a yellow solid. ^1^H NMR (400 MHz, MeOD) δ = 9.80 (s, 1H), 7.85, (d, *J* = 2.64 Hz, 1H), 7.54 (d, *J* = 7.88 Hz, 2H), 7.42 (d, *J* = 2.76 Hz, 1H), 7.07 (d, *J* = 8.04 Hz, 4H), 6.95 (d, *J* = 7.88 Hz, 4H), 6.85 (d, *J* = 7.88 Hz, 2H), 4.12 (t, *J* = 5.12 Hz, 4H), 3.62 (t, *J* = 7.92 Hz, 4H), 3.22 (s, 18H), 2.35–2.28 ppm (m, 4H). ^13^C NMR (100 MHz, MeOD) δ = 184.6, 157.0, 156.3, 151.4, 142.1, 141.8, 140.4, 128.5, 128.3, 125.8, 124.1, 120.5, 116.8, 66.1, 65.6, 53.7, 24.4 ppm. HRMS (ESI) *m*/*z* calcd for C_35_H_45_N_3_O_3_S^2+^ 293.6586 [M-2Br]^2+^; found 293.6594.

#### 4.2.5 Synthesis of M1

2-Methylbenzothiazole (0.5 g, 3.36 mmol) and 4-bromo-1-butyne (2.23 g,16.78 mmol) were added into a pressure tube with 2 mL of acetonitrile. The reaction mixture was heated to 110 °C overnight. After the reaction mixture cooled to room temperature, diethyl ether (10 mL) was added to the mixture. The precipitate was filtered and redissolved in water (10 mL). The solution was then washed with DCM until no starting materials were observed on the TLC plate. The aqueous layer was then concentrated, and THF was added for precipitation. The solid was filtered and washed with THF to obtain M1 (0.24 g, 25% yield) as a white solid. ^1^H NMR (400 MHz, MeOD) δ = 8.35–8.31 (m, 2H), 7.95–7.91 (m, 1H), 7.86–7.82 (m, 1H), 5.03 (t, *J* = 6.56 Hz, 2H), 3.34 (s, 3H), 3.06–3.02 (m, 2H), 2.51 ppm (t, *J* = 2.64 Hz, 1H). ^13^C NMR (100 MHz, MeOD) δ = 179.4, 142.4, 131.1, 130.6, 129.9, 125.5, 118.1, 79.5, 74.3, 19.1, 17.8 ppm. HRMS (ESI) *m*/*z* calcd for C_12_H_12_NS^+^ 202.0685 [M-Br]^+^; found 202.0692.

#### 4.2.6 Synthesis of M2

M2 was synthesized similarly to M1 while 4-bromo-1-butyne was replaced with bromoethane. M2 was obtained as a white solid (0.3 g, 35% yield). ^1^H NMR (400 MHz, DMSO-*d*_6_) δ = 8.47 (d, *J* = 8.12 Hz, 1H), 8.35 (d, *J* = 8.4 Hz, 1H), 7.89 (t, *J* = 7.48 Hz, 1H), 7.80 (t, *J* = 7.64 Hz, 1H), 4.80–4.75 (m, 2H), 3.22 (s, 3H), 1.45 (t, *J* = 7.08 Hz, 3H). ^13^C NMR (100 MHz, MeOD) δ = 177.8, 142.3, 131.1, 130.9, 129.8, 125.4, 117.8, 46.4, 13.7. HRMS (ESI) *m*/*z* calcd for C_10_H_12_NS^+^ 178.0685 [M-Br]^+^; found 178.0692.

#### 4.2.7 Synthesis of TPA-1

Compound **4** (0.1 g, 0.13 mmol), M1 (73 mg, 0.26 mmol), and a catalytic amount of potassium carbonate (9 mg) were dissolved in absolute ethanol (15 mL). The reaction was heated to reflux under nitrogen for 24 h. The reaction was then concentrated, filtered, and purified by preparative HPLC using a C18 column with acetonitrile (0.1% TFA) and water (0.1% TFA) as the gradient mobile phase. The eluent was concentrated and freeze-dried to obtain TPA-1 as a dark purple solid (40 mg, 30% yield). Melting point range: 77–81 °C. ^1^H NMR (400 MHz, MeOD) δ = 8.37 (d, *J* = 15.12 Hz, 1H), 8.21 (d, *J* = 8.08 Hz, 1H), 8.16 (d, *J* = 8.48, 1H), 7.84 (t, *J* = 7.56 Hz, 1H), 7.77–7.74 (m, 2H), 7.61–7.58 (m, 3H), 7.47 (d, *J* = 4.04, 1H), 7.11 (d, *J* = 8.84, 4H), 6.97 (d, *J* = 8.88 Hz, 4H), 6.87 (d, *J* = 8.76, 2H), 5.06 (t, *J* = 6.12 Hz, 2H), 4.12 (t, *J* = 5.56 Hz, 4H), 3.62–3.58 (m, 4H), 3.20 (s, 18H), 3.01–2.98 (m, 2H), 2.48 (t, *J* = 2.4 Hz, 1H), 2.34–2.28 ppm (m, 4H). ^13^C NMR (100 MHz, MeOD) δ = 173.7, 157.2, 156.0, 151.6,143.2, 142.6, 141.6, 139.6, 138.5, 130.8, 129.6, 129.0, 128.6, 128.3, 125.2, 124.9, 120.3, 117.3, 116.7, 110.5, 79.7, 74.4, 66.0, 65.5, 53.7, 53.6, 53.6, 24.4, 19.4 ppm. HRMS (ESI) *m*/*z* calcd for C_47_H_55_N_4_O_2_S_2_^3+^ 257.1250 [M-3Br]^3+^; found 257.1263. HPLC purity: 98.38%.

#### 4.2.8 Synthesis of TPA-0

TPA-0 was synthesized like TPA-1 while replacing M1 with M2. TPA-0 was obtained as a dark purple solid (50 mg, 39% yield). Melting point range:130–135 °C.^1^H NMR (400 MHz, MeOD) δ = 8.35 (d, *J* = 15.12, 1H), 8.19 (d, *J* = 8.04, 1H), 8.13 (d, *J* = 8.52, 1H), 7.84 (t, *J* = 7.52, 1H), 7.77–7.72 (m 2H), 7.58 (d, *J* = 8.76 Hz, 2H), 7.47–7.42 (m, 2H), 7.11 (d, *J* = 8.88 Hz, 4H), 6.97 (d, *J* = 8.92, 4H), 6.85 (d, *J* = 8.8, 2H), 4.93–4.84 (m, 2H), 4.13 (t, *J* = 5.56 Hz, 4H), 3.62–3.58 (m, 4H), 3.21 (s, 18H), 2.35–2.28 (m, 4H), 1.58 ppm (t, *J* = 7.2 Hz, 3H). ^13^C NMR (100 MHz, MeOD) δ = 172.2, 157.2, 155.8, 151.6, 143.6, 142.5, 141.6, 139.5, 139.1, 138.3, 130.9, 129.5, 129.4, 128.6, 125.6, 125.1, 124.9, 120.2, 109.4, 66.0, 65.5, 53.7, 53.6, 53.6, 24.4, 14.2 ppm. HRMS (ESI) *m*/*z* calcd for C_45_H_55_N_4_O_2_S_2_^3+^ 249.1250 [M-3Br]^3+^; found 249.1262. HPLC purity: 98.59%.

#### 4.2.9 Synthesis of Compound **5**

4-Bromo-4′,4″-dimethoxytriphenylamine (0.25 g, 0.65 mmol), 5-formyl-2-thienylboronic acid (0.2 g, 1.3 mmol), Pd(dppf)Cl_2_ (24 mg, 0.033 mmol), and potassium carbonate (0.45 g, 3.25 mmol) were added into a mixture of degassed methanol/toluene (1:1). The mixture was refluxed under nitrogen overnight. The solvent was then evaporated, and the solid was redissolved in DCM (100 mL). The mixture was washed with water (30 mL, 3 times), brine (30 mL), and dried with sodium sulfate. Then, the mixture was concentrated and purified by column chromatography (hexane/ethyl acetate = 5:1) to get Compound **5** (190 mg, 70% yield) as an orange solid. ^1^H NMR (400 MHz, CDCl_3_) δ = 9.83 (s, 1H), 7.68 (d, *J* = 2.84 Hz, 1H), 7.46, (d, *J* = 7.68, 2H), 7.25 (d, *J* = 7.92, 1H), 7.09 (d, *J* = 7.72, 4H), 6.91–6.85 (m, 6H), 3.81 ppm (s, 6H). ^13^C NMR (100 MHz, CDCl_3_) δ = 182.6, 156.5, 155.1, 150.0, 140.8, 139.9, 137.9, 127.2, 127.2, 124.2, 122.3, 119.3, 114.9, 55.5 ppm. HRMS (ESI) *m*/*z* calcd for C_25_H_21_NO_3_S^+^ 415.1242 [M]^+^; found 415.1245.

#### 4.2.10 Synthesis of TPA-NC-1

Compound **5**, M2, and a catalytic amount of potassium carbonate (9 mg) were dissolved in absolute ethanol. The reaction was purged with nitrogen and refluxed for 24 h. The mixture was concentrated and purified by column chromatography (DCM/methanol = 10:1) to give TPA-NC-1 as a black solid (20 mg, 13% yield). Melting point range: 161–165 °C. ^1^H NMR (400 MHz, DMSO-*d*_6_) δ = 8.44–8.38 (m, 2H), 8.23 (d, *J* = 8.48 Hz, 1H), 7.95 (d, *J* = 3.12 Hz, 1H), 7.83 (t, *J* = 7.56 Hz, 1H), 7.74 (t, *J* = 7.6 Hz, 1H), 7.61–7.55 (m, 4H), 7.12 (d, *J* = 8.28 Hz, 4H), 6.97 (d, *J* = 7.72 Hz, 4H), 6.76 (d, *J* = 8.16 Hz, 2H), 4.91–4.86 (m, 2H), 3.76 (s, 6H), 1.44 ppm (t, *J* = 6.84 Hz, 3H). ^13^C NMR (100 MHz, MeOD) δ = 172.3, 158.5, 156.2, 151.9, 143.7, 142.5, 141.0, 139.7, 138.2, 130.8, 129.5, 129.4, 128.7, 128.2, 125.2, 125.0, 124.9, 119.9, 117.0, 116.1, 109.2, 56.0, 45.4, 14.2 ppm. HRMS (ESI) *m*/*z* calcd for C_35_H_31_N_2_O_2_S_2_^+^ 575.1822 [M-Br]^+^; found 575.1839. HPLC purity: 95.30%.

#### 4.2.11 Log* P* of TPA Compounds

The log *P* values of TPA compounds were obtained according to previously reported methods.^66^ 1-octanol was first presaturated with water. Equal volume and concentration of TPA-NC-1, TPA-0, and TPA-1 were added to a 1-octanol/water (1:1) mixture and vortexed for 1 min. The mixture was then centrifuged for 5 min at 13,500 rpm. After that, the absorptions of the compound in the 1-octanol and water phases were measured. The concentrations of the compound in both phases were calculated by using the absorption calibration curve of the corresponding compound. Log *P* = log (concentration of the compound in 1-octanol/concentration of the compound in water).

#### 4.2.12 Stability of TPA-1 in pH 6.5 -pH 8.5

50 μM of TPA-1 solutions (50 μM) were prepared in 0.1 mM Tris–HCl buffers at different pHs (pH 6.5, 7.0, 7.5, 8.0, and 8.5). Then, the solutions were incubated in the dark at 37 °C. Every 30 min, a portion of the TPA-1 solution was taken out, and the concentration of the solution was recorded by measuring the absorption at 550 nm. This experiment was performed in triplicate.

#### 4.2.13 LTA and LPS-Binding Assays

One mg/mL of LTA and LPS stock solutions were prepared in water. 500 μM of BODIPY TR-cadaverine stock solution was prepared in DMSO. In a 96-well plate, BODIPY TR-cadaverine (final working concentration = 5 μM) was first mixed with LTA (final working concentration = 5 μg/mL) or LPS (final working concentration = 10 μg/mL) in Tris buffer (50 mM, pH 7.4) and incubated in the dark for 15 min at room temperature. Then, compounds (final working concentration of 10 μM) were added to the well. The mixture was further incubated for 30 min before the fluorescence intensities were recorded (excitation 580 nm, emission 620 nm) with a plate reader (TECAN Infinite M1000 PRO). The displacement percentage was calculated as (*F*_compound_ – *F*_buffer_)/(*F*_probeonly_ – *F*_buffer_) × 100% where *F*_compound_ is the fluorescence intensity after the addition of compound, *F*_buffer_ is the fluorescence intensity after the addition of buffer as a negative control, and *F*_probe only_ is the fluorescence intensity of BODIPY TR-cadaverine alone in buffer without LPS or LTA as positive control. The experiments were at least triplicated.^40,41^

#### 4.2.14 Cytotoxicity Assay

The cytotoxicity of TPA-1, TPA-0, and TPA-NC-1 against HFF-1 cells was evaluated using the 3-(4,5-dimethylthiazol-2-yl)-2,5-diphenyltetrazolium bromide (MTT) assay kit from ThermoFisher Scientific. In general, HFF-1 cells (MEM, 10% FBS, and 1% penicillin-streptomycin) were seeded in a 96-well plate with a density of 3000 cells/well overnight at 37 °C with 5% CO_2_. Then, the cells were treated with different concentrations of compounds for 24 h. MTT (10 μL, 5 mg/mL) was added to the medium, and the cells were further incubated for 3 h. The medium was then replaced by DMSO (100 μL). After gently shaking the plate for 30 s, the optical density of the solutions at 570 nm was measured by a BMG Labtech CLARIOstar microplate reader. The cell viability was calculated as (OD_compound_ – OD_DMSO_)/(OD_medium_ - OD_DMSO_) × 100% where OD_compound_, OD_medium_, and OD_DMSO_ are the optical densities of the treated cell, nontreated cells, and DMSO at 570 nm, respectively. The experiment was conducted in triplicate. The CC_50_ values were calculated using GraphPad Prism 8.

#### 4.2.15 Hemolysis

The human erythrocyte concentrate was prepared according to the kit manual from the Biomaterial Hemolytic Assay Kit (Hemoscan). After the human erythrocytes had been washed and diluted with manufacture buffer, 100 μL of erythrocytes (10%) were added into 100 μL of various concentrations of compounds in PBS. 1% of Triton X (1%) was the positive control, while PBS was the negative control. The erythrocytes were then gently mixed with the TPA compounds and further incubated for an hour at 37 °C. After that, the erythrocytes were centrifuged (1000 rpm, 5 min), and 20 μL of supernatant was added to 180 μL of PBS in a 96-well plate. The optical density at 380, 415, and 450 nm was measured by a plate reader. The final OD value of the compounds was calculated as (2 × OD_415_) – (OD_450_ + OD_380_) where OD_380_, OD_415_, and OD_450_ are the optical density of the solution at 380, 415, and 450 nm, respectively. The hemolysis rates of the compounds were calculated as (OD_compound_ – OD_PBS_)/(OD_TritonX_ – OD_PBS_) × 100% where OD_compound_, OD_PBS_, and OD_TritonX_ are the final OD values of the erythrocytes mixed with TPA compounds, PBS, and 1% Triton X, respectively. The experiment was performed in triplicate.

#### 4.2.16 Fluorescence Imaging of HFF-1 Cells

HFF-1 cells were seeded in confocal dishes at a cell density of 50,000 cells overnight at 37 °C. TPA compounds (10 μM) were added to the medium and incubated with the cells for 20 min. The fluorescence images of the cells were then taken using a Nikon Eclipse Ti2-E Live-cell Fluorescence Imaging System with an excitation wavelength of 550 nm. Emission wavelengths were collected from 590 to 670 nm for the TPA compounds.

#### 4.2.17 ROS Generation Assays

Commercial ROS detection probes (DHR123, HPF, and SOSG) were used to evaluate the ROS generation abilities of TPA compounds. DHR123, HPF, and SOSG were used to detect nonspecific ROS, hydroxyl radical, and singlet state oxygen, respectively. In general, DHR123 (5 μM), HPF (5 μM), or SOSG (2.5 μM) was mixed with 10 μM TPA compounds in PBS. TPA-1 and TPA-0 were irradiated by 600 nm light (60 mW/cm^2^) for 15 min. The change in the fluorescence intensity of the mixture was recorded by a plate reader. The fluorescence intensity for DHR123 was recorded at 529 nm with an excitation wavelength of 507 nm. The fluorescence intensity for HPF was recorded at 515 nm with an excitation wavelength of 490 nm. The fluorescence intensity for SOSG was recorded at 525 nm with an excitation wavelength of 504 nm. The experiment was performed in triplicate.

#### 4.2.18 Fluorescence Imaging of Bacterial Cells

Overnight culture of *S. aureus* (ATCC 29213) or *P. aeruginosa* (ATCC 27853) in Tryptic Soy Broth (TSB) was diluted 100 times in fresh TSB and grown to OD = 1. The bacterial culture was diluted to 2 × 10^8^ CFU/mL with PBS. TPA compounds (20 μM) were mixed with the bacteria and incubated for 20 min at 30 °C. SYTO 9 (2.5 μM) was also used to stain the bacterial DNA. The bacteria (1 μL) were then immobilized on a 1.2% agarose pad. The fluorescence images of the bacteria were captured from the Nikon Eclipse Ti2-E Live-cell Fluorescence Imaging System with excitation wavelengths of 490 (SYTO 9) and 550 nm (TPA-1). Emission wavelengths were collected from 500 to 550 nm for SYTO9 and 590 to 670 nm for TPA-1.

#### 4.2.19 Minimum Inhibition Concentration (MIC) Assay

The MIC assays of TPA-1 were conducted according to the broth microdilution methods described in the Clinical and Laboratory Standards Institute (CLSI) standard. A single colony of *S. aureus* (ATCC 29213), methicillin-resistant *S. aureus* (BAA 41), or *P. aeruginosa* (ATCC 27853) on an LB agar plate was picked and suspended in Mueller–Hinton Broth (MHB). The bacterial culture was grown overnight at 37 °C. The next day, the bacteria culture was diluted 100 times in fresh MHB and grown to the mid log phase. Bacteria in the mid log phase were further diluted to 5 × 10^6^ CFU/mL in cation-adjusted MHB (*S. aureus*) or MHB (*P. aeruginosa*). Ten μL of the diluted bacteria were added to a 96-well plate containing 90 μL of serial diluted TPA-1 in the corresponding medium. The plate was incubated overnight at 37 °C. The MICs of the TPA compounds were the concentrations at which no apparent bacteria were observed by the naked eye. Different concentrations of LTA were also added to examine the change in MIC values of TPA-1 against *S. aureus*. The experiments were conducted in triplicate.

#### 4.2.20 *S. aureus* Biofilm Inhibition and Eradication Assay in the Dark

For biofilm inhibition assay, overnight culture of *S. aureus* in TSB was diluted to 1 × 10^7^ CFU/mL in TSB (with 1% glucose). Ten μL of the diluted bacteria were added to a 96-well plate containing 90 μL of serial diluted TPA-1 in TSB (with 1% glucose). After overnight incubation at 37 °C in darkness, the medium in the well was removed. The remaining biofilm was washed with 200 μL of PBS (3 times) and fixed with 100 μL of methanol for 10 min. The well was left for drying. Then, 150 μL of 0.1% crystal violet was added to the wells and stained for 10 min. The crystal violet was removed, and the wells were washed with 200 μL of PBS (3 times). After drying, the remaining crystal violet was dissolved in 100 μL of 95% ethanol with gentle shaking, and the absorbance at 570 nm was recorded. The biofilm inhibition % was calculated as [1 – (OD_TPA-1_ – OD_blank_)/(OD_medium_ – OD_blank_)] × 100% where OD_TPA-1_, OD_medium_ and OD_blank_ were the absorbances of the wells with bacteria treated TPA-1, with bacteria without treatment and with medium only, respectively. The experiment was performed in triplicate.

For biofilm eradication assay, overnight culture of *S. aureus* in TSB under darkness was diluted to 1 × 10^5^ CFU/mL in TSB (with 1% glucose). 100 μL of the diluted culture was transferred to each well of a 96-well plate. The plate was kept at 37 °C for 24 h under darkness to form mature biofilms. The medium was removed, and the biofilms were washed with 200 μL of PBS (3 times). 100 μL of the premixed TPA-1 solutions in TSB (with 1% glucose) were added to the wells. The plate was further incubated for 24 h under darkness and subjected to the crystal violet assay as stated in the biofilm inhibition assay. The biofilm survival rate (%) was calculated as (OD_TPA-1_ – OD_blank_)/(OD_medium_ – OD_blank_) × 100% where OD_TPA-1_, OD_medium_, and OD_blank_ were the absorbances of the wells with bacteria treated TPA-1, with bacteria without treatment and with medium only, respectively. The experiment was performed in triplicate.

#### 4.2.21 Time-Kill Kinetic against *S. aureus*

Similar to the MIC assay, the overnight culture of *S. aureus* incubated under darkness was diluted in fresh MHB and grown to the mid log phase. The *S. aureus* was then diluted to 1 × 10^6^ CFU/mL in cation-adjusted MHB. Three mL of diluted *S. aureus* was transferred into a 15 mL culture tube. 2 × MIC and 4 × MIC of TPA-1 was added into the bacterial culture and incubated under darkness for 24 h. 2 × MIC of vancomycin and PBS were taken as the positive and negative controls, respectively. At time 0, 0.5, 1, 2, 4, 6, 8, and 24 h, 20 μL of the bacteria culture was taken out. The bacteria were serially diluted, and the number of viable bacteria was recorded using the plate counting method. The experiment was performed in triplicate.

#### 4.2.22 Resistance Development Assay on *S. aureus*

The MICs for TPA-1 and norfloxacin against *S. aureus* were first determined by the above-mentioned method. The wells with 0.5 × MIC of compounds were transferred to 1 mL fresh MHB and incubated under darkness for 3–4 h at 37 °C. The bacteria were then used to conduct a MIC assay again. The experiments were repeated for 20 days. The fold changes in the MIC values of the compounds were recorded. The experiments were performed in duplicate.

#### 4.2.23 Bacterial Membrane Depolarization Assay on *S. aureus*

This assay is conducted according to the previous publication with minor adjustments.^67^ Disc3(5) was used to evaluate the membrane depolarization ability of the TPA compounds. The overnight culture of *S. aureus* was diluted 100 times in fresh MHB and grown to the mid log phase. The bacterial culture was centrifuged (3900*g*, 5 min) and washed with glucose-supplemented HEPES buffer (5 mM glucose, 5 mM HEPES, pH 7.4). The cell platelet was resuspended in the glucose-supplemented HEPES buffer with an addition of 100 mM potassium chloride and diluted to 1 × 10^8^ CFU/mL. 150 μL of the diluted bacteria were transferred to each well of a 96-well plate. Disc3(5) (8 μM, 50 μL) was mixed with the cells and incubated under darkness for 30 min. Before adding TPA compounds, the background fluorescence was measured for 2 min at 670 nm with an excitation wavelength of 622 nm. Then, 10 μL of TPA compounds were added to the wells. The fluorescence intensity at 670 was further recorded for 24 min. The experiments were performed in triplicate.

#### 4.2.24 Scanning Electron Microscope (SEM) Imaging of Bacterial Cells

For studying the intrinsic antibacterial abilities of TPA-1, *S. aureus* was prepared to the mid log phase according to the method described in the MIC assay. The *S. aureus* was then diluted to 1 × 10^8^ CFU/mL in cation-adjusted MHB. 4 × MIC, and 8 × MIC of TPA-1 was added into the bacterial culture and incubated for 3 h at 37 °C in darkness. The cells were centrifuged (3900*g*, 5 min) and washed with PBS. 2.5% Glutaraldehyde was used to fix the bacteria at 4 °C overnight. The fixed bacteria were then gradually dehydrated with 30, 50, 70, 90%, and absolute ethanol. The samples were allowed to stand for 5 min in each dehydration step. After dehydration, the samples were resuspended in 10 μL of absolute ethanol and dropped on silicon slides. The samples were air-dried and imaged by SEM (Tescan MAIA3).

For studying the photodynamic eradication abilities of TPA-1 against planktonic bacteria, overnight culture of *S. aureus* or *P. aeruginosa* in TSB was diluted 100 times in fresh TSB and regrown to OD = 1. The bacterial culture was diluted to 1 × 10^8^ CFU/mL in PBS. TPA-1 (10 μM) was added to the cells and incubated for 20 min at 30 °C in darkness. Then, the bacteria were irradiated by 600 nm light (60 mW/cm^2^) for 45 min. After irradiation, the sample was prepared for SEM imaging, as described above.

For studying the photodynamic eradication abilities of TPA-1 against biofilm bacteria, overnight cultures of *S. aureus* or *P. aeruginosa* were diluted to 1 × 10^5^ CFU/mL in TSB (with 1% glucose). For the *S. aureus* biofilm, 100 μL of the diluted culture was transferred to a 96-well plate. For the *P. aeruginosa* biofilm, 200 μL of the diluted culture was transferred to an 8-well chamber. The plate or the chamber was incubated for 24 h at 37 °C in darkness. After incubation, the planktonic bacterial suspension was removed, and the well was washed with PBS (3 times). TPA-1 (10 μM of *P. aeruginosa*, 40 μM for *S. aureus*) in PBS was added into the wells and incubated for 20 min at 30 °C in darkness. The wells were kept in the dark or irradiated by 600 nm light (60 mW/cm^2^) for 45 min. For the *S. aureus* biofilm, the biofilm was mixed with 2.5% glutaraldehyde overnight at 4 °C. Then, the *S. aureus* biofilm was resuspended and centrifuged (3900*g*, 5 min). The supernatant was discarded. The remaining biofilm was dehydrated and dropped on a silicon slide for SEM. For *P. aeruginosa* biofilm, the biofilm was first resuspended and centrifuged (3900*g*, 5 min) to remove the TPA-1 solution. 2.5% Glutaraldehyde was then added to the biofilm for overnight fixing at 4 °C. The fixed *P. aeruginosa* biofilm was dehydrated and dropped on a clean silicon slide as described above.

#### 4.2.25 DNA-Binding Assay

The genomic DNA of *S. aureus* was extracted using a DNA extraction kit from Sigma-Aldrich. The extracted DNA was diluted to 10 μg/mL and mixed with different concentrations of TPA-1 or 5 μM of SYTOX Green. The mixtures were mixed with loading dye and loaded on a 0.8% agarose gel with DNA stain for electrophoresis (120 V, 40 min). The gel image was then observed and captured using the Bio-Rad ChemiDoc imaging system.

#### 4.2.26 *S. aureus* DNA Gyrase Inhibition Assay

The assay was conducted using an *S. aureus* gyrase supercoiling assay kit from Inspiralis. In general, relaxed bacterial plasmid (pBR322), *S. aureus* gyrase, and different concentrations of TPA-1 were mixed and incubated at 37 °C for 30 min. The mixtures were then quenched and subjected to electrophoresis (1% agarose gel, 75 V, 2 h). The gel image was captured with the Bio-Rad ChemiDoc imaging system.

#### 4.2.27 Photodynamic Eradication Assays

For planktonic bacteria, an overnight culture of *S. aureus* or *P. aeruginosa* was diluted 100 times in TSB and grown to OD = 1. The bacteria cultures were then diluted to 5 × 1 × 10^6^ CFU/mL in PBS. TPA-1 (2 μM) was added to the diluted cultures and incubated under darkness for 20 min at 30 °C. Bacterial cultures of 200 μL were transferred to the wells of a 96-well plate. The plate was irradiated by 600 nm light (60 mW/cm^2^) for 30 and 45 min. Then, the bacterial cultures were serially diluted, and the number of viable bacteria was counted using the plate counting method.

For biofilm bacteria, overnight culture of *S. aureus* or *P. aeruginosa* was diluted to 1 × 10^5^ CFU/mL in TSB (with 1% glucose). Portion of 100 μL of the diluted bacteria culture was added to the wells of a 96-well plate. The plate was incubated under darkness for 24 h at 37 °C. Then, the planktonic bacterial suspensions were removed from the wells. The biofilms were washed with PBS (3 times) to remove any remaining planktonic bacteria. Different concentrations of TPA-1 were dissolved in PBS. TPA-1 solution was added to the biofilms (120 μL for *S.aureus*, 150 μL for *P.aeruginosa*). After 20 min of incubation under darkness at 30 °C, the biofilms were kept in the dark or irradiated by 600 nm light (60 mW/cm^2^) for 15, 30, and 45 min. After that, the TPA-1 solution was replaced by 120 μL of PBS. The biofilm was then suspended in PBS by scratching, followed by sonication (5 min, ≤42 kHz) and vortex (5 min, 950 rpm). The number of viable bacteria was determined by the plate counting method. All experiments were carried out in triplicate.

#### 4.2.28 DNA and Protein Leakage Assays

Mid log phase *S. aureus* and *P. aeruginosa* in TSB were prepared as described above. The bacteria were washed and resuspended in PBS to a final OD = 1. TPA-1 (10 μM) was added to the bacteria and incubated for 20 min at 30 °C in darkness. The bacteria were then irradiated by 600 nm light for 45 min. After treatments, the bacteria were centrifuged (3900*g*, 5 min), and the supernatant was transferred to a UV-transparent 96-well plate. The absorption at 260 nm (DNA) and 280 nm (protein) were recorded by a plate reader. The experiment was performed in triplicate.

#### 4.2.29 Change in Metabolic Rate of Bacterial Biofilm

Calcein-AM was used to study the metabolic rate of the treated bacterial biofilm.^68^ The mature *S. aureus* and *P. aeruginosa* biofilms were prepared as described above. The biofilms were treated with TPA-1 (10 μM) with or without light irradiation. After treatments, the biofilms were further incubated for 1 h at 37 °C in darkness. Then, the TPA-1 solution was removed, and 2 μM Calcein-AM in PBS was added to the biofilms. The biofilms were incubated for another hour at 37 °C in darkness. The Calcein-AM was removed and replaced with PBS. After 15 min of incubation at room temperature in darkness, the green fluorescence signal of Calcein-AM was captured by the Nikon Eclipse Ti2-E Live-cell Fluorescence Imaging System with an excitation wavelength of 490 nm. Emission wavelengths were collected from 500 to 550 nm. The experiments were duplicated.

#### 4.2.30 Mass Spectrometry-Based Proteomic Study

For planktonic analysis, an overnight culture of *S. aureus* was diluted 100 times in CaMHB and grown to OD = 0.8–1. The *S. aureus* was then diluted to 1 × 10^8^ CFU/mL in CaMHB. The diluted bacteria culture with or without 2 × MIC of TPA-1 (6.25 μM) was incubated for 1 h at 37 °C. Bacteria culture with no addition of TPA-1 was used as control. After incubation, the bacteria were collected by centrifugation (3900*g*, 10 min). The proteomic samples were prepared from the bacterial cell pallet using EasyPep MS Sample Prep Kits from Thermo Scientific. For biofilm analysis, the bacterial biofilms were prepared using the same methods used in photodynamic eradication assays. The bacterial biofilms were treated with 40 μM of TPA-1 or PBS and incubated in the dark for 20 min at 30 °C. The biofilms were then irradiated by 600 nm light (60 mW/cm^2^) for 45 min or kept in the dark. After PDT, the TPA-1 solution or PBS was removed. After that, the biofilm proteomic samples were prepared by using EasyPep MS Sample Prep Kits.

The tryptic digests (2 μL) were injected into a Dionex UltiMate 3000 RSLCnano (Thermo Scientific). A trap-and-elute method was adopted with a PepMap 7 cm × 75 μm C18 (Thermo Scientific) and an Aurora 25 cm × 75 μm C18 column with CSI emitter (IonOpticks, Australia) with a trapping flow of 50 μL/min for 2 min in 50 °C. The elution gradient was applied with water (Solvent A) and acetonitrile (Solvent B) in 0.1% formic acid as follows: 2% B in 0–2 min, 6–30% B in 2–79 min, 30–90% B in 79–82 min, 90% B in 82–87 min, and 2% B from 87 to 89 min, in a constant 300 nL/min flow rate. Eluted samples were then analyzed by an Orbitrap Fusion Lumos Mass Spectrometer (Thermo Scientific) using data-independent acquisition (DIA) in the positive ion mode. The source parameters were 2600 V of capillary voltage with a capillary temperature of 300 °C. Full-scan MS spectra were acquired from 400 to 1500 *m*/*z*, with a resolution of 60,000 and an automatic gain control (AGC) target of 400,000. MS/MS was acquired using an Orbitrap as mass analyzer with a mass resolution of 15,000 and standard AGC target. Data analysis was done using Spectronaut (Version 19, Biognosys). Default directDIA+ workflow was used to search against the reviewed *S. aureus* (11,274 sequences; 8 December 2024) and *P. aeruginosa* (3966 sequences, 31 October 2024) database from Uniprot. Peptides search for Trypsin/P cleavages with a maximum allowance of 2 missed cleavages. Fixed modifications of carbamidomethyl on cysteine residues and variable modifications of oxidation on methylation residues are included. 0.01 False Discovery Rate (FDR) was applied for peptides identification.

Differentially expressed protein was defined as *p*-value <0.05 and |log_2_(fold change)| > 1. GO enrichment analysis was conducted using PANTHER Overrepresentation Test (Released 20240807) with Fisher’s Exact as significant test and Bonferroni correction for multiple testing. Corrected *p*-value <0.05 considered as significant. *S. aureus* (all genes in the database) and *P. aeruginosa* (all genes in the database) were used as the reference list for *S. aureus* and *P. aeruginosa*, respectively. KEGG pathway enrichment analysis was conducted in RStudio using clusterProfiler package. The analysis was performed with adjusted *p*-value cutoff = 0.05 and Bonferroni as the *p*-value-adjusted method. The differentially expressed proteins were searched against KEGG organism sao and pae for *S. aureus* and *P. aeruginosa*, respectively.

#### 4.2.31 Selective Labeling of Bacteria over HFF-1 Cells

HFF-1 cells were seeded and cultured in confocal dishes at a density of 50,000 cells for 12 h at 37 °C in darkness. The cells were washed with PBS (3 times) to remove the antibiotics from the medium. TPA-1 (10 μM) was first mixed with 1× 10^8^ CFU/mL *S. aureus* or *P. aeruginosa* in PBS. The mixture was then added to the cells and incubated for 20 min at 30 °C in darkness. The fluorescence signal from TPA-1 was observed using a Nikon Eclipse Ti2-E Live-cell Fluorescence Imaging System with an excitation wavelength of 550 nm. Emission wavelengths were collected from 590 to 670 nm for TPA-1.

#### 4.2.32 Light Toxicity on HFF-1 Cells

HFF-1 cells were seeded in a 96-well plate with a density of 10,000 cells/well for 12 h at 37 °C in darkness. Different concentrations of TPA-1 were added to the cell culture medium and incubated for 20 min at 37 °C in darkness. The cells were then incubated in the dark or irradiated by 600 nm light for 45 min. The cell viability was then evaluated using the MTT assay, which was described in the cytotoxicity assay.

#### 4.2.33 *In Vivo* Model

All *in vivo* experiments were approved by the Department of Science and Technology of Zhejiang Province (SYXK(Zhejiang)2021-0043). Female BALB/c mice (10–12 weeks old) were kept in 12 h light and 12 h dark cycle with access to free food and water for 7 days before and throughout the experiment. The backs of the mice were shaved and disinfected with iodine. A full-thickness circular wound (7 mm in diameter) was then created at the back of the mice. The mice were allowed to recover for 24 h after surgery to prevent sepsis.^65^ Ten μL of 1× 10^8^ CFU/mL MRSA (ATCC 43300) or *P. aeruginosa* (ATCC 27853) in saline was added to the wound. The wound was covered with an adhesive dressing for 24 h of infection. Next, the mice were randomly divided into four groups: (1) 50 μL TPA-1 (90 μM) with 600 nm light irradiation (60 mW/cm^2^) for 15 min, (2) 50 μL TPA-1 (90 μM) in dark, (3) 50 μL saline with 600 nm light irradiation (60 mW/cm^2^) for 15 min, and (4) 50 μL saline in dark. For light irradiation groups, TPA-1 and saline were incubated on the wound for 10 min before irradiation. The treatment was sustained for 4 days. The wound size and body weight of the mice were recorded every day. On day 6, the mice were euthanatized, and the wound tissue was collected. After that, the wound tissue was cut in half, and half of it was used in hematoxylin and eosin (H&E). The other half of the tissue was homogenized, and the number of viable bacteria was measured by the plate counting method.

#### 4.2.34 General Statistical Analysis

Experimental data were presented as the means ± standard deviations (SD). Analysis was conducted using GraphPad Prism 8. Group comparisons were performed using one- or two-way ANOVA followed by a Tukey posthoc test. A probability (*p*) value less than 0.05 was taken as statistical significance (*p* < 0.05).

## Data Availability

The mass spectrometry proteomics raw data have been deposited to in the ProteomeXchange Consortium *via* the PRIDE^69^ partner repository, under data set identifier PXD060538.

## Supporting Information Available

^1^H and ^13^C NMR spectra; high-resolution mass spectra; HPLC traces; absorption and fluorescence spectra; additional *in vitro* and *in vivo* assays results (PDF)
List of identified proteins for proteomic analysis (XLSX)
Molecular formula strings (CSV)

## Abbreviations Used

*c* Log *P*: calculated log value of partition coefficient
Log *P*: log value of partition coefficient
TLC: thin layer chromatography
calcd: calculated
