# Supplementary material for: A Water-Soluble Aggregation-Induced Emission Photosensitizer with Intrinsic Antibacterial Activity as an Antiplanktonic and Antibiofilm Therapeutic Agent
Source: J Med Chem. 2025 Apr 5;68(8):8768–85. doi: 10.1021/acs.jmedchem.5c00403 (PMC12035805; doi:10.1021/acs.jmedchem.5c00403)
Supplement: Supplementary file 1 — jm5c00403_si_001.pdf [file jm5c00403_si_001.pdf]

Supporting Information

A Water-Soluble Aggregation-Induced Emission Photosensitizer with Intrinsic Antibacterial Activity as an Antiplanktonic and Antibiofilm Therapeutic Agent

Cheung-Hin Hung[1], Ka Hin Chan[1], Wai-Po Kong[1], Ruo-Lan Du[1], Kang Ding[1], Zhiguang Liang[1], Yong Wang[1]*, and Kwok-Yin Wong[1]*

[1] State Key Laboratory of Chemical Biology and Drug Discovery, Department of Applied Biology and Chemical Technology, The Hong Kong Polytechnic University, Kowloon, Hong Kong, China.

* Corresponding authors:

Kwok-Yin Wong, E-mail: kwok-yin.wong@polyu.edu.hk*

Yong Wang, E-mail: yong.wang.abct@connect.polyu.hk*

Table of Contents

Supporting Results and Discussion S3

1. Synthetic Route of TPA-1, TPA-0 and TPA-NC-1 .... S3
2. Characterization of TPA-1, TPA-0, and TPA-NC-1 .... S4
3. HPLC Purity Analysis on TPA-NC-1, TPA-0, and TPA-1 .... S17
4. Absorption Spectra of TPA Compounds and Fluorescence spectra of TPA-1 .... S18
5. ROS Generation Assays of TPA-1 and TPA-0 without Light Irradiation S18
6. Stability of TPA-1 in pH 6.5-8.5 .... S19
7. Antibacterial Abilities of TPA-1 without Light Irradiation S19
8. Antibiofilm Abilities of TPA-1 with Light Irradiation S24
9. Selectivity Assays of TPA-1 .... S27
10. *In vivo* Assays S27

Supporting Results and Discussion

1. Synthetic Route of TPA-1, TPA-0 and TPA-NC-1

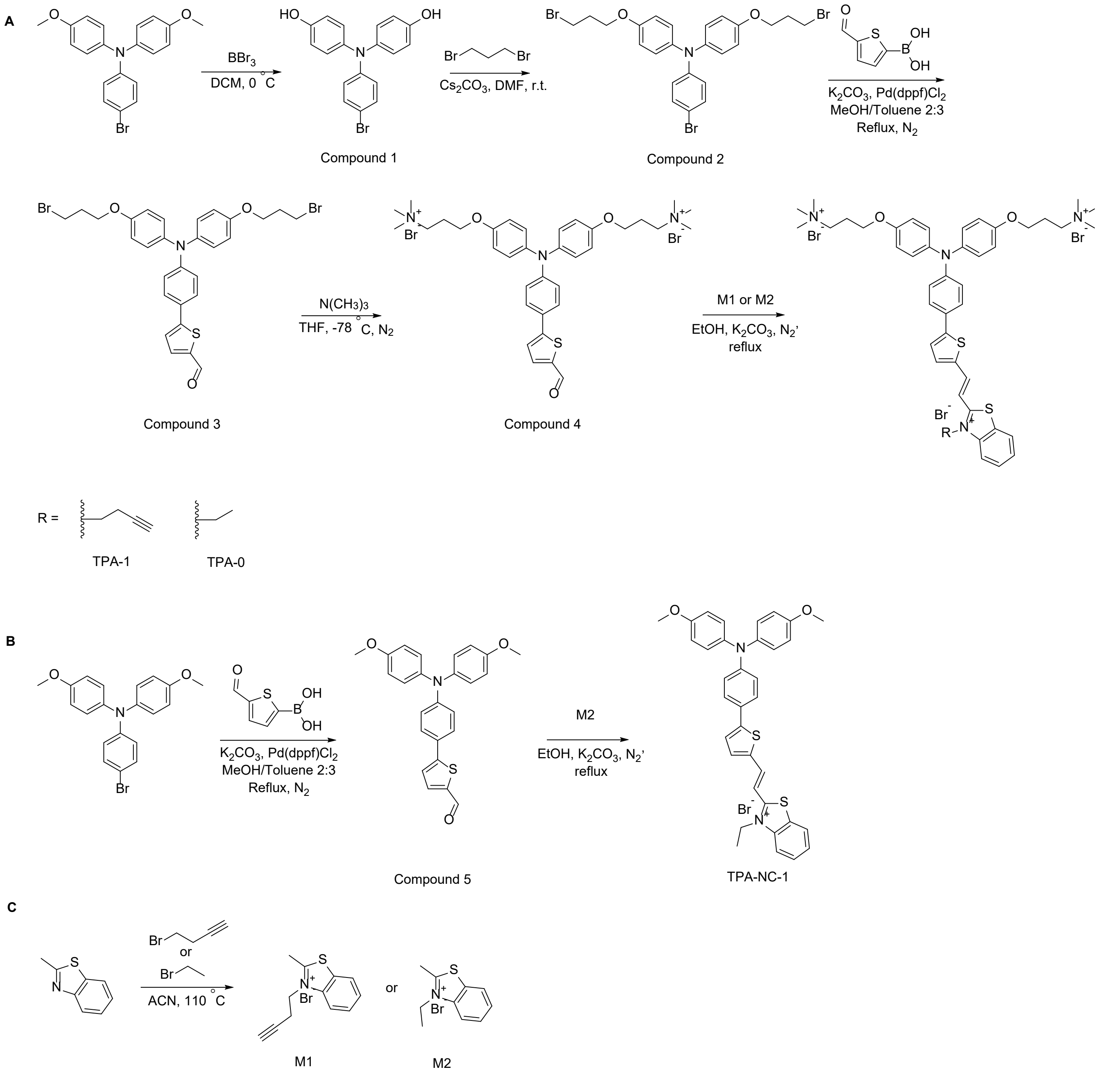

Scheme S1. Synthetic route to (A) TPA-1 or TPA-0, (B) TPA-NC-1 and (C) M1 or M2.

2. Characterization of TPA-1, TPA-0, and TPA-NC-1

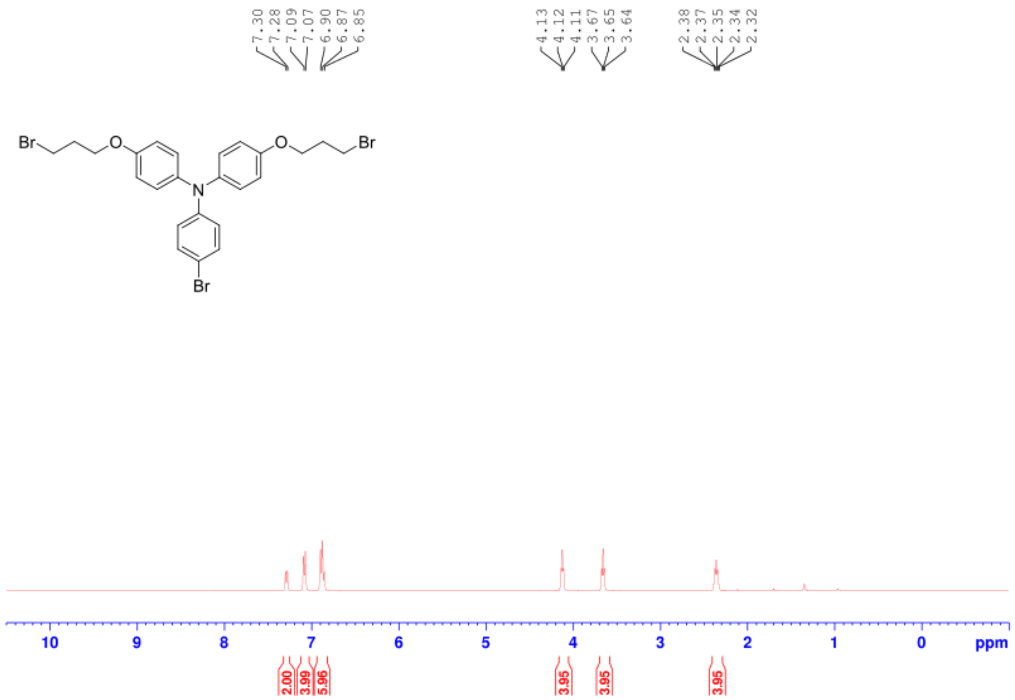

Figure S1. ^{1}H NMR spectrum (400 MHz, $CDCl_3$) of compound 2.

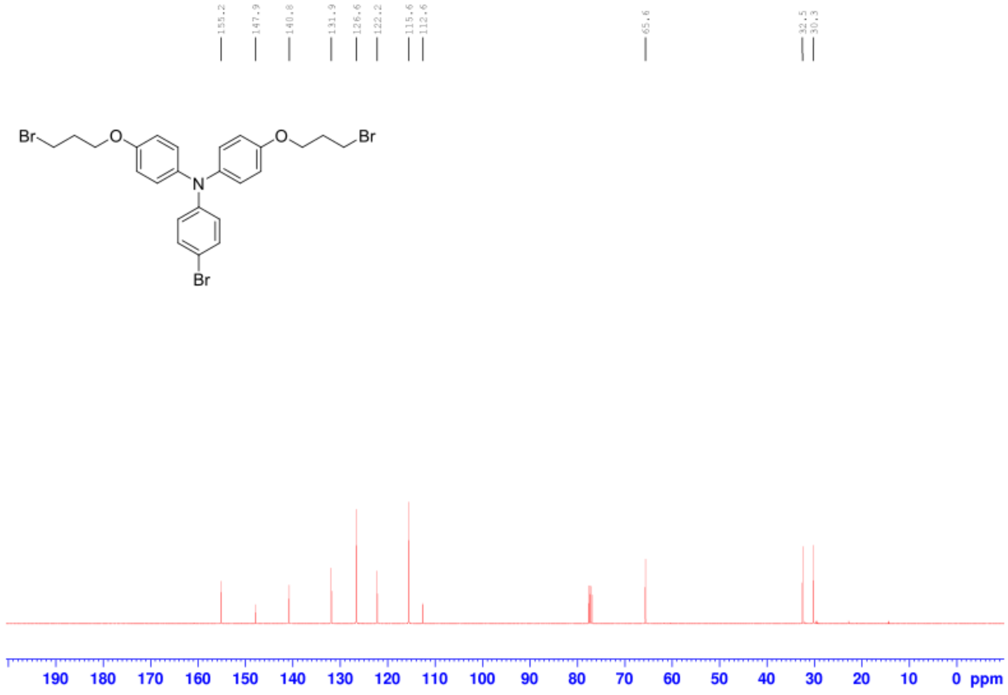

Figure S2. ^{13}C NMR spectrum (100 MHz, $CDCl_3$) of compound 2

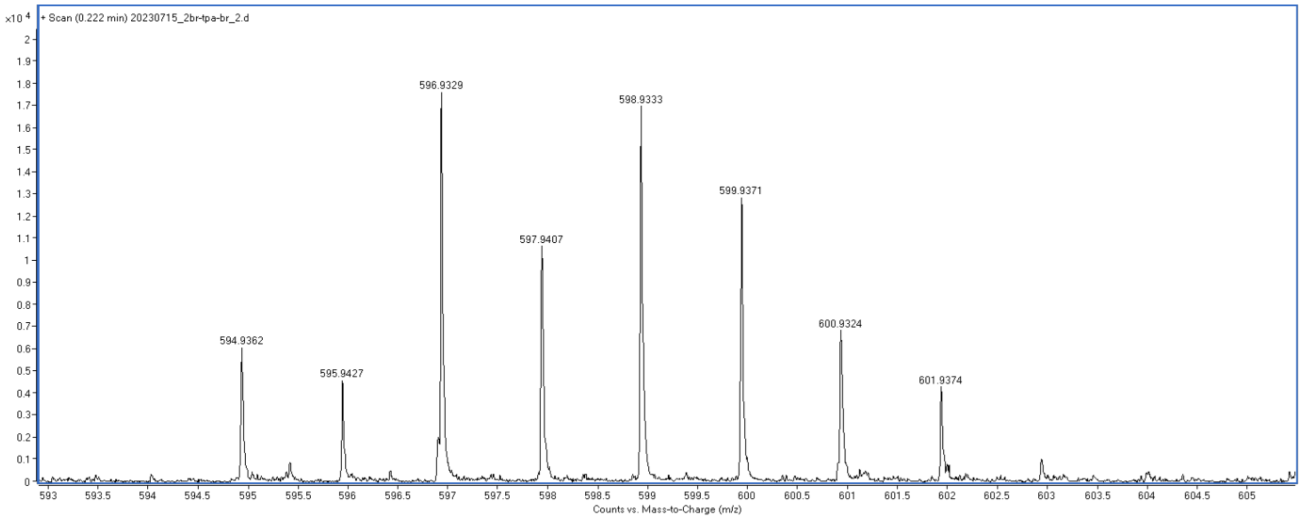

Figure S3. High resolution mass spectrum (ESI) of compound 2

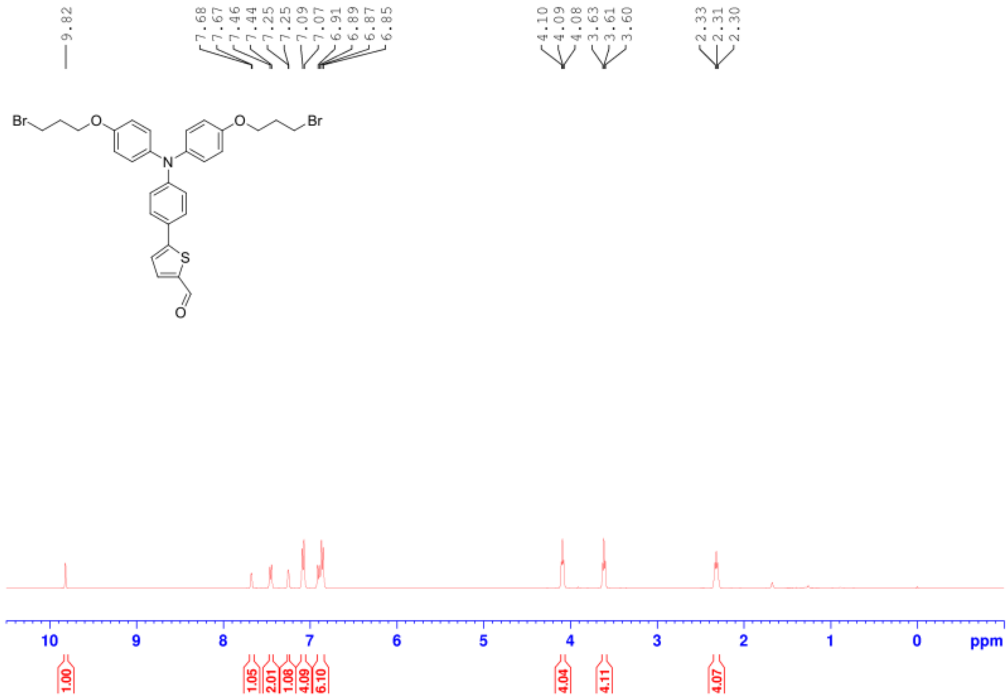

Figure S4. ^{1}H NMR spectrum (400 MHz, $CDCl_3$) of compound 3.

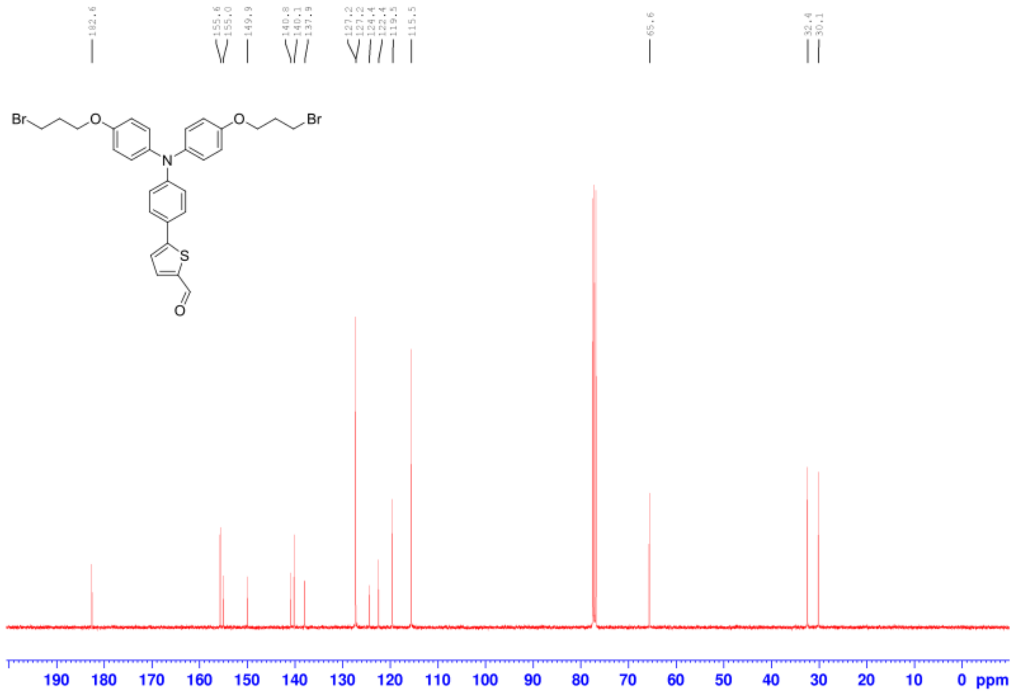

Figure S5. ^{13}C NMR spectrum (100 MHz, $CDCl_3$) of compound 3.

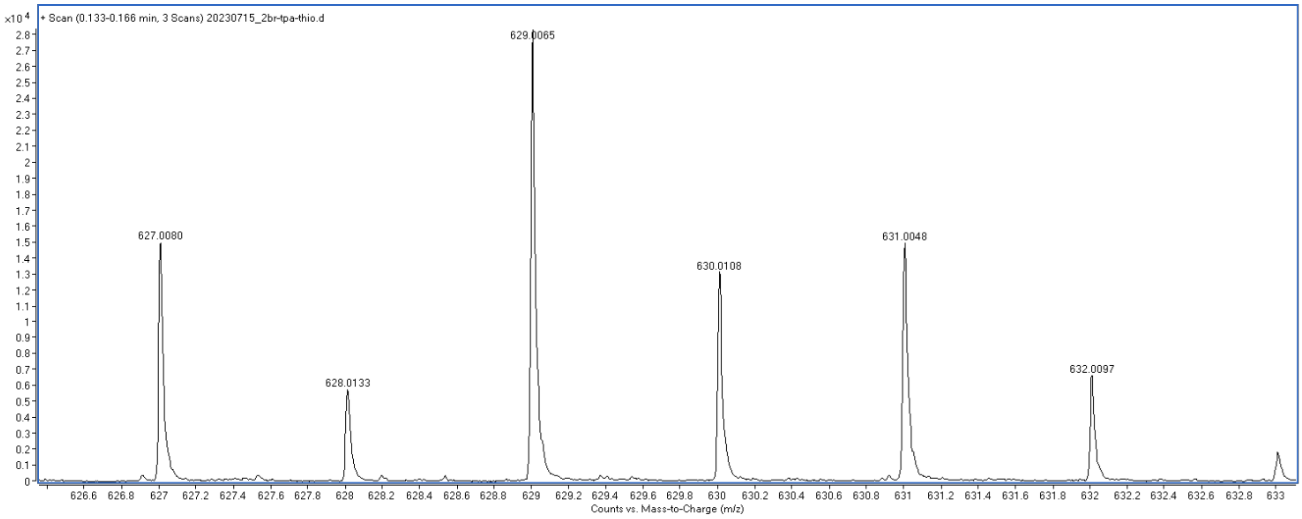

Figure S6. High resolution mass spectrum (ESI) of compound 3.

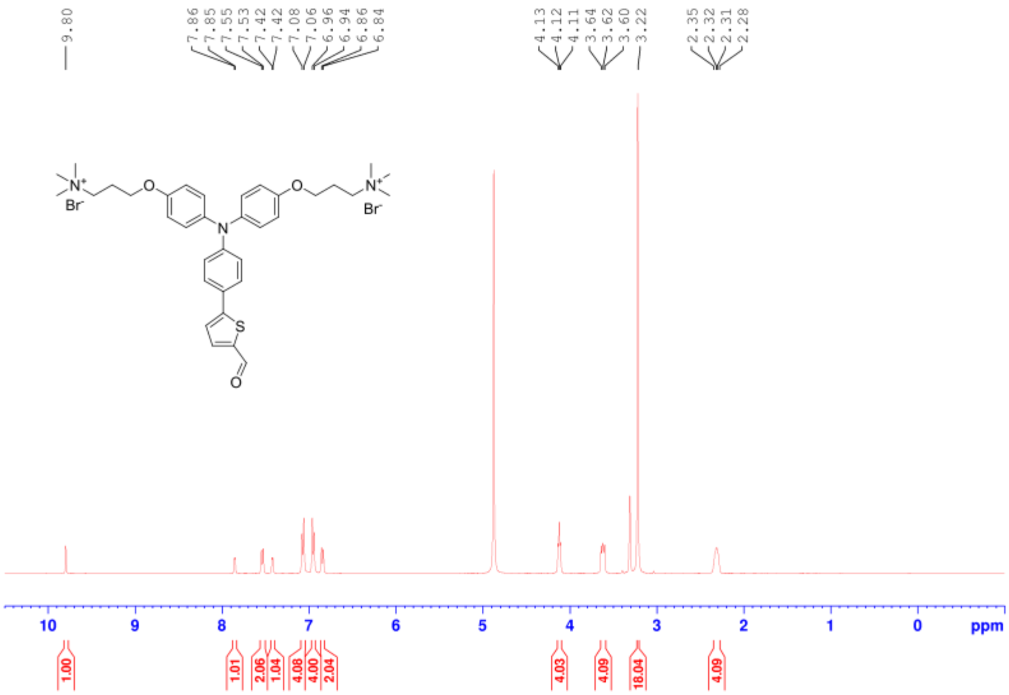

Figure S7. ^{1}H NMR spectrum (400 MHz, MeOD) of compound 4.

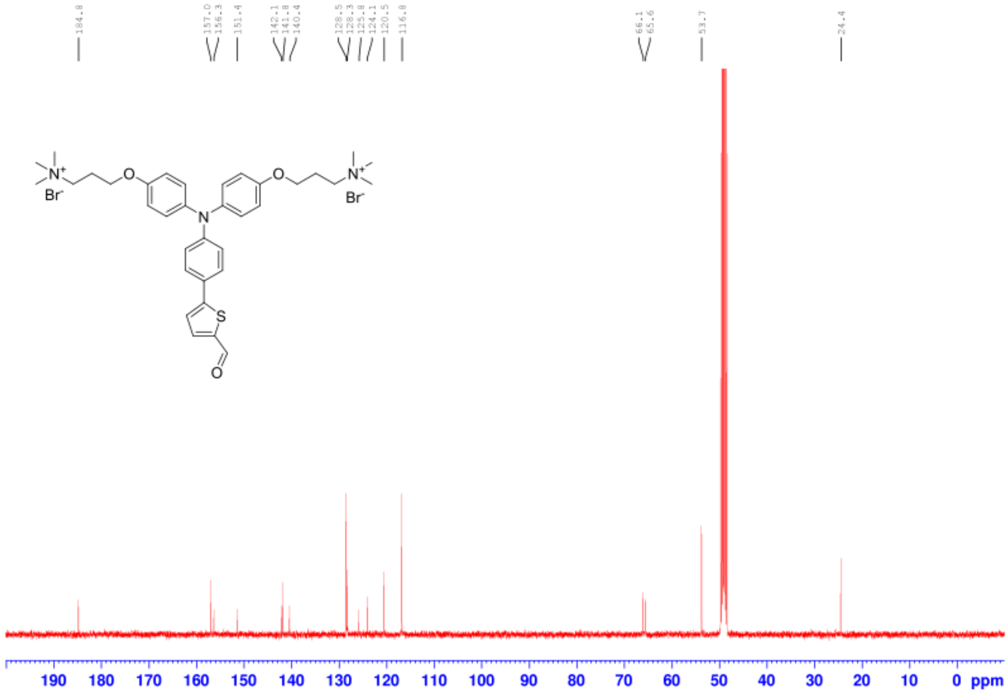

Figure S8. ^{13}C NMR spectrum (100 MHz, MeOD) of compound 4.

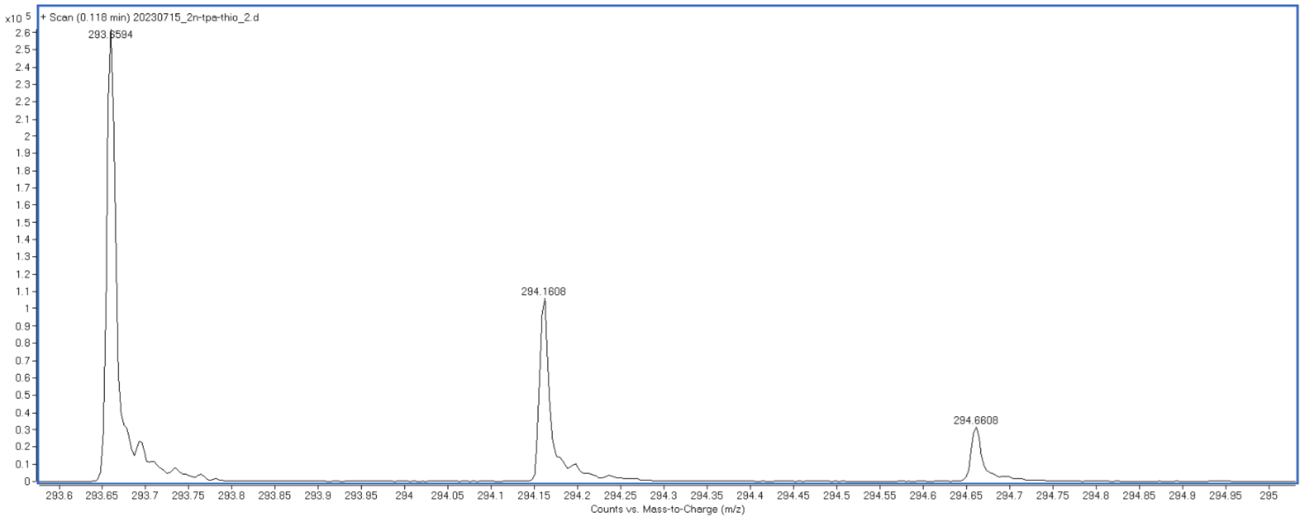

Figure S9. High resolution mass spectrum (ESI) of compound 4.

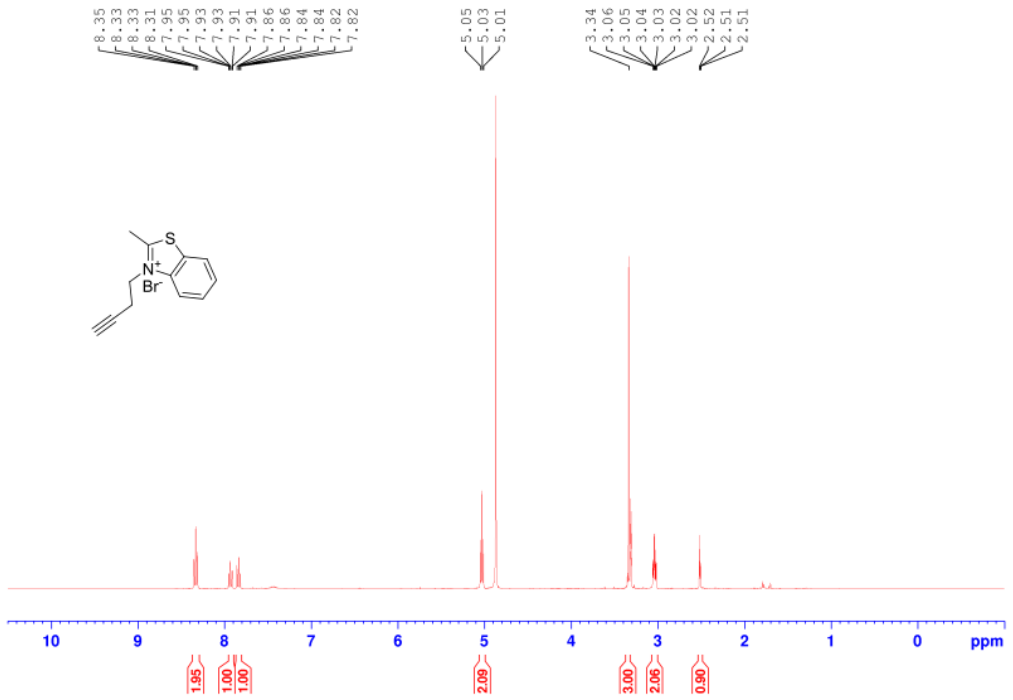

Figure S10. ^{1}H NMR spectrum (400 MHz, MeOD) of M1.

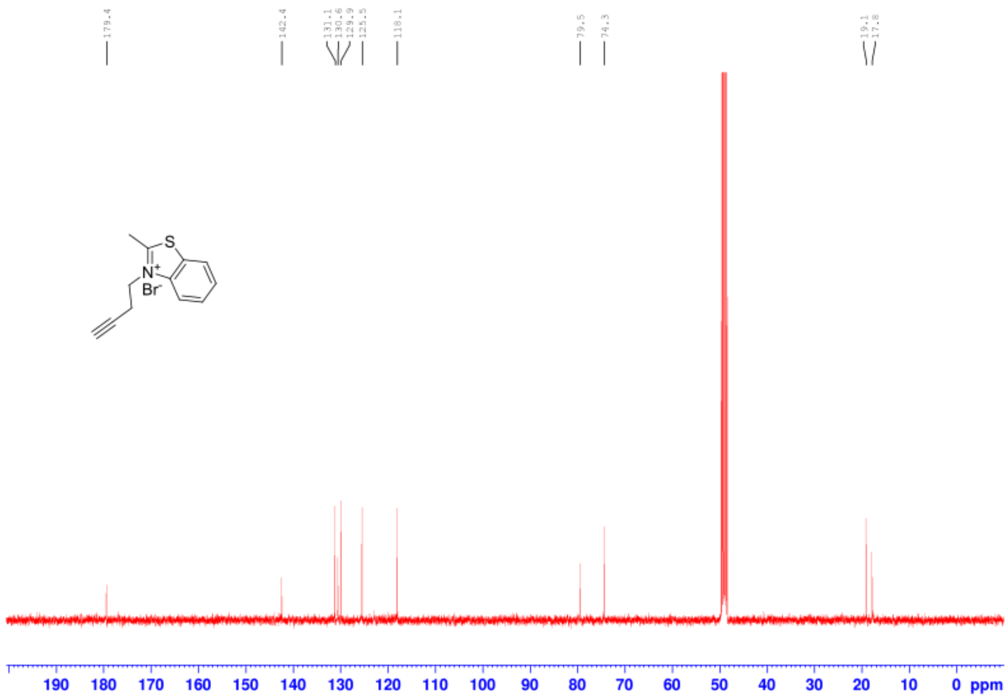

Figure S11. ^{13}C NMR spectrum (100 MHz, MeOD) of compound M1

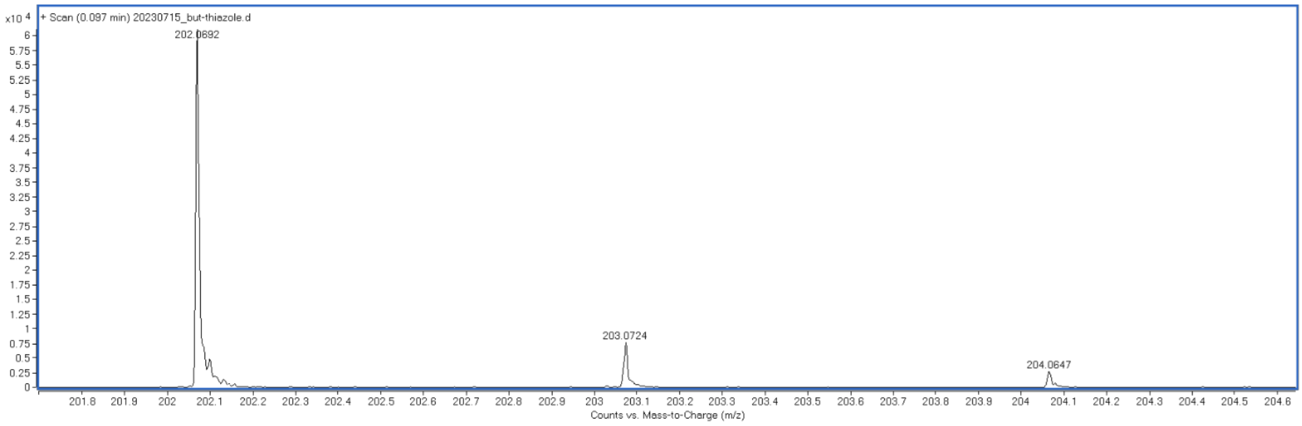

Figure S12. High resolution mass spectrum (ESI) of compound M1

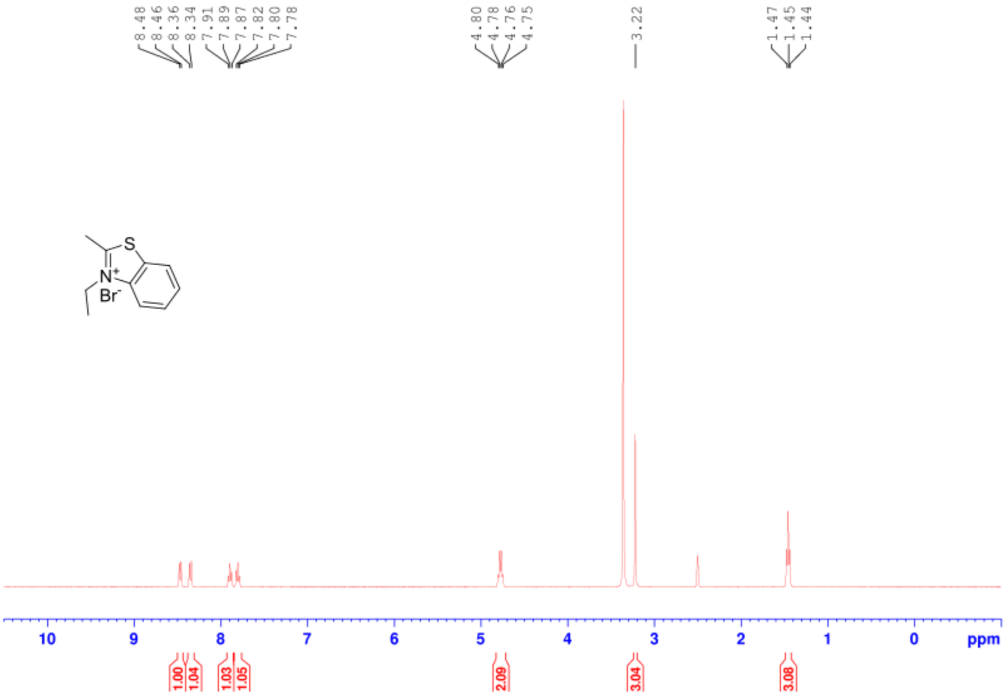

Figure S13. ^{1}H NMR spectrum (400 MHz, DMSO-d_6) of M2.

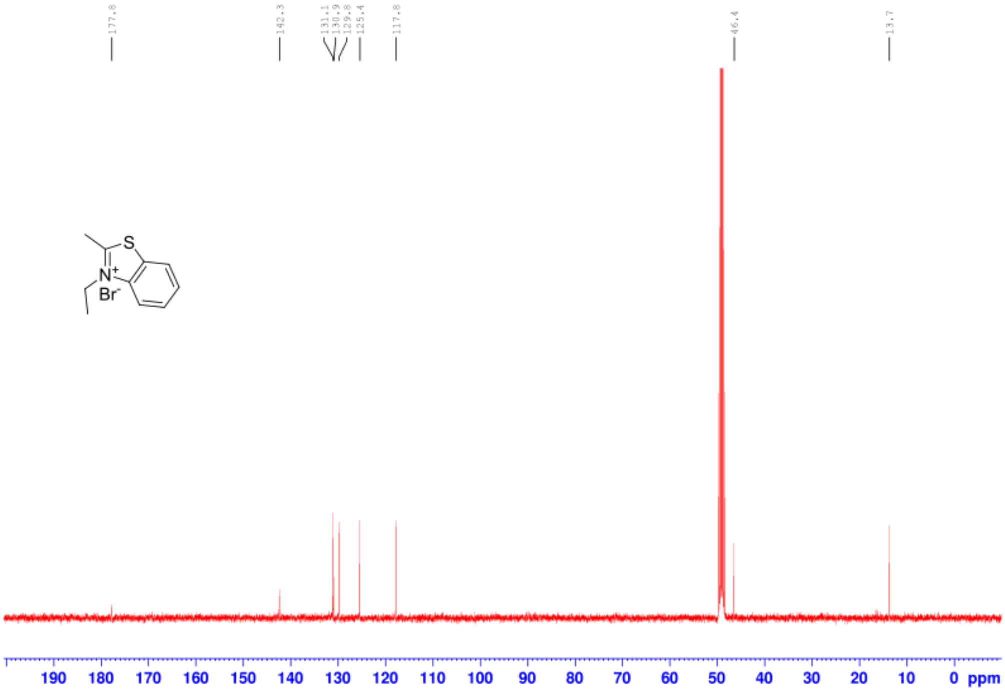

Figure S14. ^{13}C NMR spectrum (100 MHz, MeOD) of M2.

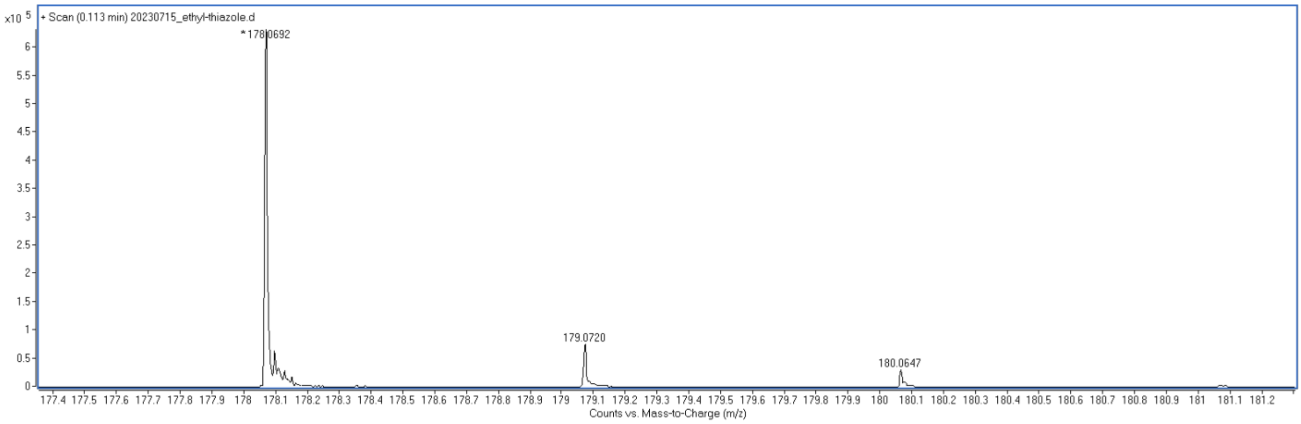

Figure S15. High resolution mass spectrum (ESI) of M2.

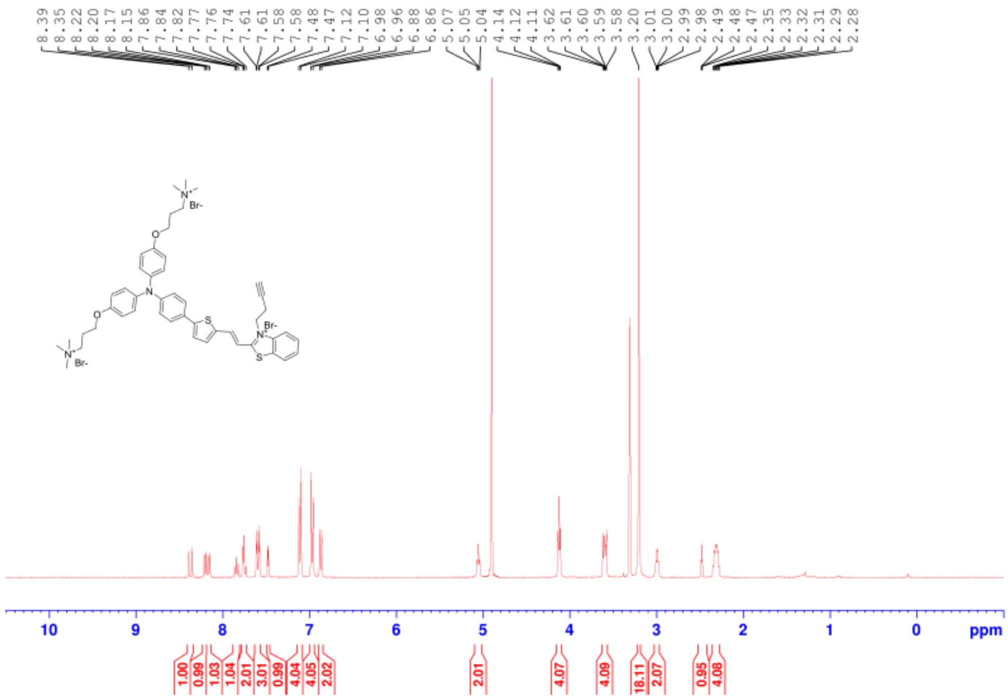

Figure S16. ^{1}H NMR spectrum (400 MHz, MeOD) of TPA-1.

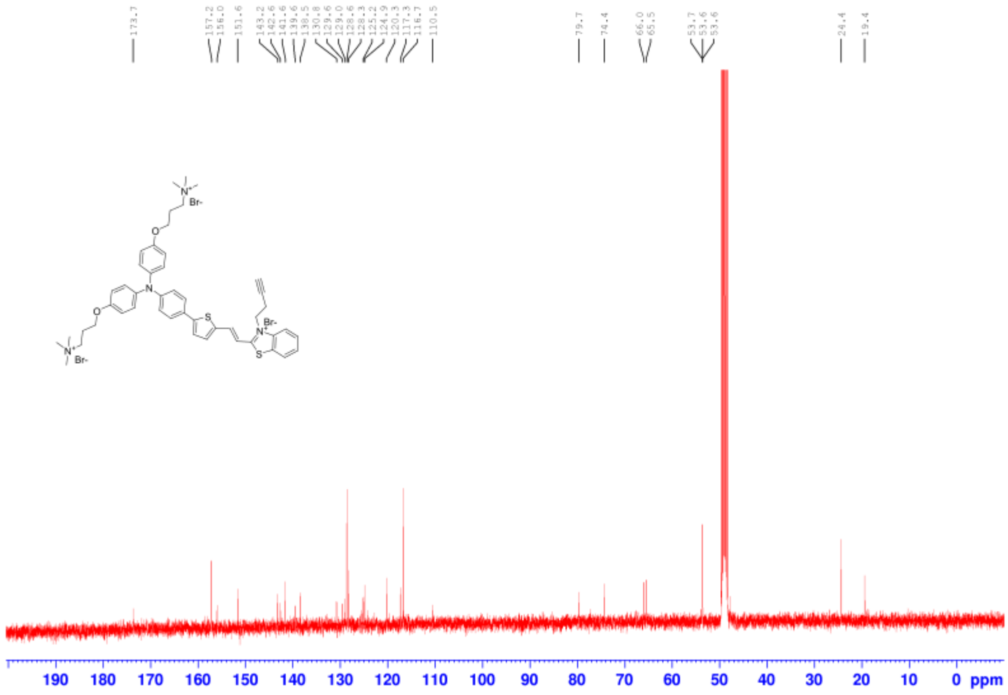

Figure S17. ^{13}C NMR spectrum (100 MHz, MeOD) of TPA-1.

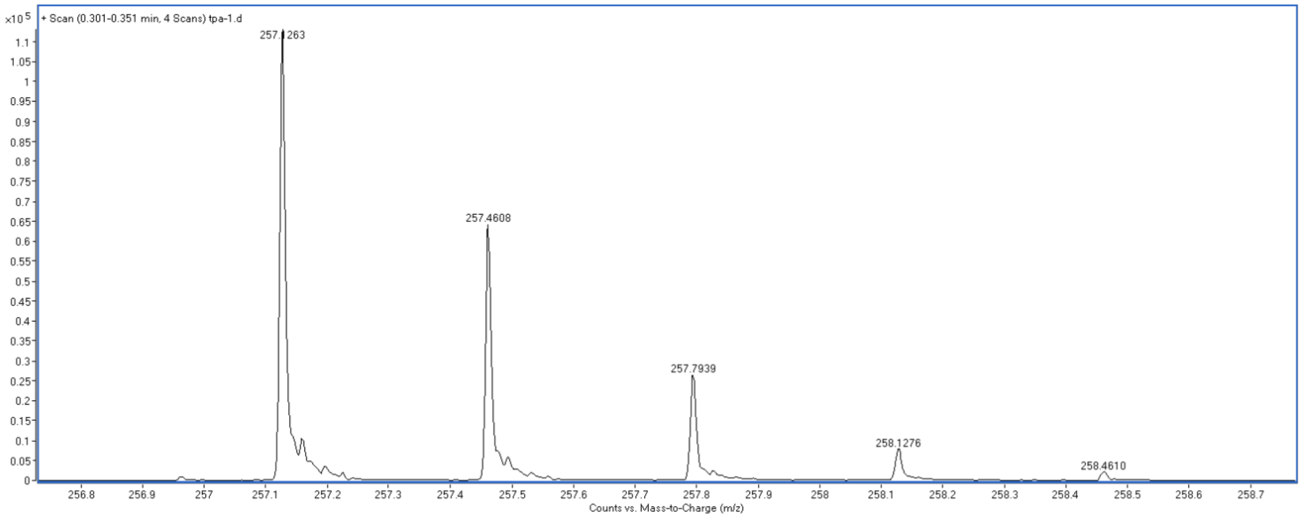

Figure S18. High resolution mass spectrum (ESI) of TPA-1.

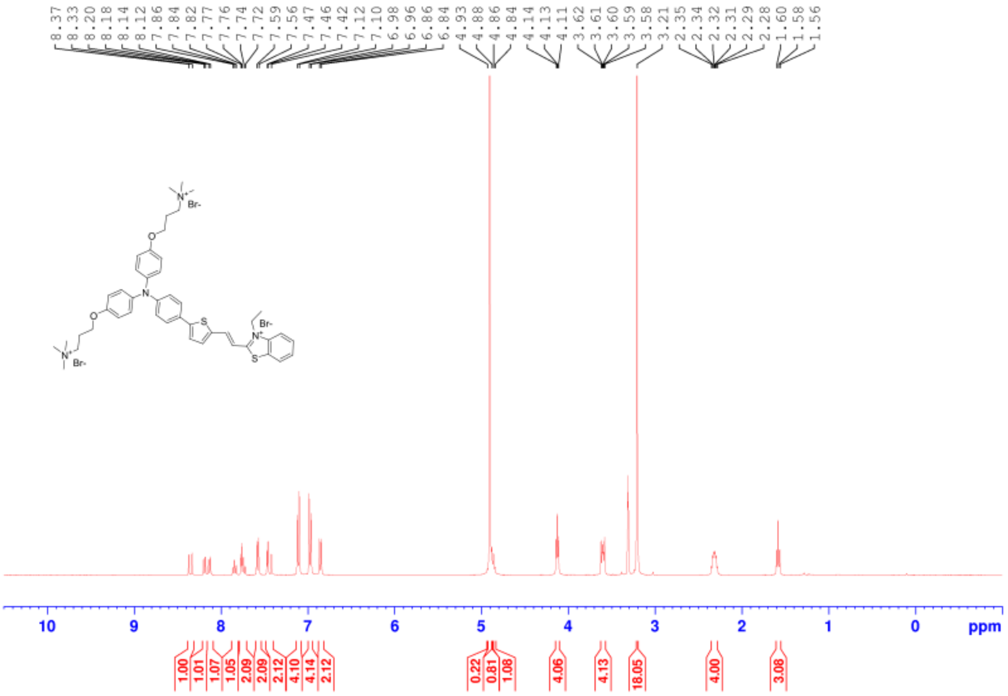

Figure S19. ^{1}H NMR spectrum (400 MHz, MeOD) of TPA-0.

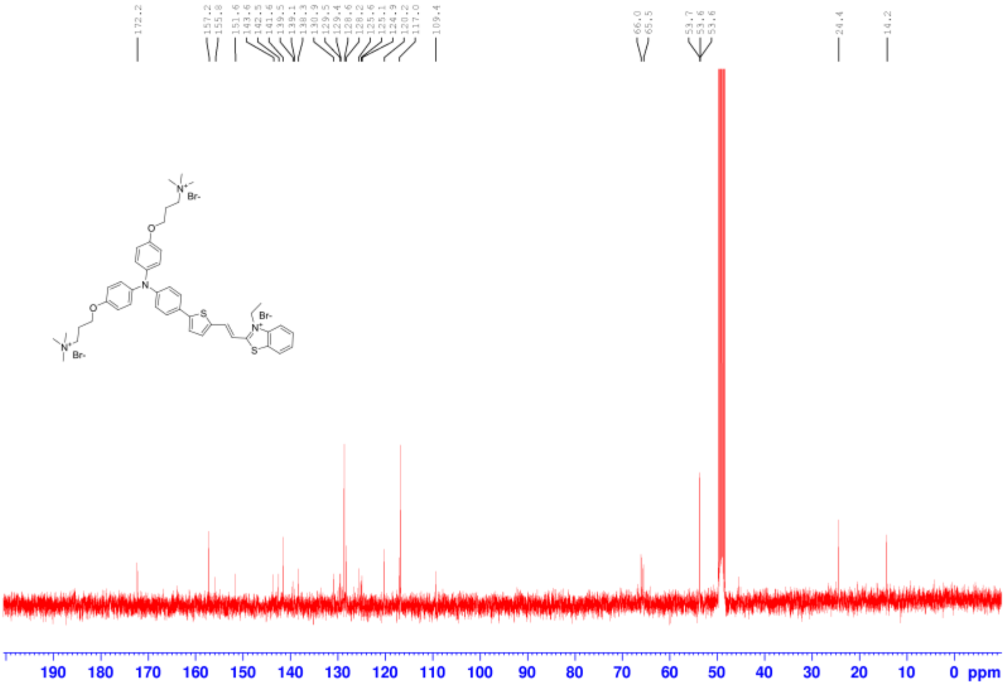

Figure S20. ^{13}C NMR spectrum (100 MHz, MeOD) of TPA-0

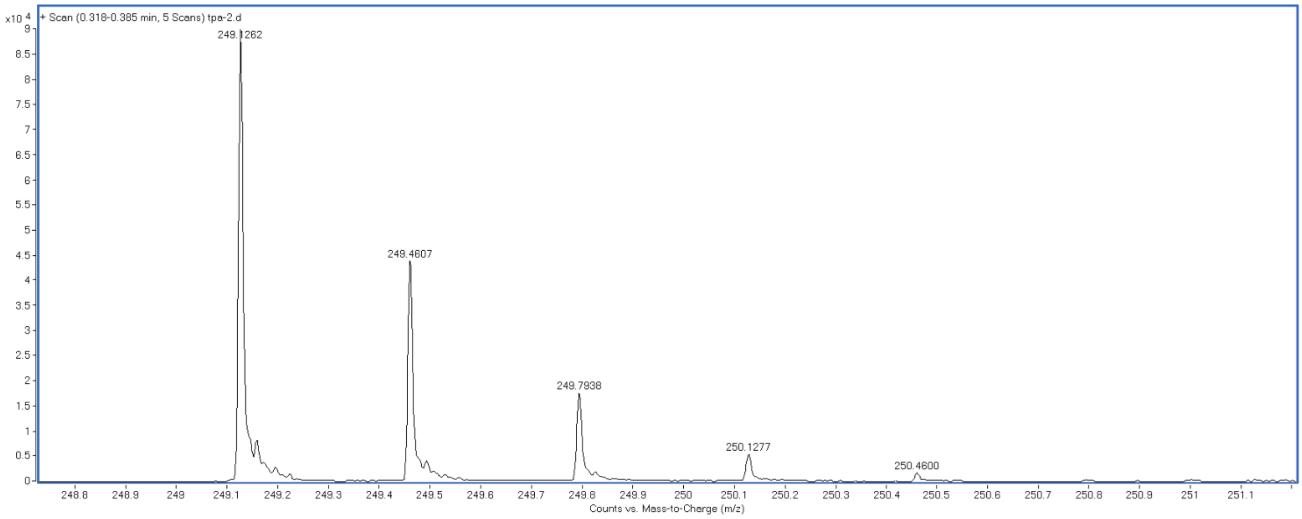

Figure S21. High resolution mass spectrum (ESI) of TPA-0.

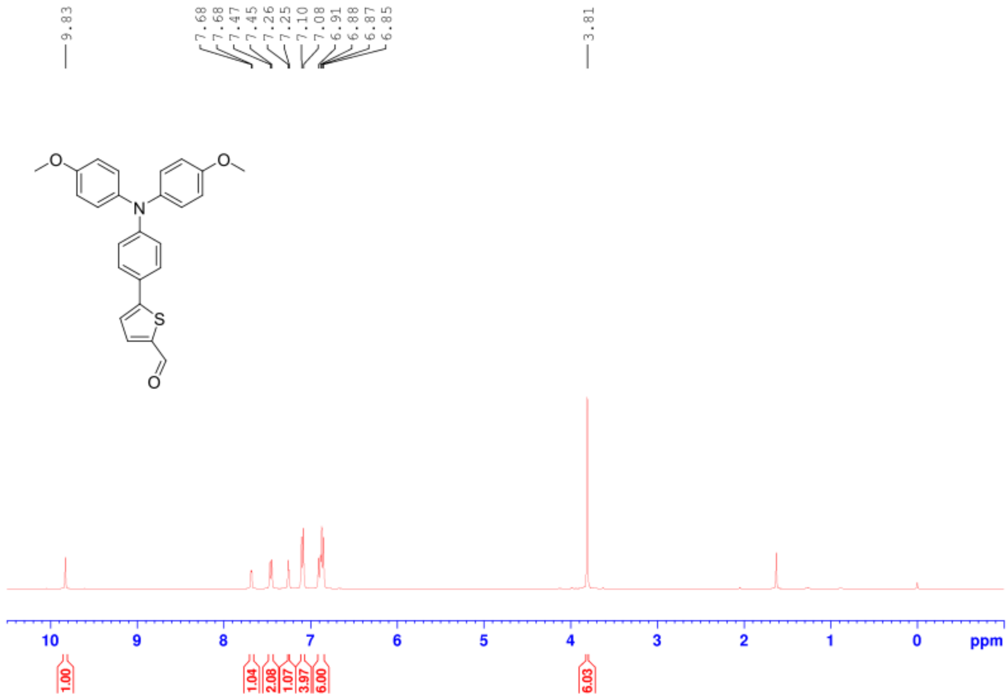

Figure S22. ^{1}H NMR spectrum (400 MHz, $CDCl_3$) of Compound 5.

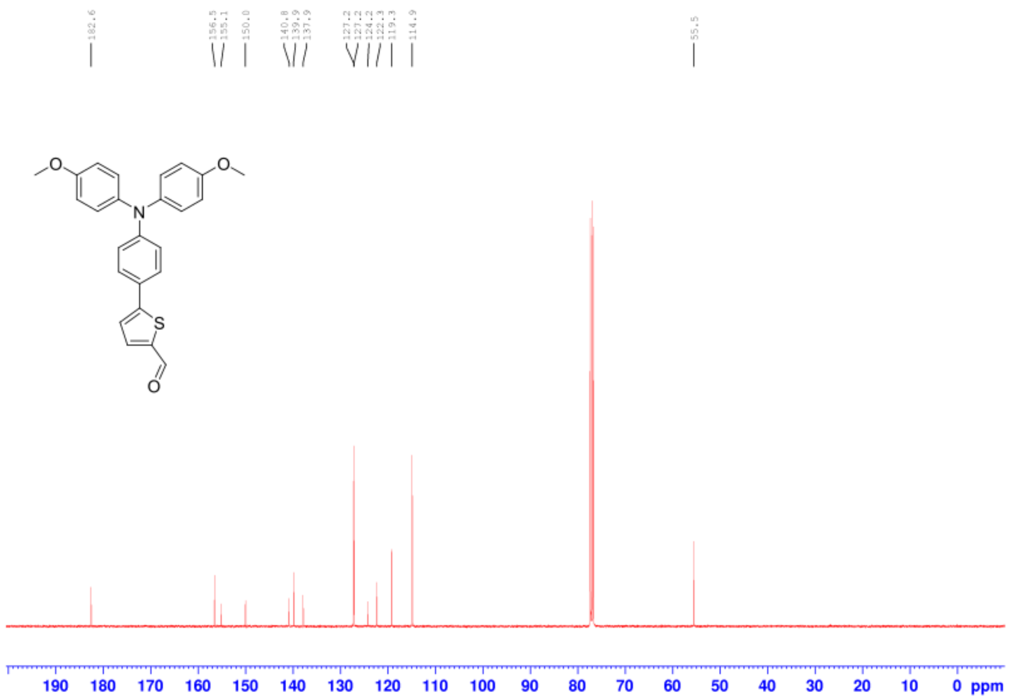

Figure S23. ^{13}C NMR spectrum (100 MHz, $CDCl_3$) of Compound 5.

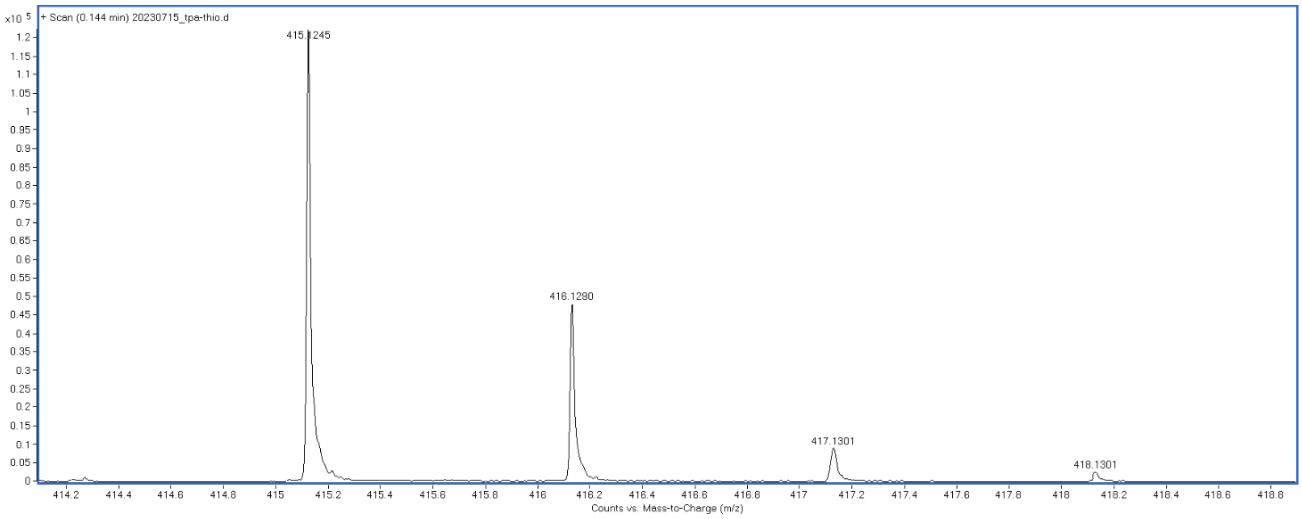

Figure S24. High resolution mass spectrum (ESI) of Compound 5.

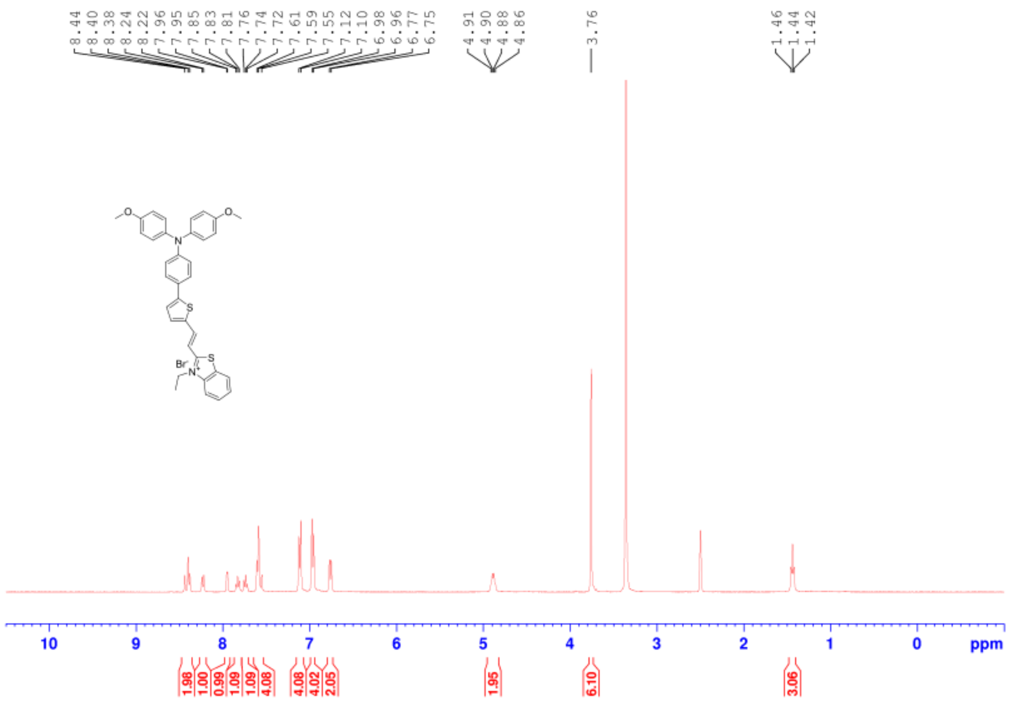

Figure S25. ^{1}H NMR spectrum (400 MHz, DMSO-d_6) of TPA-NC-1.

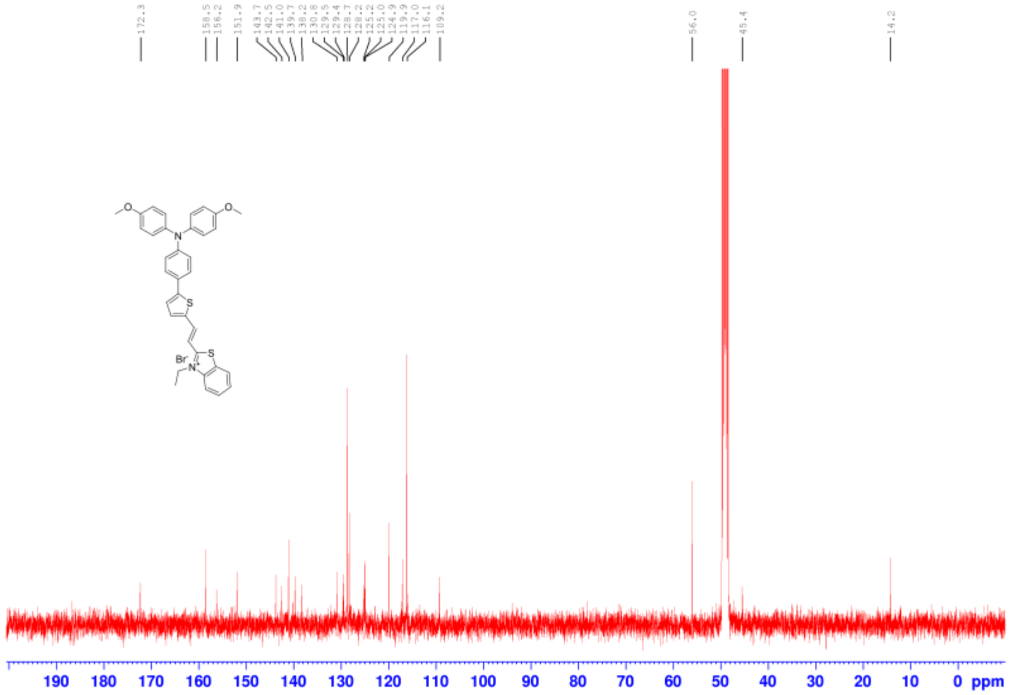

Figure S26. ^{13}C NMR spectrum (100 MHz, MeOD) of TPA-NC-1.

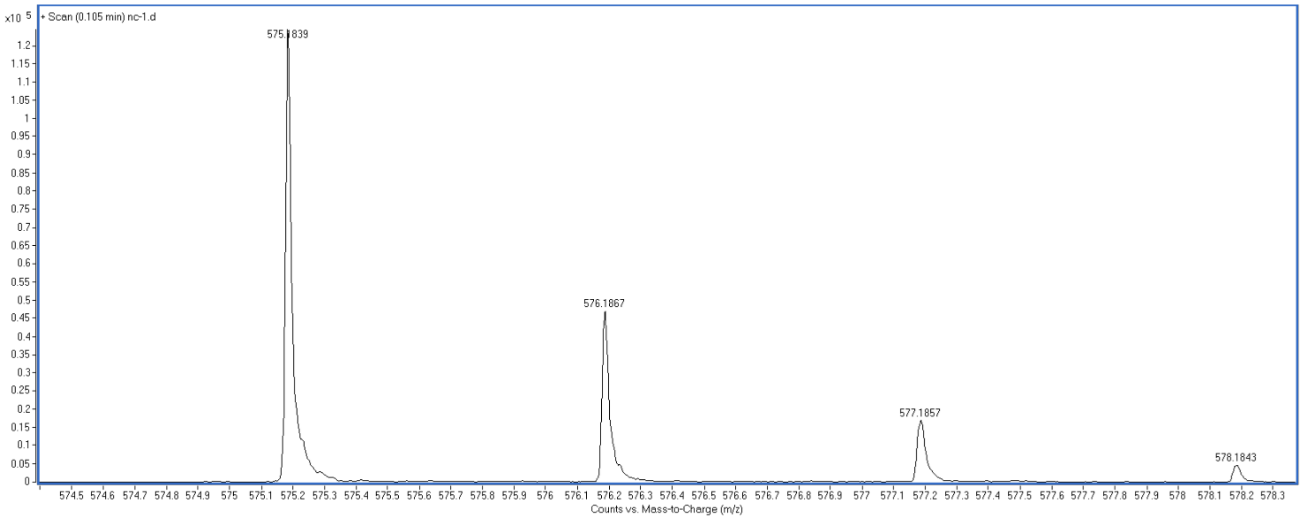

Figure S27. High resolution mass spectrum (ESI) of TPA-NC-1.

3. HPLC Purity Analysis on TPA-NC-1, TPA-0, and TPA-1

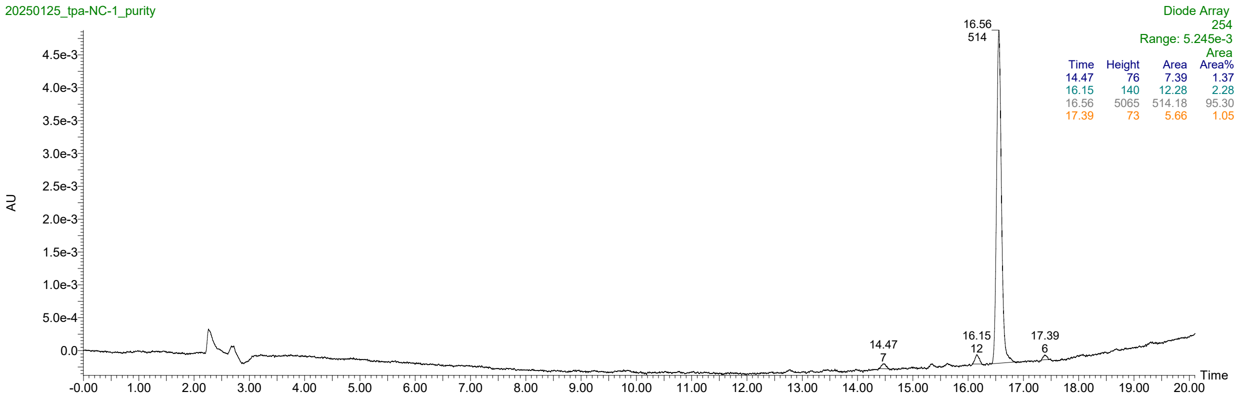

Figure S28. HPLC purity analysis on TPA-NC-1. The elution was applied with water with 0.1% TFA (A) and acetonitrile with 0.1% TFA (B) as follow: isocratic elution at 10% B from 0 - 2 minutes; gradient elution at 10% - 95% B from 2- 20 minutes. The signal was monitored at 254 nm.

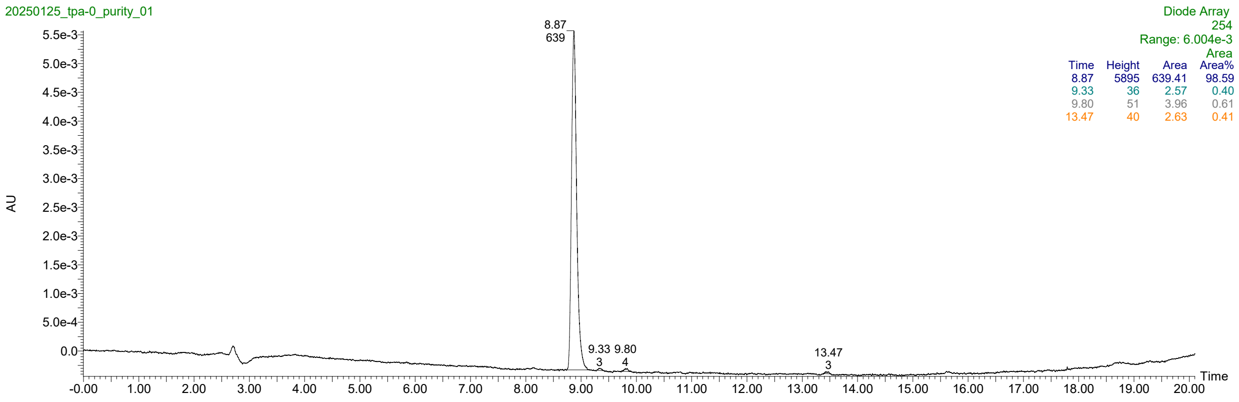

Figure S29. HPLC purity analysis on TPA-0. The elution was applied with water with 0.1% TFA (A) and acetonitrile with 0.1% TFA (B) as follow: isocratic elution at 10% B from 0 - 2 minutes; gradient elution at 10% - 95% B from 2- 20 minutes. The signal was monitored at 254 nm.

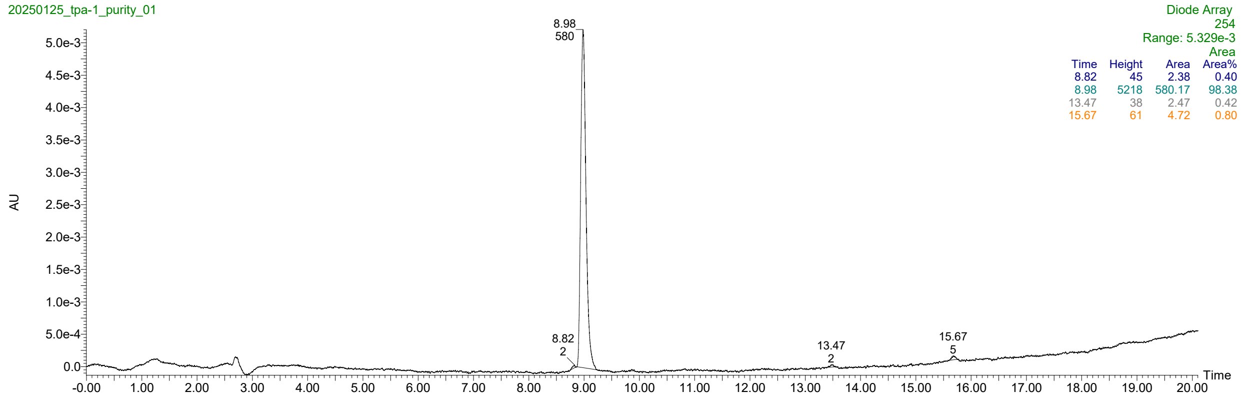

Figure S30. HPLC purity analysis on TPA-1. The elution was applied with water with 0.1% TFA (A) and acetonitrile with 0.1% TFA (B) as follow: isocratic elution at 10% B from 0 - 2 minutes; gradient elution at 10% - 95% B from 2- 20 minutes. The signal was monitored at 254 nm.

4. Absorption Spectra of TPA Compounds and Fluorescence spectra of TPA-1

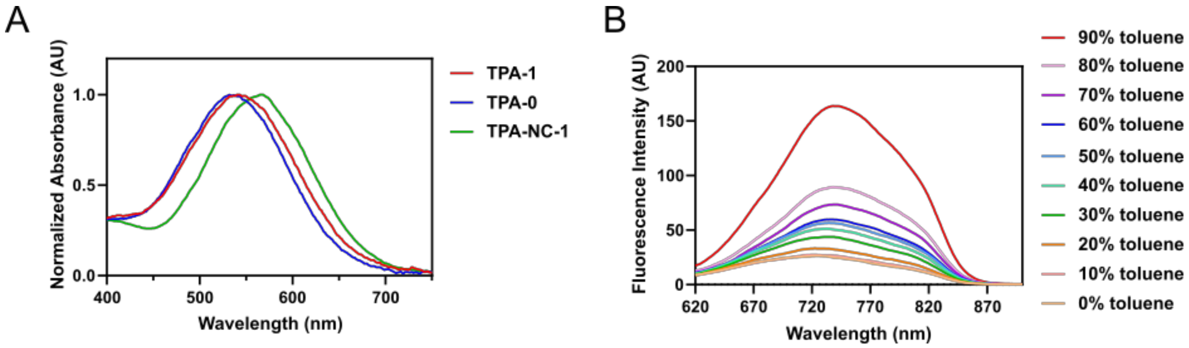

Figure S31. (A) The absorption spectra of TPA-NC-1, TPA-0, and TPA-1 in water. (B) The fluorescence spectra of TPA-1 in methanol with different portions of toluene. The fluorescence intensity increased along with the concentration of toluene, indicating the AIE properties of TPA-1.

5. ROS Generation Assays of TPA-1 and TPA-0 without Light Irradiation

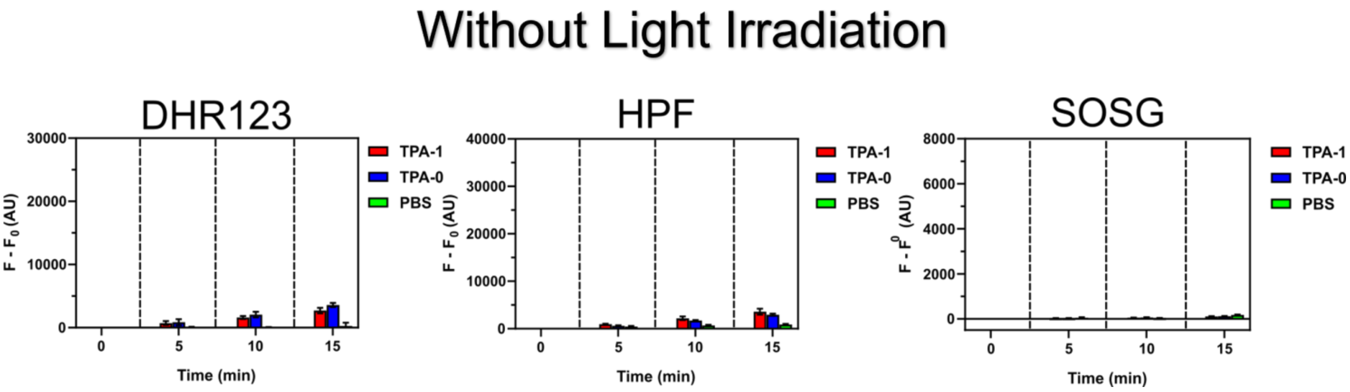

Figure S32. The ROS generation assays of TPA-1 (10 μM) and TPA-0 (10 μM) in the dark for 15 minutes. DHR123 was used as a non-specific ROS detection probe. HPF was used as a hydroxyl radical detection probe. SOSG was used as a singlet oxygen detection probe. $n = 3$ per group.

6. Stability of TPA-1 in pH 6.5-8.5

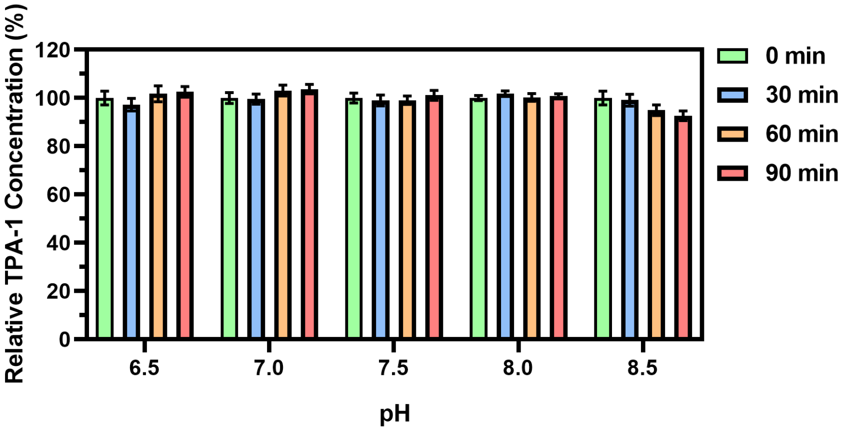

Figure S33. Stability of 50 µM TPA-1 in 0.1 mM Tris-HCl at different pHs over 90 minutes at 37℃. $n = 3$ per group.

7. Antibacterial Abilities of TPA-1 without Light Irradiation

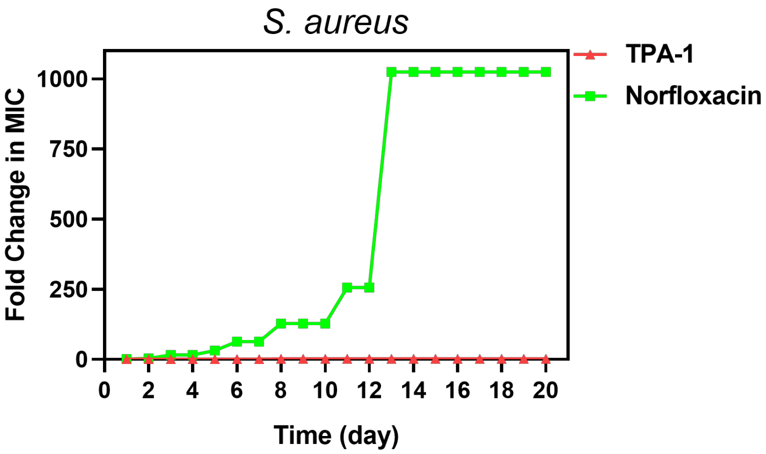

Figure S34. The second replicate of the resistance development assay of TPA-1 and norfloxacin (positive control) on *S. aureus*.

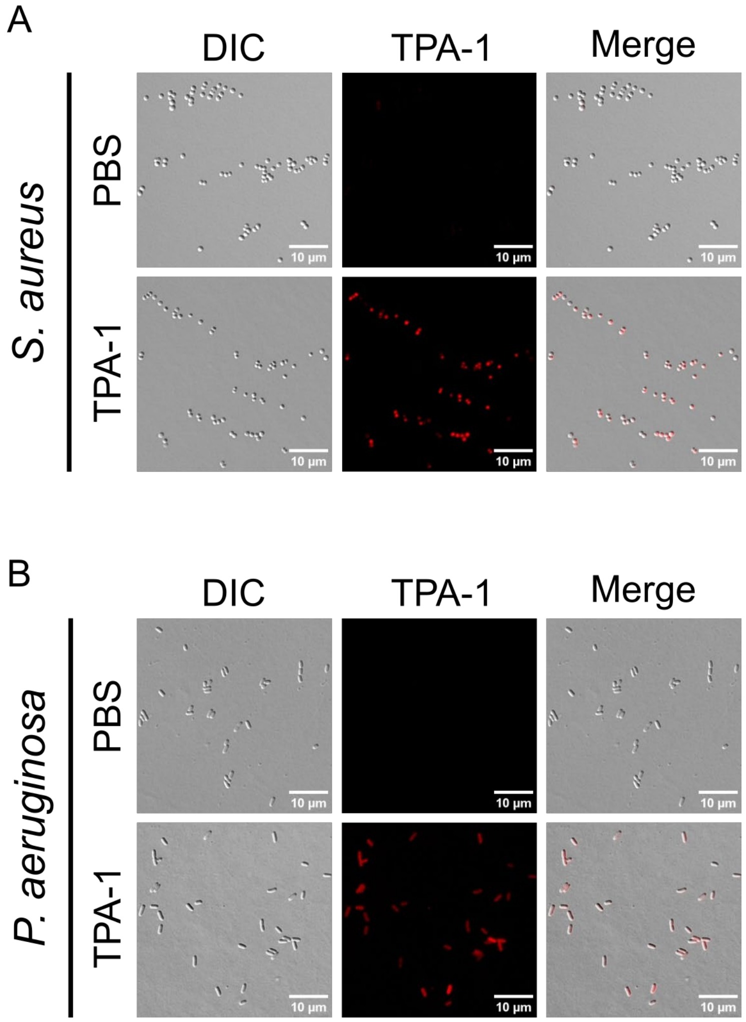

Figure S35. The fluorescence images of (A) *S. aureus* and (B) *P. aeruginosa* after incubation with PBS or 20 μM TPA-1 for 20 minutes at 30 ℃. DIC: differential interference contrast. The excitation wavelength for TPA-1 was 550 nm and the emission wavelength were collected form 590 nm to 670 nm.

Table S1. Minimum inhibition concentrations (MICs) of TPA-1 against *S. aureus*, MRSA and *P. aeruginosa*.

Compound	*S aureus* (ATCC 29213)	Methicillin-resistant *S. aureus* (BAA 41)	*P. aeruginosa* (ATCC 27853)
TPA-1	3.125 μM	3.125 μM	>100 μM
Vancomycin	0.625 μM	0.625 μM	Not determined
Meropenem	0.3125 μM	Not determined	2.5 μM

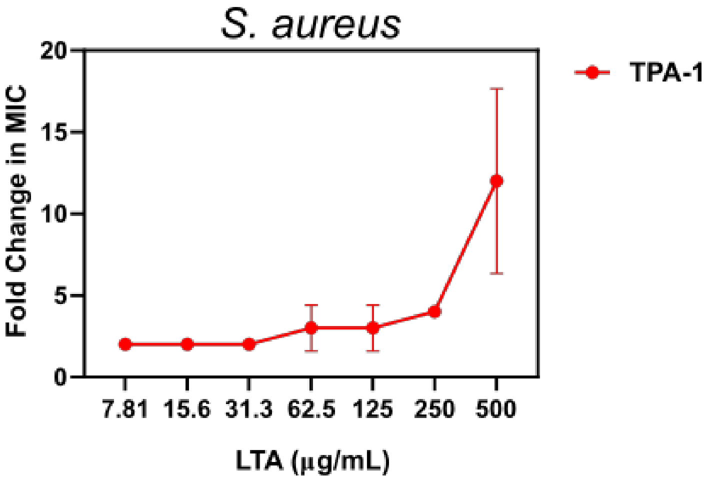

Figure S36. The change in MICs of TPA-1 against *S. aureus* after adding different concentrations of LTA. The MICs of TPA-1 against *S. aureus* increased with the concentration of LTA, suggesting that the antibacterial activity of TPA-1 requires interaction with the LTA on the bacterial membrane. *n* = 2 per group.

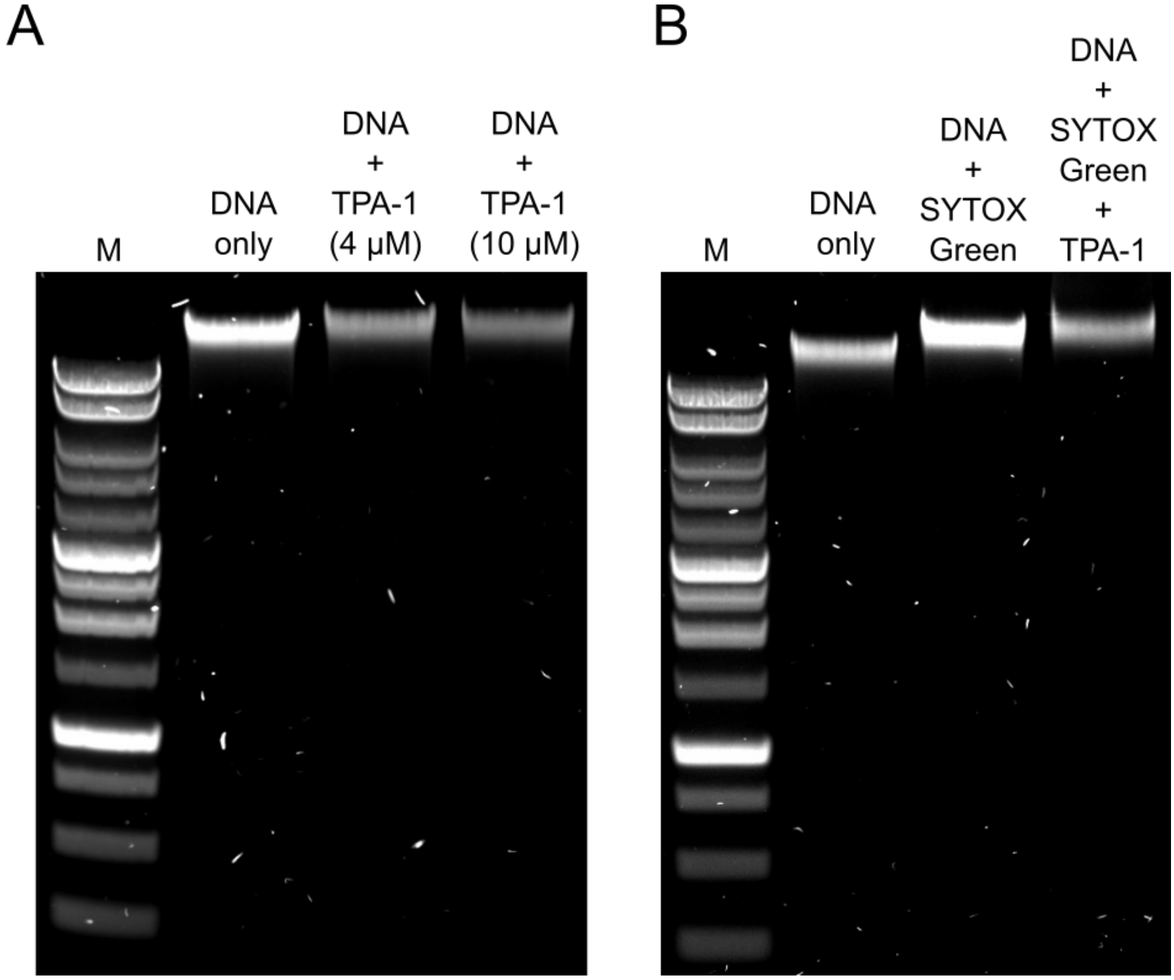

Figure S37. (A) The DNA gel (0.8% agarose) image of different concentrations of TPA-1 mixed with the genomic DNA of *S. aureus*. Upon the addition of TPA-1, the DNA bands were upshifted, which suggested the binding of TPA-1 to DNA. (B) The gel image of SYTOX Green (5 μM) and SYTOX Green (5 μM) + TPA-1 (5 μM) mixed with the genomic DNA of *S. aureus*. SYTOX Green is a commercial DNA stain (positive control). M represented the DNA marker.

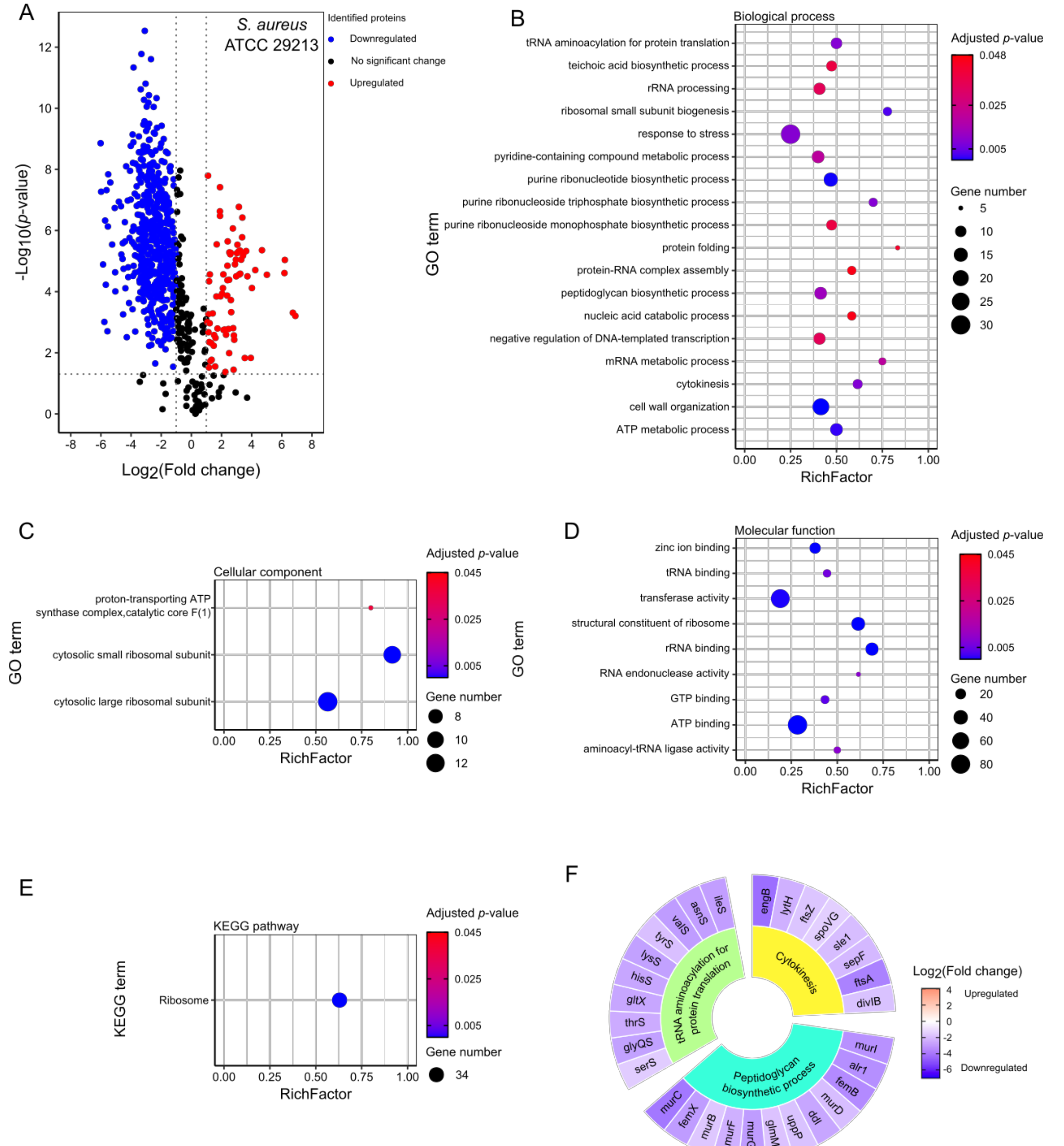

Figure S38. (A) The volcano plot showed the differentially expressed proteins (DEP)s in TPA-1 (1 × MIC, 3.125 μM) treated *S. aureus* versus control *S. aureus* after an hour of incubation at 37℃ in the dark. (B) Biological function, (C) cellular component, (D) molecular function GO enrichment, and (E) KEGG pathway enrichment analysis of the corresponding genes of the DEPs in TPA-1-treated *S. aureus*. (F) Selected biological processes in *S. aureus* that were affected by TPA-1. Inner blocks were the names of the selected biological processes. Outer blocks show the fold change of the DEPs, which are represented by their gene names, in the corresponding biological processes. Three independent biological replicates were performed in this proteomic study. For both *S. aureus* treated with 1 × MIC or 2 × MIC of TPA-1, the same number and types of proteins were identified in cytokinesis, peptidoglycan biosynthetic process, and tRNA aminoacylation for protein translation. Moreover, the proteins in these three biological processes were more downregulated in *S. aureus* treated with 2 × MIC of TPA-1 than those treated with 1 × MIC, suggesting that the downregulation of these DEPs depends on the concentration of TPA-1.

8. Antibiofilm Abilities of TPA-1 with Light Irradiation

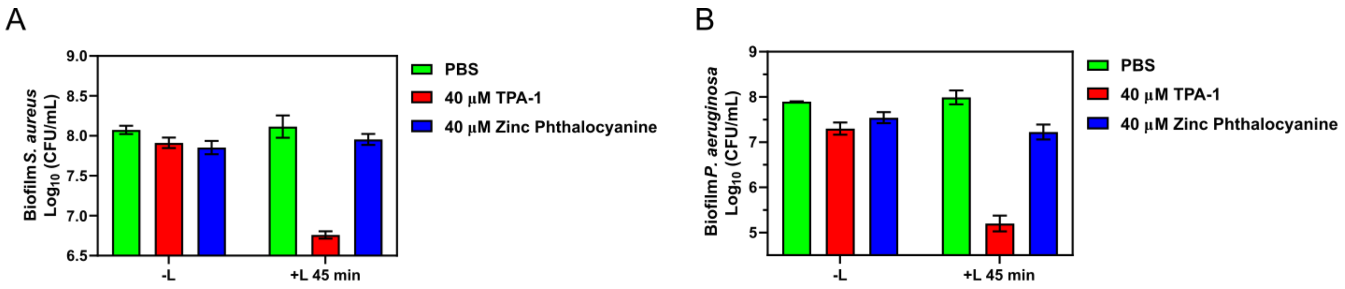

Figure S39. The number of viable bacterial cells in (A) *S. aureus* biofilm and (B) *P. aeruginosa* biofilm after PDT with zinc phthalocyanine. n = 3 per group. -L: Without light irradiation. +L: With 600 nm (60 mW/cm^2) light irradiation.

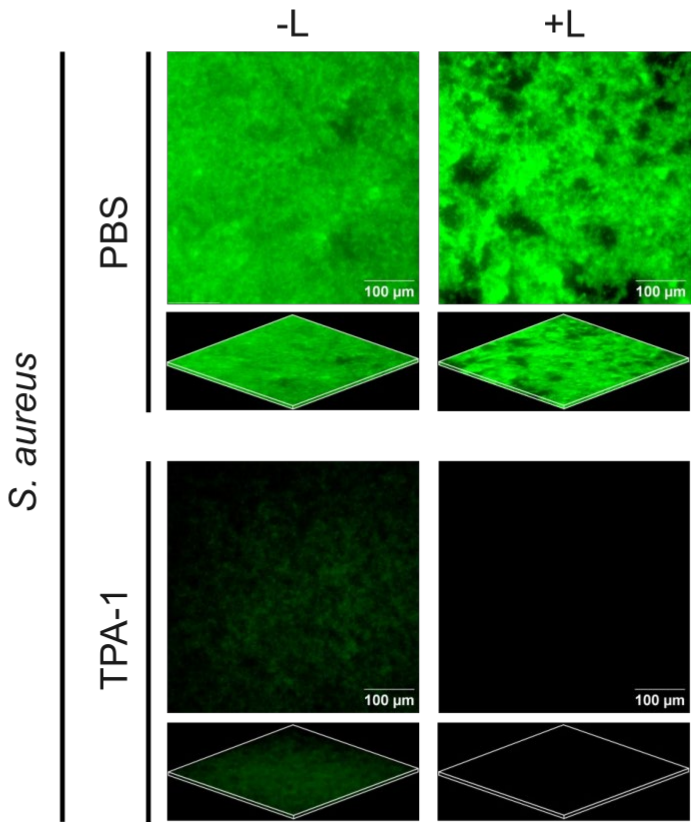

Figure S40. The fluorescence images of *S. aureus* biofilm after various treatments. The biofilm was stained by Calcein-AM, which can generate a green fluorescence signal inside a metabolic active cell. The excitation wavelength was 490 nm. Emission wavelength was collected from 500 to 550 nm. -L: Without light irradiation. +L: With 600 nm (60 mW/cm^2) light irradiation.

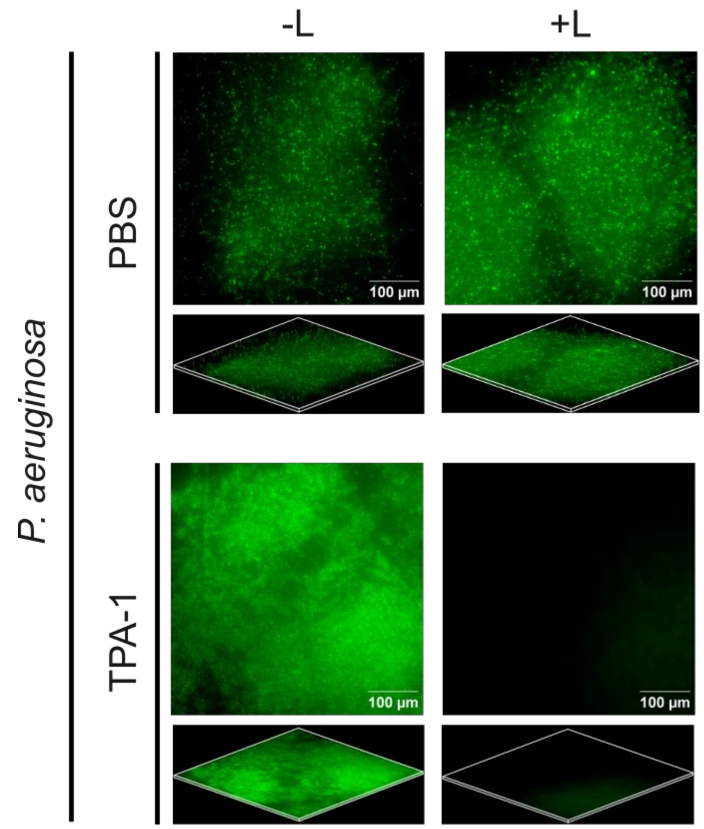

Figure S41. The fluorescence images of *P. aeruginosa* biofilm after various treatments. The biofilm was stained by Calcein-AM, which can generate a green fluorescence signal inside a metabolic active cell. The excitation wavelength was 490 nm. Emission wavelength was collected from 500 to 550 nm. -L: Without light irradiation. +L: With 600 nm (60 mW/cm^2) light irradiation.

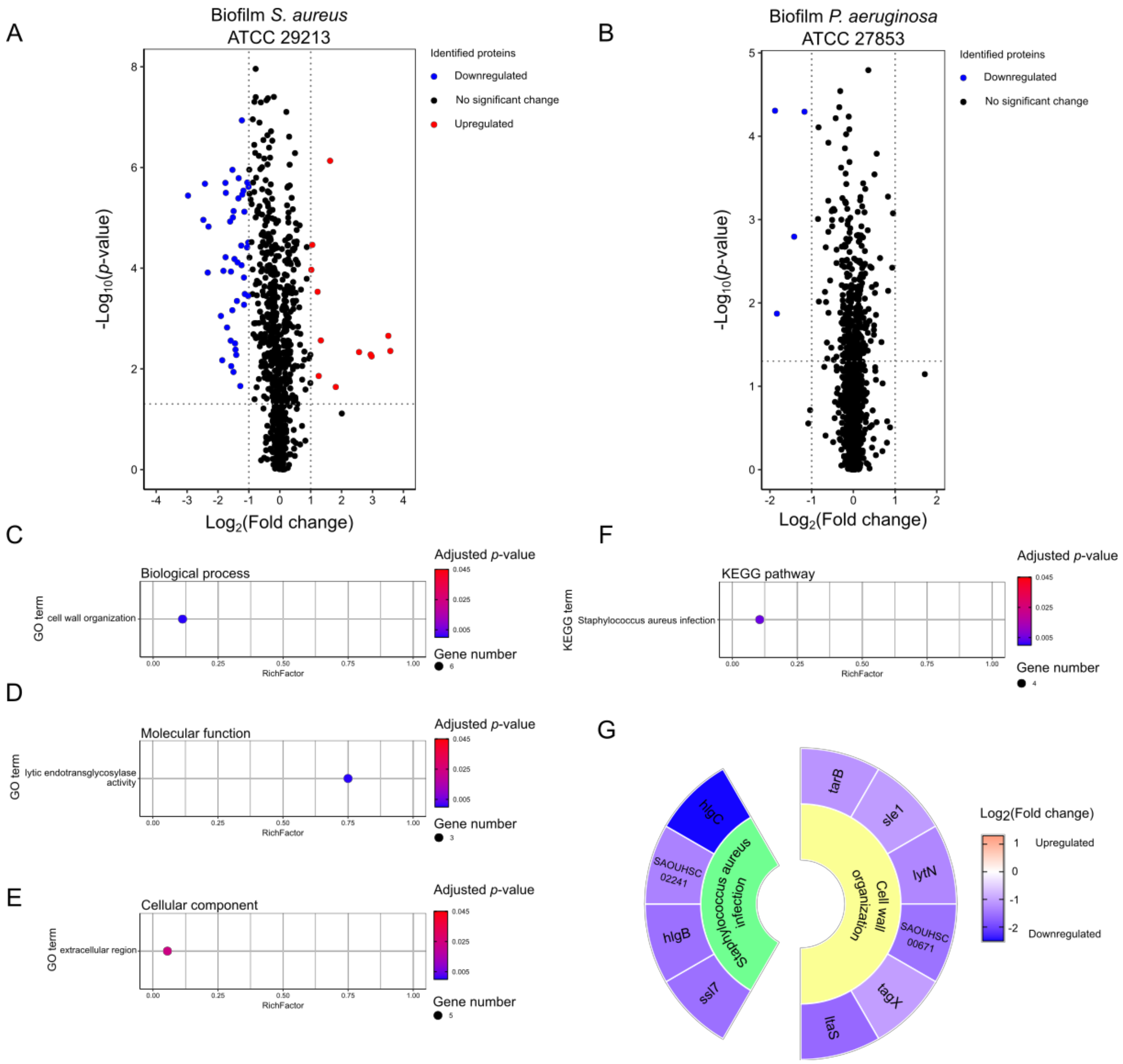

Figure S42. (A) The volcano plot showed the DEPs in TPA-1 (40 µM)-treated biofilm *S. aureus* versus PBS-treated biofilm *S. aureus* and (B) TPA-1 (40 µM)-treated biofilm *P. aeruginosa* versus PBS-treated biofilm *P. aeruginosa* after 45 minutes of 600 nm (60 mW/cm^2) irradiation. (C) Biological process, (D) molecular function, (E) cellular component GO enrichment, and (F) KEGG pathway enrichment analysis of the corresponding genes of the DEPs in TPA-1-treated biofilm *S. aureus* after light irradiation. (G) Selected biological processes and KEGG pathway in biofilm *S. aureus* after PDT of TPA-1. Inner blocks were the names of the selected biological process and KEGG pathway. Outer blocks show the fold change of the DEPs, which are represented by their gene names, in the corresponding biological process and KEGG pathway. Three independent biological replicates were performed in this proteomic study.

9. Selectivity Assays of TPA-1

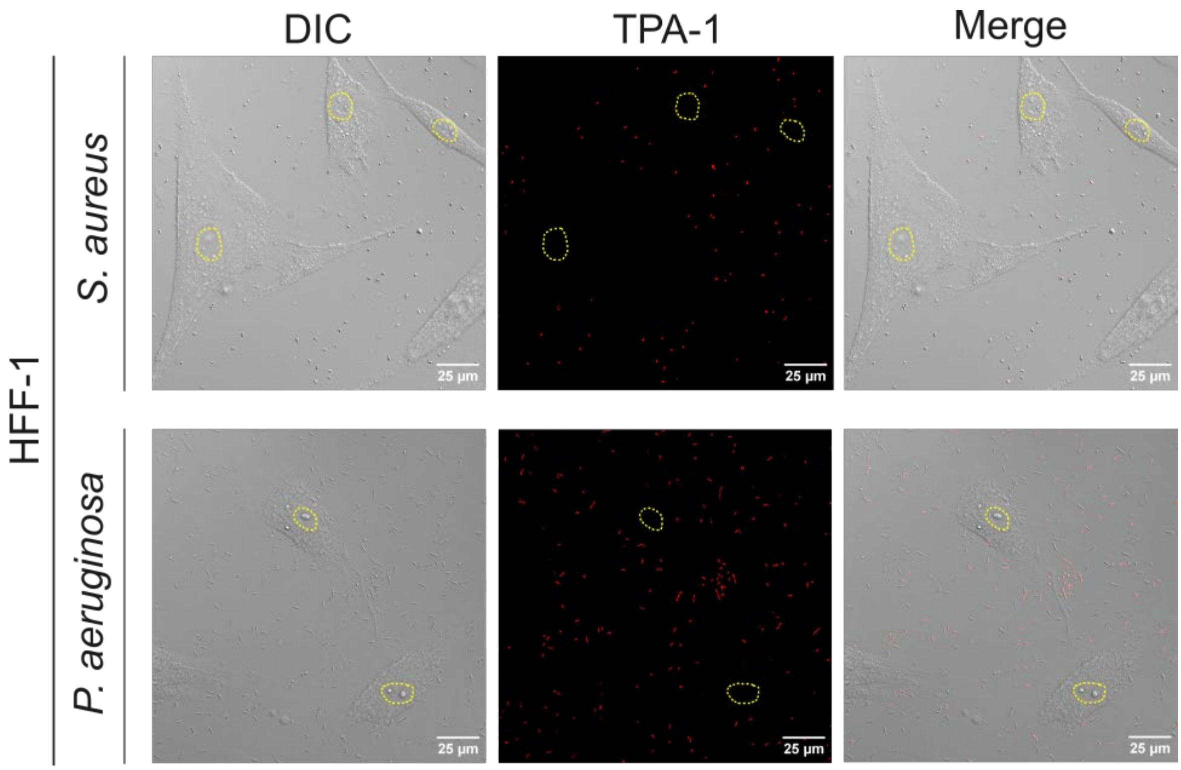

Figure S43. The fluorescence images of HFF-1 cells (50000 cells/well) were mixed with 1× 10^8 CFU/mL *S. aureus* or *P. aeruginosa* and incubated with TPA-1 (10 μM). The yellow dotted circles represent the nuclei of the HFF-1 cells. TPA-1 can specifically label bacteria. The excitation wavelength was 550 nm. Emission wavelength was collected from 590 to 670 nm.

10. *In vivo* Assays

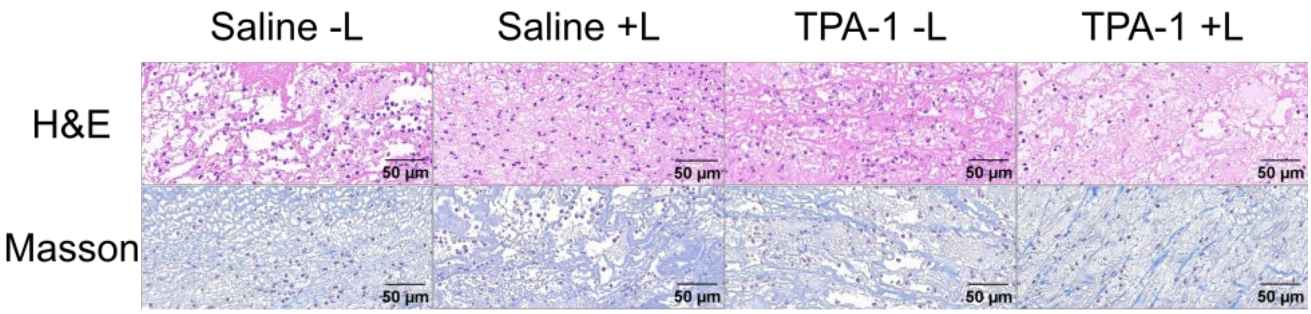

Figure S44. H&E and Masson's trichrome stained slide of MRSA biofilm infected tissue after 4 days of treatments. -L: Without light irradiation. +L: With 600 nm (60 mW/cm^2) light irradiation for 15 minutes.

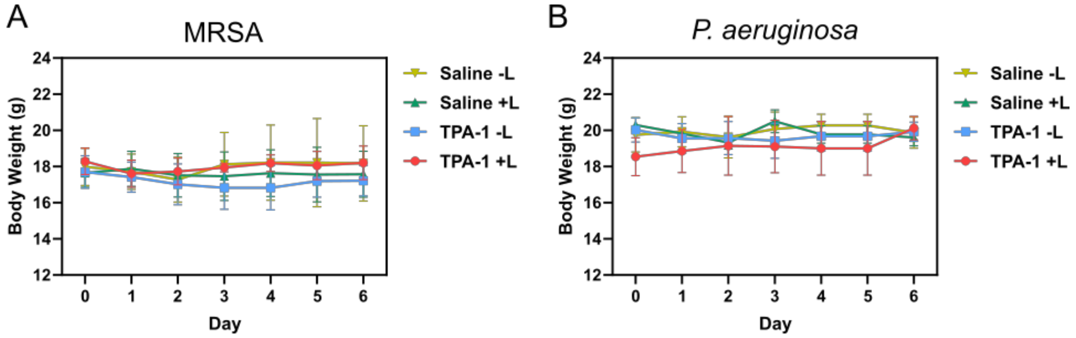

Figure S45. The body weight of (A) MRSA and (B) *P. aeruginosa* biofilm infected mice throughout the experiment. n = 8 per group for (A), n = 4 per group for (B).
